# In silico reversal of repeat-induced point mutation (RIP) identifies the origins of repeat families and uncovers obscured duplicated genes

**DOI:** 10.1186/1471-2164-11-655

**Published:** 2010-11-24

**Authors:** James K Hane, Richard P Oliver

**Affiliations:** 1 Faculty of Health Sciences, Murdoch University, Perth, Western Australia, 6150, Australia; 2 Department of Environment and Agriculture, Curtin University, Perth, Western Australia, 6102, Australia; 3 Current address: CSIRO Plant Industry, CELS Floreat, Perth, Western Australia, 6014, Australia

## Abstract

**Background:**

Repeat-induced point mutation (RIP) is a fungal genome defence mechanism guarding against transposon invasion. RIP mutates the sequence of repeated DNA and over time renders the affected regions unrecognisable by similarity search tools such as BLAST.

**Results:**

DeRIP is a new software tool developed to predict the original sequence of a RIP-mutated region prior to the occurrence of RIP. In this study, we apply deRIP to the genome of the wheat pathogen *Stagonospora nodorum *SN15 and predict the origin of several previously uncharacterised classes of repetitive DNA.

**Conclusions:**

Five new classes of transposon repeats and four classes of endogenous gene repeats were identified after deRIP. The deRIP process is a new tool for fungal genomics that facilitates the identification and understanding of the role and origin of fungal repetitive DNA. DeRIP is open-source and is available as part of the RIPCAL suite at http://www.sourceforge.net/projects/ripcal.

## Background

Repeat-induced point mutation (RIP) is a genome defence mechanism found within filamentous ascomycete fungi that is purported to combat transposon invasion. RIP mutates duplicated DNA sequences during sexual reproduction, thereby inactivating genes encoded in both copies. First discovered in *Neurospora crassa *[1,2], RIP was later demonstrated in the Ascomycetes *Magnaporthe oryzae *[3,4], *Podospora anserina *[5], *Leptosphaeria maculans *[6] and *Fusarium graminearum *[7]. Putative RIP events have also been detected bioinformatically in *Aspergillus fumigatus *[8], *Fusarium oxysporum *[9-11], *Aspergillus nidulans *[12], *Neurospora tetrasperma *[13], *Microbotryum violaceum *[14], *Aspergillus oryzae *[15], *Magnaporthe oryzae *[16], *Colletotrichum cereal *[17], *Aspergillus niger *[18], *Penicillium chysogenum *[18] and most recently in *Stagonospora nodorum *[19,20]. Given this broad distribution, it is reasonable to assume that RIP is widespread across, but so far restricted to, filamentous ascomycota and basidiomycota.

The mechanism by which RIP operates is yet to be fully understood, but the following observations have been made. RIP involves transition mutations from C:G to T:A nucleotide base pairs in duplicated DNA; this affects both copies of the repeat and occurs prior to meiosis [1,2]. In the majority of cases studied so far, there is a strong bias for the mutation of C:G nucleotide base pairs followed by A:T nucleotide base pairs [18,21,22]. Thus CpA di-nucleotides are more frequently affected than any of the other 15 di-nucleotides. CpA nucleotides are converted to TpA. Coincidentally, the complementary TpG di-nucleotide on the opposite strand is also converted to TpA (Table 1). In *N. crassa*, RIP requires ≥ 80% identity of duplicated DNA over a length of ≥ 400 bp [23,24].

**Table 1:** The four potential di-nucleotide RIP mutations detected by RIPCAL.

RIP mutation				Counted di-nucleotides	
Forward		Reverse complement		Forward	Reverse complement
		
pre-RIP	post-RIP	pre-RIP	post-RIP		
CpA	TpA	TpG	TpA	CpA, TpA	TpG, TpA
CpC	TpC	GpG	GpA	CpC, TpC	GpG, GpA
CpG	TpG	CpG	CpA	CpG, TpG	CpG, CpA
CpT	TpT	ApA	ApG	CpT, TpT	ApG, ApA

The deRIP process counts the occurrence of the contributing di-nucleotides incrementally across a multiple alignment of repeats and alters the consensus sequence at each position to the appropriate pre-RIP di-nucleotide sequence.

The consequences of RIP are that repeated DNA segments, such as would result from the transposition of a retrotransposon, or the duplication of a gene, are mutated and inactivated. RIP would be expected to operate in successive sexual cycles until the sequence identity between duplicated sequences is reduced below the minimum homology threshold required by the RIP machinery. The genome would then contain a repeat family consisting of relics of the duplication event degraded to varying degrees.

The rapid increase in the number of fungal genome assemblies has created a demand for methods to detect and quantify RIP. Two approaches have been used; RIP indices and alignment methods. RIP increases the frequency of particular di-nucleotides (TpA in most cases studied to date) in affected regions of DNA. Thus RIP can be identified by comparing ratios of di-nucleotide frequencies in pre-RIP to post-RIP sequences; these ratios are referred to as "RIP indices" [8,12,25,26]. However, in reality RIP depends upon the alignment of two similar regions of double-stranded DNA [23] and therefore it is more appropriate to use alignments of repeat families to identify and quantify RIP. We have previously introduced a rapid, automated alignment-based procedure for the whole-genome analysis of RIP mutation called RIPCAL [20]. Using this procedure, we readily identified and quantified the degree of RIP in all repeated DNA families within the genome of the necrotrophic fungal wheat pathogen *S. nodorum*.

*Stagonospora *(syn. *Septoria*) *nodorum *[teleomorph: *Phaeosphaeria *(syn. *Leptosphaeria*) *nodorum *(Müll) Hedjar.] is a major pathogen of wheat and is a model for the fungal class Dothideomycetes, a taxon that includes many important pathogens of crops [27]. *S. nodorum *infects wheat crops in most wheat-growing areas of the world [28]. Infection is predominantly determined by the presence of various effectors (host-specific toxins) harboured by different strains of the fungus [29]. The fungus is heterothallic (out-crossing) and the mating types are evenly distributed [30]. The fungus over-summers as ascospores on stubble [28] and multiplies via asexual reproduction during the growing season. The pathogen displays high levels of variability as determined by genomic analyses [31,32] and this has been exploited to determine the biogeographic history of the pathogen [33]. The pattern of micro-satellite markers is consistent with a pattern whereby the pathogen originated in the "Golden Triangle" region and spread as wheat cultivation was adopted in Eurasia and North Africa several thousand years ago and into North and South America, South Africa and Australia since European colonisation.

**Figure 1:**
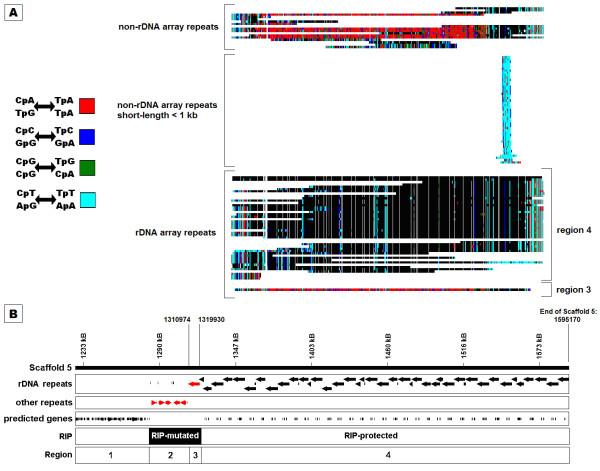
**The distribution of the Y1 family of rDNA repeats and their susceptibility to RIP mutation**. (A) A multiple alignment of Y1 rDNA repeats found in the genome of *S. nodorum *strain SN15. Each repeat was compared for mutation with the alignment majority consensus (black = match, grey = mismatch, white = gap). The mutation of CpN di-nucleotides is color-coded according to the legend (left). In *S. nodorum *RIP is characterised by the mutation of CpA di-nucleotides (red). Y1 rDNA repeats are grouped according to their genomic location and length. Full length rDNA repeats scattered randomly throughout the genome are prone to RIP whereas short, incomplete copies (defined as <1 kb but generally <300 bp) are not affected. rDNA repeats located in a tandem array at the 3' end of scaffold 5 [NCBI: CH445329] are protected from RIP, excepting a single repeat. (B) The *S. nodorum *tandem rDNA array, also known as the nucleolus organiser region (NOR), and flanking regions. Region 1 contains gene encoding regions, region 2 contains non-rDNA repeats and regions 3 and 4 comprise the tandem rDNA array. RIP mutates repetitive DNA, hence genes in region 1 are not RIP-mutated but repeats in region 2 are RIP-mutated (indicated in red). The tandem rDNA array repeats are protected from RIP (region 4), except for a single repeat at the array terminus (region 3).

An initial survey of the nuclear genome sequence of a West Australian isolate (strain SN15) [19] identified 26 repeat families which comprised 6.2% of assembly. The role and origin of several repeat families could not be inferred by homology. We ascribed this to RIP mutation, after which all copies were unrecognisable. RIPCAL analysis showed that the repetitive DNA of SN15 was subject to RIP-like changes [20]. The rDNA repeat (Y1) exhibits selective susceptibility to RIP mutation (Figure 1). RIP does not affect copies located within the tandem rDNA array (also referred to as the nucleolus organiser region, or NOR, Figure 1: regions 3 & 4) [1,34]. One exception was found in a repeat at the array terminus, which showed evidence of RIP at similar levels to those of non-rDNA array repeats. rDNA repeats were also found scattered throughout the genome. Within the non-rDNA array repeats, short repeats (defined as < 1 kb, however the majority were < 300 bp) did not show evidence of RIP whilst the long repeats (> 1 kb) were RIP-affected [20]. Due to the presence of both RIP-affected and non-RIP-affected copies, the rDNA repeat was perfectly suited to be used as a test case for the validity of bioinformatic predictions of RIP.

The presence and activity of transposons in *S. nodorum *had previously been studied using a transposon trap procedure [35]. Several strains of *S. nodorum *from the United Kingdom (UK) were plated on chlorate [36] to select for mutations in the nitrate reductase (*Nia1*) structural gene. Using the cloned *Nia1 *gene as a probe [37] several insertional mutants were identified. Three insertions were cloned and sequenced [35]. These insertion sequences, named Molly, Pixie and Elsa, represented intact copies of active transposons (Table 2). Southern blots probed with these transposons revealed large variations in copy number, band size and band intensity between strains. When the sequences of the intact copies of these transposons were compared to the genome sequence of the SN15 strain [19], related repetitive regions were identified. However, no active (non-RIP-affected) copies of these transposons were found in the SN15 assembly. The lack of active transposons within SN15 was intriguing and raises the question of the relationship of the repeat families to the active transposons in the UK isolates. This relationship is addressed here.

**Table 2:** Validation of the deRIP technique comparing homology of majority- and deRIP-consensus sequences with non-RIP-affected sequences.

		Blastn homology						Needleman-Wunsch Global Alignment		
		Majority consensus		deRIP consensus		deRIP improvement factor		Majority consensus	deRIP consensus	deRIP improvement to percent identity
Repeat class	Hit Accession	e-value	bitscore	e-value	bitscore			Percent identity		
**(A) Comparisons to active transposon sequences**										

Elsa	AJ277966	1.00E-51	216	1.00E-121	381	1.8 X	69.2%	73.1%	3.9%	
Molly	AJ488502	7.00E-07	66	3.00E-86	329	5.0 X	72.3%	77.5%	5.2%	
Pixie	AJ488503	5.00E-07	66	2.00E-28	137	2.1 X	72.5%	75%	2.5%	

**(B) Comparisons to RIP-protected rDNA array consensus (Figure 1: region 4)**										

Long, non-rDNA array repeats > 1 kb		0	12800	0	17220	1.3 X	89.5%	94.0%	4.5%	
Short, non-rDNA array repeats < 1 kb		3.00E-10	58	1.00E-27	122	2.1 X	46.2% ^a^	45.6% ^a^	-0.6%	
RIP-mutated terminal rDNA array repeat (Figure 1: region 3)		0	8258	--	--	--	85.8%	--	--	

^a ^Needleman-Wunsch global alignment was performed using a sub-region of long rDNA repeats corresponding to the short rDNA repeat consensus										

Blastn hits and pairwise global percent identities to non-RIP-affected sequences were compared between the majority consensus and deRIP consensus versions. (A) The transposons Elsa, Molly and Pixie of *S. nodorum *SN15 were compared to active copies of an alternate strain. In all 3 cases the deRIP sequences match best to the active transposons. This is indicated by the 'deRIP improvement' factor and the differences in percent identities for global alignments. DeRIP improvement is a measure of how much better the deRIP consensus matched the hit compared to the majority consensus. DeRIP improvement > 1 indicates that the repeat family was derived from the hit or a related homolog, but was subsequently mutated by RIP. (B) RIP-protected copies of the *S. nodorum *rDNA repeat are located within a tandem array (Figure 1). RIP-susceptible copies were grouped by size into long (> 1 kB) and short (< 1 kB) categories and compared to the RIP-protected copies. Homology between RIP-protected repeats in rDNA array and long RIP-susceptible non-rDNA array repeats were improved by deRIP. The rDNA array also contains one RIP-affected repeat at its terminus which shows similar levels of homology to the rDNA array as the majority consensus of the long non-rDNA array repeats.

Building upon the RIPCAL procedure, we describe here a new technique to reverse the effects of RIP mutation *in silico*: "deRIP". The deRIP process involves scanning a multiple alignment of a repeat family for RIP-like polymorphism and reverting the alignment consensus to the putative pre-RIP-mutated sequence. The resultant "deRIPped" sequence is a prediction of what a RIP-mutated repeat DNA may have looked like prior to RIP mutation. We have applied the deRIP process to the repetitive DNA of *S. nodorum *SN15 which has increased the number of recognisable repeat families from 65% (17/26) to 92% (23/25).

## Results

### Validating the deRIP process using known non-RIP-affected repeats

The repetitive DNA content of the *S. nodorum *SN15 nuclear genome was previously estimated to contain 26 families comprising 6.2% of the assembly [19]. A repeat family was defined if there were 10 or more copies, of greater than 200 bp and sharing greater than 65% sequence identity. Each family had been analysed by RIPCAL and the extent of RIP measured using the CpA↔TpA dominance statistic [20]. RIP dominance varied from 0.2 to 2.96 (by comparison to the highest G:C content sequence). Blast comparisons predicted the origin of 17 out of the 26 repeat families.

Functional and authentic transposon homologues of the repeat families Molly, Pixie and Elsa had been previously characterized. Elsa was identified as a LTR retrotransposon; Molly and Pixie as Tc-1 Mariner elements [38]. Characterized sequences were derived from UK isolates of *S. nodorum *[35]. The maximum sequence identity between the proteins encoded by the active copies and matches within the SN15 genome assembly was approximately 66% by blastx (Additional file 1).

**Figure 2:**
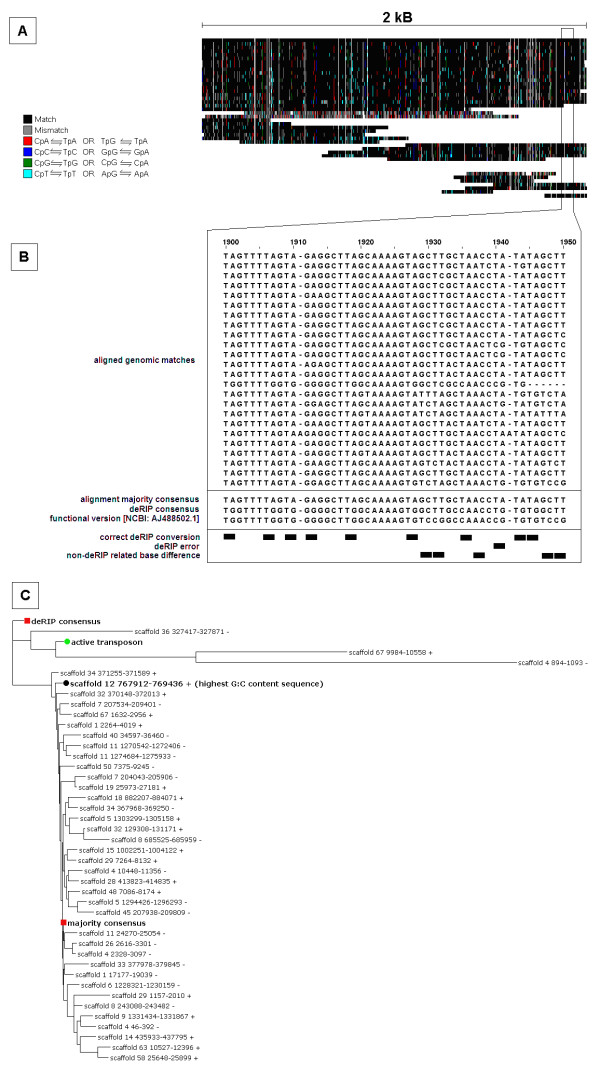
**Application of the deRIP process to the Molly transposon repeat family of *Stagonospora nodorum *SN15**. Molly is one of three *S. nodorum *repeats with known functionally transposable sequence available [NCBI AJ488502.1]. (A) Genomic matches to the Molly repeat family were aligned and compared for RIP-like polymorphism against a model sequence (in this case the majority consensus). RIP mutation of the form CpN ←→ TpN was color-coded as indicated in the legend. (B) The deRIP process was applied to a 51 bp sub-region of the alignment. A 'majority' consensus of the alignment represented the most abundant nucleotide at each alignment position. The deRIP consensus was derived from the majority consensus, however where di-nucleotides were detected exhibiting RIP-like polymorphism (Table 1) they were reverted back to their pre-RIP state. Changes in sequence between majority and deRIP consensus sequences was compared to the sequence of the active transposon. (C) Phylogram showing relationships between all genomic regions, majority consensus, deRIP consensus and active copy of the Molly repeat family. The deRIP consensus resembled the functional transposon more closely than the majority consensus, highest G:C content sequence and the majority of matching genomic regions.

To determine whether the SN15 repeats were derived from the active copies via RIP mutation, the deRIP procedure was applied to the alignment of the Molly, Elsa and Pixie-like sequences. The example shown in Figure 2 illustrates the deRIP process applied to the transposon repeat Molly.

Molly-like repeat sequences of SN15 were aligned and analysed for RIP mutation via RIPCAL [20] (Figure 2A). The alignment includes 18 full length copies and 22 incomplete copies. Mismatches between individual repeats and the majority consensus are colour-coded; vertical red bars represent the CpA/TpG to TpA di-nucleotide substitution previously shown to be the predominant RIP-induced change in *S. nodorum *[20]. The predominance of red changes indicates that the repeat family has been affected by RIP.

The Molly alignment was processed using the new deRIP algorithm. The process is illustrated in Figure 2B in a 51 bp subsection of the alignment from position 1900 to 1950. At position 1900-1901 of the alignment there is a TpA di-nucleotide in 23 out of 24 copies and TpG in one copy. This set of di-nucleotides corresponds to the TpG→ TpA mutation, which is characteristic of RIP (the reverse complement of CpA→ TpA, Table 1). It was assumed that the TpA copies were derived from an ancestral TpG via RIP. Therefore while the majority consensus (alignment consensus by base majority) was TpA at this position, the deRIP process changed this to the most probable pre-RIP sequence - TpG. This process was extended across the length of the repeat alignment, producing a new sequence called the 'deRIP consensus'. This deRIP consensus sequence was compared to the majority consensus as well as the sequence of the active copy of Molly [NCBI: AJ488502.1] (Figure 2B). In this example, deRIP changes were labelled as "correct" where alterations in the deRIP consensus agreed with the sequence of the active copy. Nine such cases occurred in the highlighted section. DeRIP changes were labelled as "errors" where the deRIP changes and the active copy sequence did not agree. There was one deRIP error in the sub-alignment at alignment position 1940-1. Non-deRIP related base differences, common between the majority and deRIP consensus sequences but different in the active copy sequence, occurred five times in the sub-alignment.

All the Molly-like repeats, the active copy, the alignment 'majority' consensus sequence and the new deRIP consensus sequence were compared via RAxML (using the gamma model and maximum-likelihood phylogeny) [39] (Figure 2C). The deRIP-predicted sequence was a closer match to the authentic, active copy than the majority consensus. The relative levels of sequence similarity between the active transposon and the majority and deRIP consensus sequences were also tested via Needleman-Wunsch global alignment [40]. The sequence identity between the active copy and majority consensus was 72.3% whereas the identity between the active copy and the deRIP consensus was 77.5% (Table 2).

The deRIP process was applied to the other transposon repeat families with pre-existing characterized active copy sequences, Pixie and Elsa. Table 2A summarises the results for all three previously identified active *S. nodorum *transposons. In the case of Molly, the majority consensus by blastn had an e-value to the active copy of 7e-07 (bitscore = 66) whereas the deRIP consensus by blastn had ane-value of 3e-86 (bitscore = 329). These results can be summarised as a "deRIP improvement" of 329/66 = 5.0. DeRIP improvement factors were 1.8 and 2.1 and global percent identities to the active transposons were improved 3.9% and 2.5% for Elsa and Pixie respectively (Table 2). The overall improvement in maximum bit scores to active transposons indicates that the deRIP versions were significantly better matches to the functional transposons that were the presumed ancestors of the sequences in the Australian SN15 strain.

The rDNA repeat family Y1 had been previously demonstrated to show differential susceptibility to RIP between its various copies (Figure 1) [20]. rDNA repeats within a tandem rDNA array were not RIP-affected except for one repeat at the array terminus. Non-rDNA array repeats greater than 1 kb (which we call "long") showed evidence of RIP, however non-rDNA array repeats less than 1 kb ("short") did not. After deRIP was applied to the consensus of long, non-rDNA array repeats, the percent identity to the non-RIP-affected rDNA-array consensus was improved by 4.5% - from 89.5% to 94% and the deRIP improvement factor was 1.3 (Table 2). Conversely, the percent identity between the non-rDNA array short repeat consensus and the rDNA array consensus was not improved by deRIP (Table 2). The RIP-affected terminal rDNA repeat and the majority consensus of the RIP-affected long non-rDNA array repeats both had similar levels of homology to the rDNA array (Table 2).

### Determining the role and origin of RIP-degraded repeats in *S. nodorum*

The deRIP process was extended to all repeat families of *S. nodorum *SN15 (Additional files 2, 3, 4). Table 3 summarises the copy number and size of repeat families as estimated previously [19,20]. The extent to which repeat families were affected by RIP is indicated by the RIP dominance scores. RIP dominance [20] was calculated using a variety of comparative models including: sequence of highest G:C content; alignment majority consensus and; consensus sequence predicted by deRIP (Additional file 5). A RIP dominance of greater than 0.6 by comparison to the repeat with highest G:C content was considered a reliable threshold for RIP [20].

**Table 3:** Summary of RIP mutation in the repeat families of S. nodorum strain SN15.

			RIP Dominance Scores			NCBI NR Protein Blastx			GIRI Repbase Tblastx		
**Repeat Family**	**Copy Number**	**Full Length (bp)**	**RIP dominance by highest G:C content **[20]	**RIP dominance by majority consensus**	**RIP dominance by deRIP consensus**	**Hits to deRIP consensus**	**Hits to majority consensus**	**deRIP Improve-ment factor (Maximum value)**	**Hits to deRIP consensus**	**Hits to majority consensus**	**deRIP Improve-ment factor (Maximum value)**

R8	48	9143	2.96	1.95	2.91	126	77	1.96			
R10	59	1241	1.91	0.96	2.07	3	2	2.13	0	3	
X0	76	3862	2.13	0.97	2.05	1	0		3	1	1.34
R9	72	4108	1.88	0.92	1.77	250	25	2.75	124	4	1.28
Molly	40	1862	1.21	0.64	1.73	250	161	3.78	34	15	1.92
X3	213	9364	0.63	0.81	1.62	11	10	2.8			
X35	19	1157	1.5	1.34	1.43	1	0				
X96	14	308	0.87	0.89	1.39						
X48	22	265	1.82	1.16	1.33						
R22	23	678	1.2	0.84	1.28				2	2	1.06
X26	38	4628	1.16	1.08	1.19	57	57	1.38			
Pixie	28	1845	0.77	0.57	1.06	250	190	1.79	17	18	1.25
R37	98	1603	0.49	0.25	0.95	0	55		4	18	1.16
R31	23	3031	0.99	0.83	0.9	16	15	1.44	3	7	1.14
X23	29	685	0.45	0.4	0.9				3	3	0.82
X36	10	512	0.89	0.78	0.87	2	1	1.43			
Elsa	17	5240	0.86	0.78	0.82	250	231	2.06	65	30	1.44
R51	39	833	0.47	0.31	0.8	0	3		0	3	
X11	36	8555	0.83	0.71	0.78	250	250	1.35	250	228	2.18
X12	29	2263	0.67	0.43	0.76	0	1		10	10	1.44
R39	29	2050	0.59	0.28	0.74	173	149	1.54	34	31	1.88
X28	30	1784	0.83	0.59	0.73						
R25	23	3320	0.25	0.6	0.65	4	4	1.19	3	1	0.86
X15	37	6231	0.61	0.46	0.61	250	250	1.45	243	217	1.66
R38	25	358	0.2	0.14	0.5						

RIP dominance, a measure of the strength of RIP mutation, is reported for all 3 different RIPCAL comparison methods: versus the highest G:C content sequence; versus the alignment 'majority' consensus and; versus the deRIP consensus. Measures of how much the predicted deRIP consensus of a repeat family resembles its original version, hit discovery scores and deRIP improvement factors, are also summarised for comparisons against NCBI NR Proteins via blastx and the GIRI Repbase database of repetitive elements via tblastx.

DeRIP consensus-generated RIP dominances correlated with the highest G:C content RIP dominance scores better (correlation coefficient = 0.88) than those of the majority consensus (0.85). This supports the reliability of the deRIP consensus as an accurate prediction of the pre-RIP-mutated progenitor sequence.

Table 3 also lists Blast hits to the NCBI NR and GIRI Repbase. The number of hits of the majority consensus is compared to those of the deRIP consensus sequence. Similarly, the number of hits of either sequence to Repbase is also reported. The deRIP improvement factor used the ratio of highest bit scores of the deRIP and majority sequences to either NR by blastx or Repbase by tblastx respectively. An improvement factor can only be calculated if both consensus sequences have hits above the thresholds (see methods).

In the great majority of cases the number of hits of the deRIP consensus matched or exceeded the number achieved by the majority consensus sequences (Table 3). In two cases, (X35, X0 to NR) the deRIP sequence found a hit where none had been found before (Table 4). In other cases, very substantial increases in hit number were observed (R8, R9 to NR; R9 to Repbase). In a few cases the number of hits was reduced (X12, R37 and R51 by NR; R10, Pixie, R31 and R37 by Repbase).

**Table 4:** Classification of repeat family origin in S. nodorum SN15.

Repeat family	**Predicted origin **[19,20]	Predicted origin after deRIP	comparison type	informative hits	Majority Consensus e-value	deRIP consensus e-value	deRIP improvement factor (maximum)
X26	Sub-telomeric, transposon remnant	Telomere-associated RecQ helicase	blastx vs NR	EAL89306.1 telomere-associated RecQ helicase, putative *Aspergillus fumigatus *Af293	1.00E-07	2.00E-12	1.25

R25	Transposon remnant	Histone H3	blastx vs NR	EDU47581.1 histone H3 *Pyrenophora tritici-repentis *Pt-1C-BFP	0.032	2.00E-04	1.16
			tblastx vs Repbase	TDD4 DNA transposon *Dictyostelium_discoideum*		6.00E-04	

R10	Unknown	Uncharacterized endogenous gene region and DNA transposon	blastx vs NR	EAT76576.1 hypothetical protein SNOG_15997 *Phaeosphaeria nodorum *SN15	2.2	2.00E-13	2.13
			blastx vs NR	EAT81769.1 hypothetical protein SNOG_11270 *Phaeosphaeria nodorum *SN15		5.00E-11	
			blastx vs NR	EAT76052.1 hypothetical protein SNOG_16585 *Phaeosphaeria nodorum *SN15	0.006	2.00E-08	1.40
			tblastx vs Repbase	CR1-3_HM CR1 *Hydra magnipapillata*	9.00E-06		

R31	Unknown	DNA Transposon	blastx vs NR	CAP79587.1 Pc23g00930 *Penicillium chrysogenum *Wisconsin 54-1255	0.013	1.00E-06	1.28
			tblastx vs Repbase	hAT-1_AN hAT DNA transposon *Emericella nidulans*	1.00E-05	1.00E-06	1.07

R39	Unknown	Mariner/Tc1-like DNA transposon	blastx vs NR	EAT91063.1 hypothetical protein SNOG_01414 *Phaeosphaeria nodorum *SN15	2.00E-62	3.00E-73	1.15
			blastx vs NR	EED11513.1 pogo transposable element, putative *Talaromyces stipitatus *ATCC 10500	2.00E-28	1.00E-36	1.20
			tblastx vs Repbase	Mariner-9_AN Mariner/Tc1 *Emericella_nidulans*	8.00E-37	1.00E-25	1.01

R51	Unknown	Mariner/Tc1-like DNA transposon	tblastx vs Repbase	P-29_HM P *Hydra magnipapillata*	1.00E-05		
			tblastx vs Repbase	Mariner-31_HM Mariner/Tc1 *Hydra magnipapillata*	3.00E-05		

X23	Unknown	LTR Retrotransposon	tblastx vs Repbase	ATCOPIA80_I Copia *Arabidopsis thaliana*		1.00E-04	
			tblastx vs Repbase	CR1-3_HM CR1 *Hydra magnipapillata*	9.00E-05	3.00E-04	0.82

X36	Unknown	Retrotransposon	blastx vs NR	EAS29858.1 hypothetical protein CIMG_08604 *Coccidioides immitis *RS	4.9	2.00E-04	1.43
			blastx vs NR	gag-pol polyprotein *Podospora anserina*		4.00E-03	

X3X3R8	X3: Helicase	Endogenous gene cluster containing tandem duplicated Rad5/SNF2-like helicase, Rad6/ubiquitin conjugating enzyme and uncharacterised ORFs	blastx vs NR	EAT83378.1 hypothetical protein SNOG_09186 EAT91019.1 hypothetical protein SNOG_01370 *Phaeosphaeria nodorum *SN15	1.00E-165	0	1.48
			blastx vs NR	EAT90556.1 hypothetical protein SNOG_02344 *Phaeosphaeria nodorum *SN15	6.00E-75	1.00E-122	1.54
			blastx vs NR	EAT83381.1 hypothetical protein SNOG_09189 EAT91023.1 hypothetical protein SNOG_01374 *Phaeosphaeria nodorum *SN15	8.00E-93	1.00E-117	1.51
			blastx vs NR	EAT90553.1 hypothetical protein SNOG_02341 *Phaeosphaeria nodorum *SN15	7.00E-61	1.00E-100	1.51
			blastx vs NR	EAT92620.1 hypothetical protein SNOG_16597 *Phaeosphaeria nodorum *SN15	9.00E-39	2.00E-49	1.43
			blastx vs NR	EAT91018.1 hypothetical protein SNOG_01369 *Phaeosphaeria nodorum *SN15	3.00E-30	1.00E-48	1.44
			blastx vs NR	EAT90555.1 hypothetical protein SNOG_02343 *Phaeosphaeria nodorum *SN15	7.00E-36	5.00E-33	1.31
			blastx vs NR	EAT90554.1 hypothetical protein SNOG_02342 *Phaeosphaeria nodorum *SN15	1.00E-36	2.00E-26	1.34
			blastx vs NR	EAT83379.1 hypothetical protein SNOG_09187 *Phaeosphaeria nodorum *SN15	3.00E-14	1.00E-21	1.28
			blastx vs NR	EAT91020.2 hypothetical protein SNOG_01371 *Phaeosphaeria nodorum *SN15	3.00E-15	2.00E-20	1.19
			blastx vs NR	EAT83294.1 hypothetical protein SNOG_09102 *Phaeosphaeria nodorum *SN15	2.00E-13	4.00E-20	1.25
			blastx vs NR	EAT83377.2 hypothetical protein SNOG_09185 *Phaeosphaeria nodorum *SN15	1.00E-13	8.00E-20	1.23
			blastx vs NR	EAT92618.1 hypothetical protein SNOG_16595 *Phaeosphaeria nodorum *SN15	3.00E-07	4.00E-19	1.60
			blastx vs NR	EAT83380.1 hypothetical protein SNOG_09188 EAT91022.1 hypothetical protein SNOG_01373 EAT92619.1 hypothetical protein SNOG_16596 *Phaeosphaeria nodorum *SN15	4.00E-05	7.00E-15	1.57
			blastx vs NR	EAT91021.1 hypothetical protein SNOG_01372 *Phaeosphaeria nodorum *SN15	2.00E-06	8.00E-14	1.40
			blastx vs NR	EDU40406.1 ubiquitin-conjugating enzyme E2-21 kDa *Pyrenophora tritici-repentis *Pt-1C-BFP	2.00E-10	2.00E-16	1.27
			blastx vs NR	EAW17873.1 ubiquitin conjugating enzyme (*UbcC*), putative *Neosartorya fischeri *NRRL 181	1.00E-07	2.00E-13	1.29
			
	R8: Ubiquitin conjugating enzyme		blastx vs NR	EAT91013.2 hypothetical protein SNOG_01364 *Phaeosphaeria nodorum *SN15	0	0	0.91
			blastx vs NR	EAT92627.2 hypothetical protein SNOG_16589 *Phaeosphaeria nodorum *SN15	0	0	0.91
			blastx vs NR	EAT83373.2 hypothetical protein SNOG_09181 *Phaeosphaeria nodorum *SN15	1.00E-177	0	0.95
			blastx vs NR	EAT90557.2 hypothetical protein SNOG_02345 *Phaeosphaeria nodorum *SN15	2.00E-65	1.00E-106	2.80
			blastx vs NR	EAT90559.2 hypothetical protein SNOG_02347 *Phaeosphaeria nodorum *SN15	1.00E-62	5.00E-91	1.38
			blastx vs NR	EAT91015.1 hypothetical protein SNOG_01366 *Phaeosphaeria nodorum *SN15	3.00E-40	3.00E-62	1.35
			blastx vs NR	EAT85951.1 hypothetical protein SNOG_06120 *Phaeosphaeria nodorum *SN15	2.00E-18	3.00E-24	1.19
			blastx vs NR	EAT91016.1 hypothetical protein SNOG_01367 *Phaeosphaeria nodorum *SN15	1.00E-15	2.00E-23	1.28
			blastx vs NR	EAT83374.2 hypothetical protein SNOG_09182 *Phaeosphaeria nodorum *SN15	5.00E-05	4.00E-08	1.23
			blastx vs NR	EAT91014.2 hypothetical protein SNOG_01365 *Phaeosphaeria nodorum *SN15	1.00E-04	2.00E-04	1.15

After deRIP analysis the predicted origin of 8 repeat families has been altered from that described in Hane & Oliver (2008) [20]. Details of the blast hits which were most informative in re-classifying a repeat family are listed below. E-values are shown for matches to both the majority and deRIP consensus sequences. DeRIP improvement is a measure of how much better the deRIP consensus matched the hit compared to the majority consensus. DeRIP improvement > 1 indicates that the repeat family was derived from the hit or a related homolog, but was subsequently mutated by RIP.

The deRIP improvement factor was greater than one in all cases for NR and in all but two case for Repbase indicating a general increase in the confidence and significance of a hit and hence a functional assignment. The factor ranged up to 3.78 for NR and up to 2.18 for Repbase hits (Table 3). In two cases (R25 and X23) the factor with Repbase was less than 1. This can occur if the hit present in the reference database had been submitted in its non-functional, RIP-affected form.

Blast information was used to determine the origin of several RIP-degraded repeat families of *S. nodorum *SN15. Previously the probable origin of 17 out of 26 repeat families had been identified. However after deRIP had been applied to each repeat family, 23 out of 25 have now been categorised. In six cases (R10, R31, R39, R51, X23 and X36) no previous homology information had been available. Repeat families R31, R39, R51, X23 and X36 were re-classified as transposons after deRIP analysis (Table 4). The repeat family R10, also previously unknown, was identified as corresponding to *S. nodorum *genes SNOG_15997, SNOG_11270 and SNOG_16585 [NCBI: EAT76576.1, EAT81769.1, EAT76052.1].

The previous classification of X15 as a Gypsy class transposon remnant was confirmed after deRIP. The deRIP improvement factors for Gypsy sequences were 1.45 and 1.66 for NR Proteins and Repbase sequences respectively (Table 4). X26, previously predicted to be a transposon remnant, was found after deRIP to contain regions corresponding to a telomere-associated RecQ helicase (Table 4). R25 was previously classified as a putative transposon remnant. After deRIP, some weak homology to DNA transposons was detected versus Repbase but a region of homology to histone H3 proteins was also detected (Table 4). Repeat family R25 was thus re-classified as originating from a (presumably) endogenous gene-encoding region.

**Figure 3:**
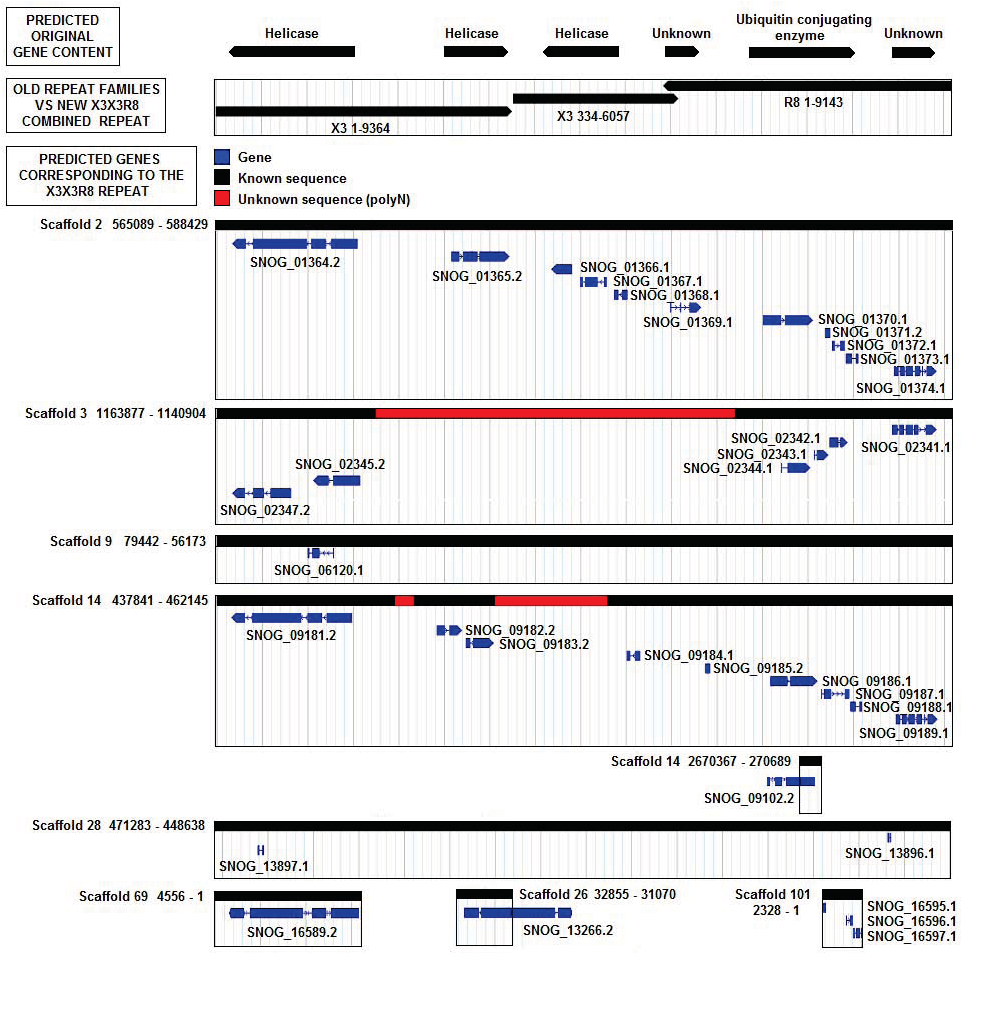
**Nine copies of the repeat X3X3R8 contain predicted gene annotations in the *S. nodorum *genome**. Blast analysis of the deRIP consensus sequence led to the hypothesis that 3 helicase genes, an ubiquitin conjugating enzyme and 2 unknown genes originally occupied this region. The effects of RIP mutation have led to the disruption of open-reading frames in several of these genes resulting in multiple, short-length gene predictions which are highly likely to be pseudogenes.

R8 and X3 were previously predicted to contain the remnants of an ubiquitin conjugating enzyme and helicase genes respectively [19,20]. DeRIP analysis was used to predict the ancestral sequence and identified matches to nine copies of a cluster of endogenous *S. nodorum *genes (Additional file 3). Analysis of repeats X3 and R8 indicated that several copies of these repeat families were physically adjacent (Additional file 3). Studying the location of X3 and R8 repeats revealed that these repeats were frequently arranged in a distinctive pattern roughly corresponding to two tandem X3 repeats followed by a reversed R8 repeat (Figure 3, Figure S# 4). These two repeat classes were combined and renamed X3X3R8.

In addition to the cluster of endogenous genes, the X3X3R8 deRIP consensus also hit known ubiquitin conjugating enzymes with greater homology than the majority consensus (Table 4). Homology relationships to DNA excision/repair helicase regions were inferred from hits to the endogenous *S. nodorum *genes residing within X3X3R8 (Additional file 2).

**Figure 4:**
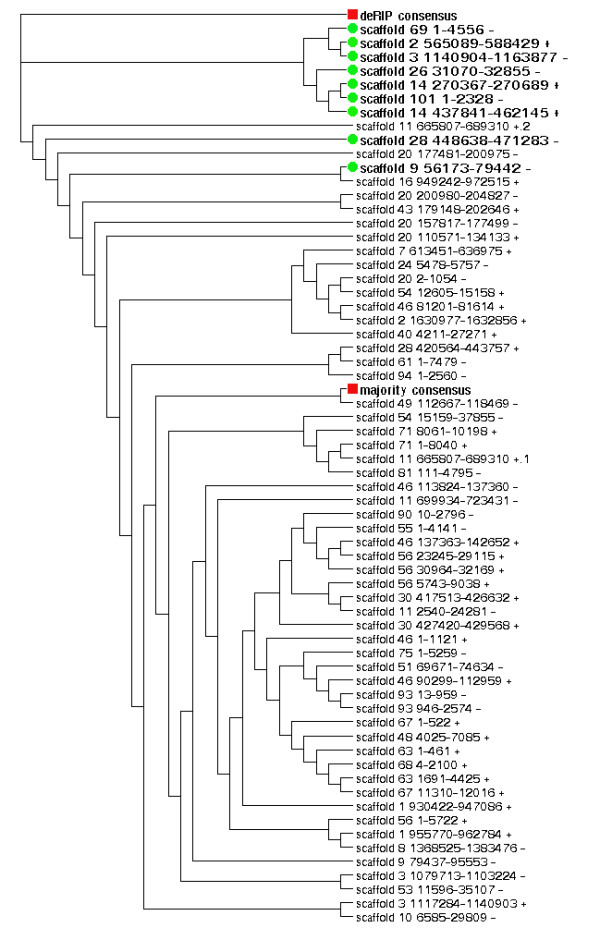
**Comparison of the repeats of the X3X3R8 repeat family with respect to predicted gene content**. The deRIP consensus (red square, top) is a prediction of the original repeat sequence prior to RIP-degradation. Nine X3X3R8 repeats contained predicted gene annotations (green circles, refer to Figure 3). All gene-annotation containing repeats were more closely related to the deRIP consensus than to the majority consensus (red square, middle).

Since the deRIP consensus is a prediction of the sequence prior to RIP-mutation it can be presumed that the X3X3R8 repeats with functional genes are closely related to the deRIP consensus. The majority of repeats with predicted gene annotations were found to be highly similar to the deRIP consensus (Figure 4, green circles). Current evidence supports the functionality of some or all of the genes contained in six out of these nine X3X3R8 repeats.

## Discussion

The deRIP algorithm was designed to reverse the affect of RIP upon repeat families in-silico and thereby help determine the evolutionary history of repeated elements. The process automates the selective alteration of bases within a consensus according to a set of rules that can be determined by considering the RIP machinery operating in the organism concerned. Despite its validation with known non-RIP-affected sequences, deRIP has some limitations and will not necessarily perfectly predict the ancestral sequence. DeRIP can only choose within options provided by the aligned set of repeats. In the example given in Figure 2 the TpA sequence at position 1900-1901 was converted to TpG. To do this, at least one of the copies of the repeat must have the presumably ancestral di-nucleotide TpG at this site. If all extant copies had been mutated, the reversion would have no support. The deRIP process is therefore critically dependent on the degree of RIP within a repeat. The success of deRIP is also dependent upon the accuracy of the alignment. A noteworthy aside is that default alignment parameters often fail to align fungal repeats correctly due to complex internal repeat structures. Finally, the diagnostic metrics of deRIP success, deRIP improvement and hit discovery, are only possible to calculate if appropriate matching sequences exist in the queried databases. If a repeat sequence is truly novel, its "homology" cannot be improved until homologs are found.

RIPCAL uses a model sequence to compare to aligned repeats for RIP-like polymorphism. Selecting the repeat with the highest total count of G and C nucleotides assumes that high G:C content is representative of the least RIP-affected repeat. The majority consensus model on the other hand could be representative of the least or most RIP-affected repeat depending on the level of RIP mutation within the repeat family. Previously, we had selected the sequence with the highest G:C content as the RIPCAL model [20]. While in most cases this rationale is sound, the G:C model has several shortcomings. If a sequence with the highest total G:C content does not span the full length of its alignment, RIP data from the un-covered regions would be lost. Alternatively, a repeat longer than the least RIP-affected repeat (e.g. resulting from a large sequence insertion into a RIP-affected repeat) may have higher total G:C content merely due to its greater length. The G:C model is also sensitive to variations in G:C content not related to RIP. Furthermore, RIP occurs between multiple combinations of repeats over time. The G:C model sequence therefore comprises of an amalgam of pre-RIP and post-RIP di-nucleotides relative to the alignment as a whole.

RIP mutations have directionality (Table 1), so the combination of pre- and post-RIP sites makes it necessary to consider RIP mutation both towards and away from the G:C model sequence. In contrast, a deRIP consensus model, being a prediction of the pre-RIP-mutated sequence, has the advantage of polarity. As such, deRIP mutation calculations can be restricted to one direction: proceeding from the deRIP consensus to the RIP-affected repeat.

The prior isolation of active copies of three transposons, Molly Pixie and Elsa, as well as the differential effect of RIP on the rDNA repeats, allowed a thorough test of the power of deRIP to reconstruct the ancestral sequence. In all four examples the predicted deRIP consensus of the RIP-affected sequences was the best match to the active copy indicating that deRIP was able to accurately revert the RIP-degraded repeats close to their original states. These analyses helped define the concepts of hit discovery number and deRIP improvements as applied more broadly in Table 3.

The deRIP process serves to highlight the effectiveness of RIP as a transposon-silencing mechanism. In most observed cases, the resemblance between RIP-degraded repeats and their non-RIP-affected, functional counterparts is minimal (Additional file 1). In the case of the Molly, Elsa and Pixie transposons, functional sequences of transposon proteins were available for comparison. No viable open-reading frames could be found in any of their respective genomic matches in *S. nodorum *SN15 (Additional file 1). Some repeat families could not even be classified by homology prior to deRIP (Table 4). The deRIP process is therefore an essential tool which facilitates the identification and understanding of the role and origin of fungal repetitive DNA. The effectiveness of deRIP was such that functional assignments were improved quantitatively or qualitatively in nearly all cases. This was most clearly the case when repeat families were most clearly affected by RIP (Table 3, Table 4).

Conversely when the repeat family was not RIP-affected, the deRIP process was not able to improve the homology assignment. An example is the transposon repeat family R37 which had low RIP dominance (by majority consensus) of 0.25, indicating that R37 is not greatly affected by RIP mutation.

The R10 repeat contained regions corresponding to three *S. nodorum *genes (SNOG_15997, SNOG_11270 and SNOG_16585 [NCBI: EAT76576.1, EAT81769.1, EAT76052.1]) (Table 4) which are located in separate regions of the genome assembly. The sub-telomeric repeat X26, which contained telomere-associated RecQ helicase sequence and was subject to relatively high levels of RIP mutation (Table 3). RecQ helicase plays a critical role in genome maintenance and is essential for DNA replication in eukaryotes [41]. Twenty six putative functional copies (i.e annotated gene models) of RecQ are present within the *S. nodorum *genome (Additional file 6). It is currently unclear by what mechanism function is preserved in certain copies of this highly repeated gene family, but not in others.

The repeat family X3X3R8, which replaced the previously defined repeat families X3 and R8, matched to a cluster of endogenous *S. nodorum *SN15 genes (Table 4) - some of these coding for a DNA repair helicase and ubiquitin conjugating enzyme (Figure 3). The helicase and ubiquitin conjugating enzyme genes within X3X3R8 were homologous to *Saccharomyces cerevisiae *proteins Rad5 [SGD: YLR032W] and Rad6 [SGD: YGL058W] respectively (Additional file 2). These proteins are involved in the post-replication repair of UV-damaged DNA, the epigenetic silencing of telomeres and sporulation in yeast [42-44].

The absence of active copies of the Elsa, Molly and Pixie families in the Australian SN15 isolate contrasts with the situation in the UK. Rawson screened several isolates for active transposons and used a trapping process to isolate active copies [35]. Using these transposons as probes showed great variation in copy number and band intensity amongst a collection of UK isolates. We have only looked at one Australian isolate, but it appears to be devoid of active transposons (Additional file 1). Consideration of the properties of RIP, the need for sexual reproduction by the fungus in Mediterranean climates and the biogeography of *S. nodorum*, could explain the absence of transposons in the Australian isolate. Repeated elements over a threshold size and above a threshold identity would be subject to RIP. This would inactivate all copies of a transposon during a meiotic event that appears to be necessary for survival over the hot summer [30]. It would not be conceivable for an active copy to be reconstituted in an asexual population derived from such an event. The survival of a transposon in a population of a sexually reproducing fungus would require mating with an isolate with an active (and presumably single) copy of the transposon. The invasion of *S. nodorum *into Australia most likely occurred via the propagation of a small founder population consistent with the reduced polymorphism of populations found here [33]. We speculate that no active transposons have survived within any Australian isolate capable of RIP. Screening of a larger population of Australian and Eurasian isolates to determine differences in frequency and distribution of active copies between the founder and derived populations would be required to confirm this.

## Conclusions

In summary, we present a facile and rapid method to assist the annotation of repetitive elements of ascomycete genomes. The deRIP process can predict ancestral functional sequence from degraded repeat elements. Analysis of the repeat families of the fungal phytopathogen *Stagonospora nodorum *(strain SN15) using deRIP-converted sequences increased the number of recognisable repeat families from 65% (17/26) to 92% (23/25). This has enabled the characterization of many repeat families and has advanced our progress towards the goal of understanding and accounting for the evolutionary history of all regions of a genome.

## Methods

### Analysis of RIP-mutation of *S. nodorum *repetitive DNA

The 26 distinct repeat families of *S. nodorum *SN15 [19] were analysed for RIP mutation using RIPCAL [20]. RIPCAL requires an appropriate model sequence, which is a template to which all other aligned sequences of the repeat family are compared for RIP-like polymorphism. Previously we used the sequence of highest total G:C content as the model sequence. As RIP irreversibly converts G:C nucleotide pairs to A:T, it was assumed that the sequence with the highest G:C content was the least RIP-affected repeat in the family. In this study, we have performed RIPCAL analyses using 3 different models: highest G:C content, alignment majority consensus and predicted sequence of the repeat family prior to RIP-mutation.

### Predicting the original repeat sequence prior to RIP-degradation

The deRIP process predicts the sequence of the pre-RIP-mutated version of the repeat alignment. Firstly, the majority consensus was generated by counting the nucleotide frequency at each position of the repeat alignment. The majority consensus sequence was determined by the highest frequency nucleotide. Secondly, at each position of the multiple alignment, counts of di-nucleotides exhibiting RIP-like polymorphism (CpN → TpN) were calculated (Table 1). A RIP mutation with the highest corresponding di-nucleotide count was presumed to be dominant and therefore the majority consensus was converted to the appropriate pre-RIP di-nucleotide sequence. This predicted sequence is henceforth referred to as the 'deRIP consensus'.

### Validating the deRIP technique

The sequences of the active copies (which are presumably non-RIP-degraded) of the *S. nodorum *transposon repeats Molly, Pixie and Elsa [NCBI; AJ277966, AJ488502, AJ488503] [35] were compared to their respective majority and deRIP consensus sequences from strain SN15 via blastn [45]. Majority and deRIP consensus sequences of these repeat families were also globally aligned against their respective active copy via needle [40]. The SN15 rDNA repeat (Y1) was previously shown to be differentially susceptible to RIP [20]. The majority consensus of the non-RIP-affected copies of Y1 were compared to the majority and deRIP consensus sequences of the RIP-susceptible copies as above. The relative difference in alignment bit scores between majority and deRIP consensus sequences with their respective active copies was used to measure the degree of 'improvement' of the deRIP consensus over the majority consensus:

$$\frac{\text{Bit score of best HSP }(\text{deRIP consensus})}{\text{Bit score of best HSP }(\text{majority consensus})}$$

A 'deRIP improvement factor' greater than 1 indicated that the deRIP process had modified the RIP-affected sequence to resemble the sequence of the active copy.

### Predicting the origin of RIP-degraded repeats

Majority and deRIP consensus sequences were compared to the NCBI NR protein database via blastx [45] and to the GIRI Repbase database of repetitive DNA [46] via tblastx. The results of these comparisons were used to infer repeat family origin and function. In this analysis, NCBI and Repbase sequences were both assumed to represent active transposons. Stronger deRIP matches to either database indicated that the deRIP algorithm was able to convert a RIP-inactivated sequence back into that of an active transposon. A maximum e-value threshold of 10 was imposed on hits against both the majority or deRIP consensus, with one of these also required to be less than 1e-3. DeRIP improvement factors were calculated for each hit as above. However for the purpose of summarising this data in Table 3, the maximum value was reported for each respective repeat family. 'Hit discovery scores' are the number of hits that the deRIP or majority consensus sequences have to the NR or GIRI databases. The scores illustrate the extent to which the deRIP process was able to discover new homology relationships that were previously lost due to RIP.

## Authors' contributions

JKH designed the deRIP algorithm and performed the bioinformatics analyses. JKH and RPO wrote the manuscript. All authors read and approved the final manuscript.

## Supplementary Material

Additional file 1 **Test for viable copies of the transposons Molly, Pixie and Elsa in the *S. nodorum *SN15 genome**. Click here for file

Additional file 2 **Summary of deRIP improvement and hit discovery scores**. Contains summaries of the RIPCAL analyses for highest G:C content, majority consensus and deRIP consensus comparisons. Also contains details of majority and deRIP consensus hits by blastx to the NCBI NR Protein database and by tblastx to the GIRI Repbase database. Click here for file

Additional file 3 **Merging of the previously identified repeat families X3 and R8 to form the new repeat family X3X3R8**. Click here for file

Additional file 4 **Merging of the previously identified repeat families X3 and R8 to form the new repeat family X3X3R8**. Supplementary Figure, PNG format. The previously predicted X3 and R8 repeat families (HANE and OLIVER 2008) were found to correspond to genomic regions in a distinctive repeated pattern which spanned 26 kB. This region was classified as a new repeat family, X3X3R8, which supersedes the old repeat families R8 and X3. The MUMMER dot-plot above illustrates how the nucleotide majority consensus sequences of R8 and X3 relate to X3X3R8. The first third of the X3X3R8 majority consensus corresponds to a full length copy of X3. The second third of X3X3R8 is comprised of a second, incomplete copy of X3 which in matching regions is 10-20% divergent from the X3 consensus. The final third corresponds to a complete copy of the R8 repeat, in the reverse orientation with respect to its previously defined sequence. Click here for file

Additional file 5 **deRIP RIPCAL analysis of the repetitive DNA of *S. nodorum *SN15**. RIPCAL outputs for highest G:C, consensus and deRIP models versus S. nodorum repeat families, tab-delimited txt and gif formats. Click here for file

Additional file 6 **List of predicted functional RecQ helicases in the *S. nodorum *genome**. Click here for file
