# Supplementary figures and images for: In silico reversal of repeat-induced point mutation (RIP) identifies the origins of repeat families and uncovers obscured duplicated genes
Source: BMC Genomics. 2010 Nov 24;11:655. doi: 10.1186/1471-2164-11-655 (PMC3017866; doi:10.1186/1471-2164-11-655)

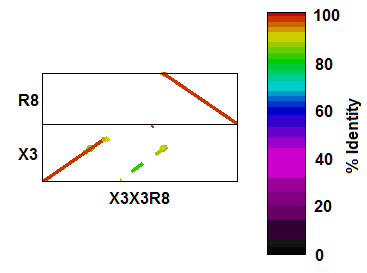

Supplement: Additional file 4 — Merging of the previously identified repeat families X3 and R8 to form the new repeat family X3X3R8. Supplementary Figure, PNG format. The previously predicted X3 and R8 repeat families (HANE and OLIVER 2008) were found to correspond to genomic regions in a distinctive repeated pattern which spanned 26 kB. This region was classified as a new repeat family, X3X3R8, which supersedes the old repeat families R8 and X3. The MUMMER dot-plot above illustrates how the nucleotide majority consensus sequences of R8 and X3 relate to X3X3R8. The first third of the X3X3R8 majority consensus corresponds to a full length copy of X3. The second third of X3X3R8 is comprised of a second, incomplete copy of X3 which in matching regions is 10-20% divergent from the X3 consensus. The final third corresponds to a complete copy of the R8 repeat, in the reverse orientation with respect to its previously defined sequence. [file 1471-2164-11-655-S4.PNG]

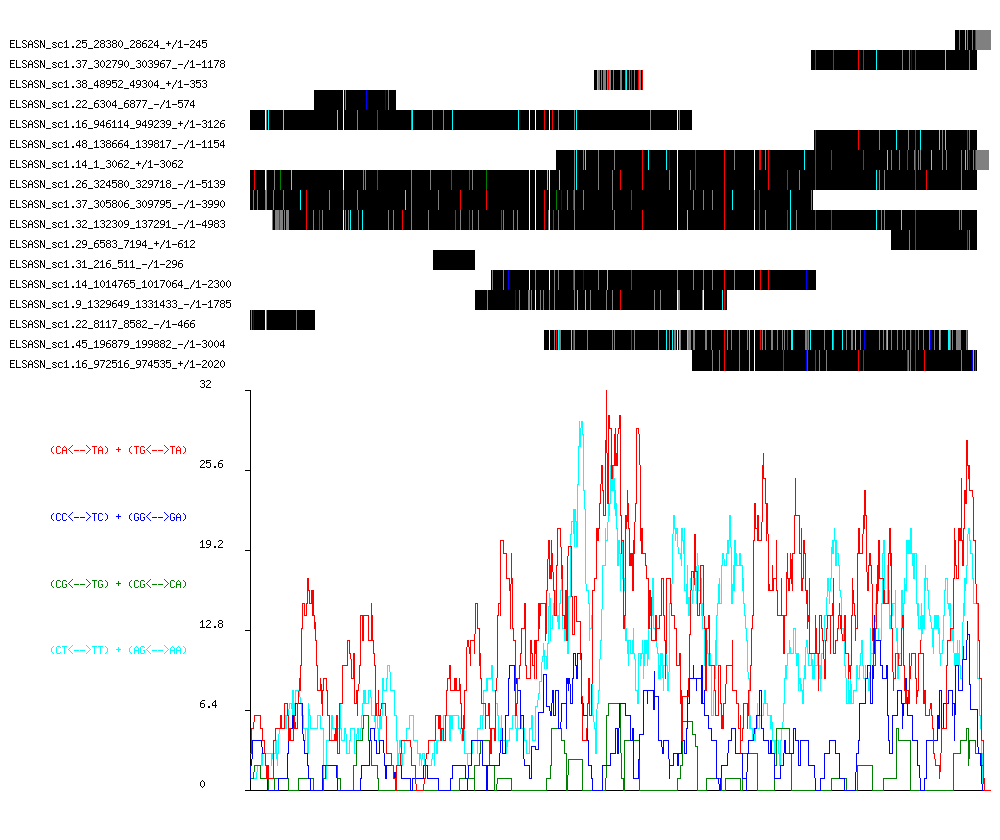

Supplement: Additional file 6 — List of predicted functional RecQ helicases in the S. nodorum genome. [file 1471-2164-11-655-S6.ZIP › consensus/elsa_aln_2009_8_19_15_26_04_input.gif]

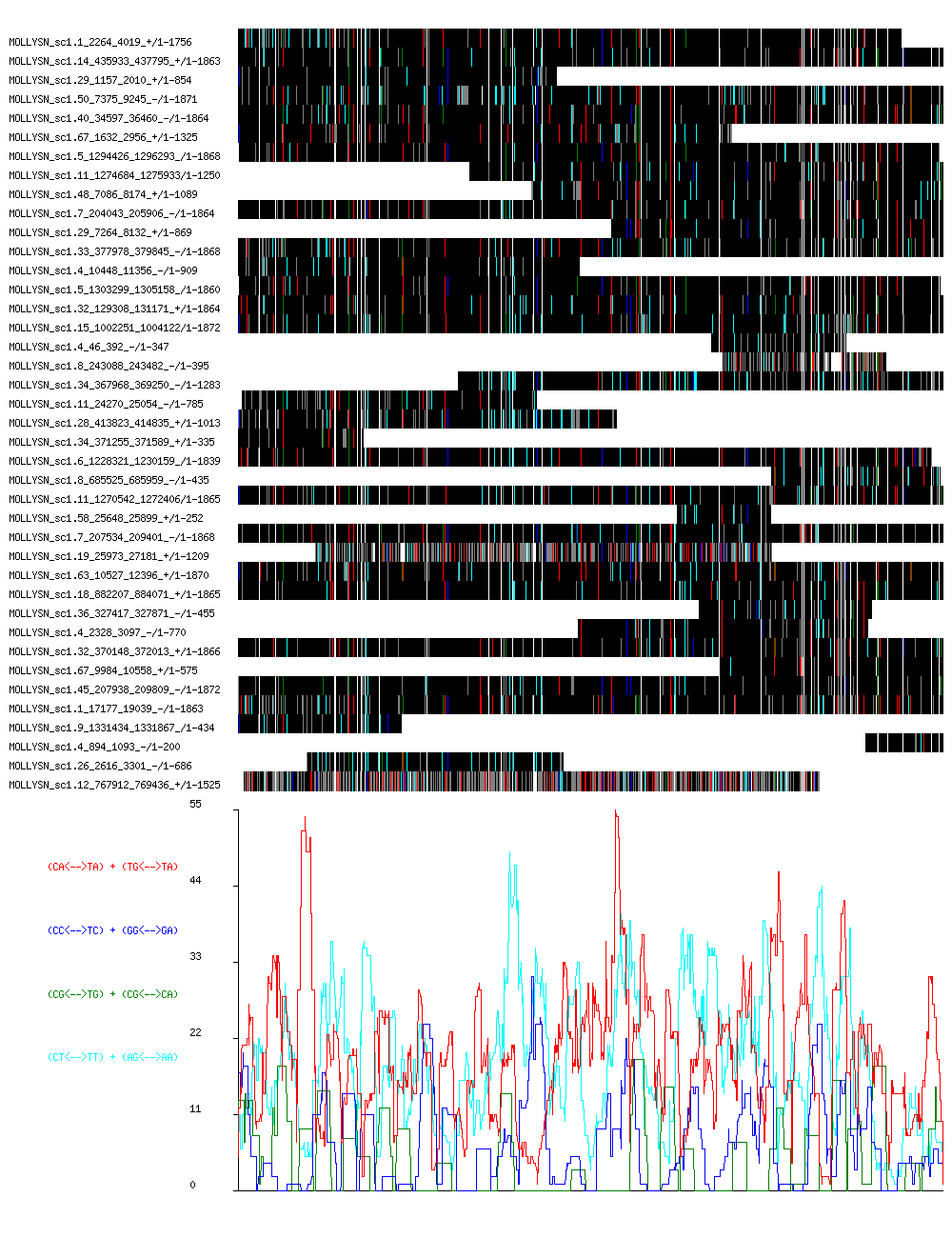

Supplement: Additional file 6 — List of predicted functional RecQ helicases in the S. nodorum genome. [file 1471-2164-11-655-S6.ZIP › consensus/molly_aln_2009_8_19_15_26_18_input.gif]

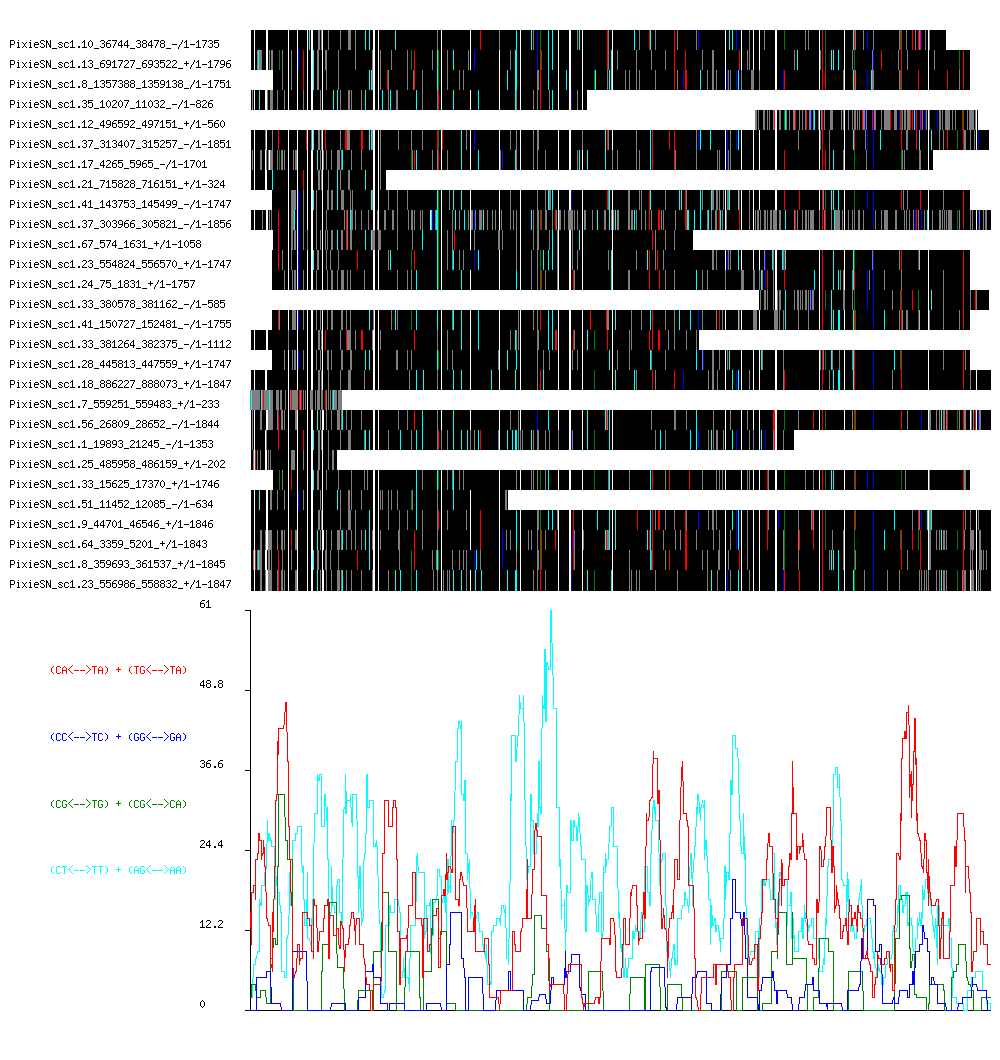

Supplement: Additional file 6 — List of predicted functional RecQ helicases in the S. nodorum genome. [file 1471-2164-11-655-S6.ZIP › consensus/pixie_aln_2009_8_19_15_26_28_input.gif]

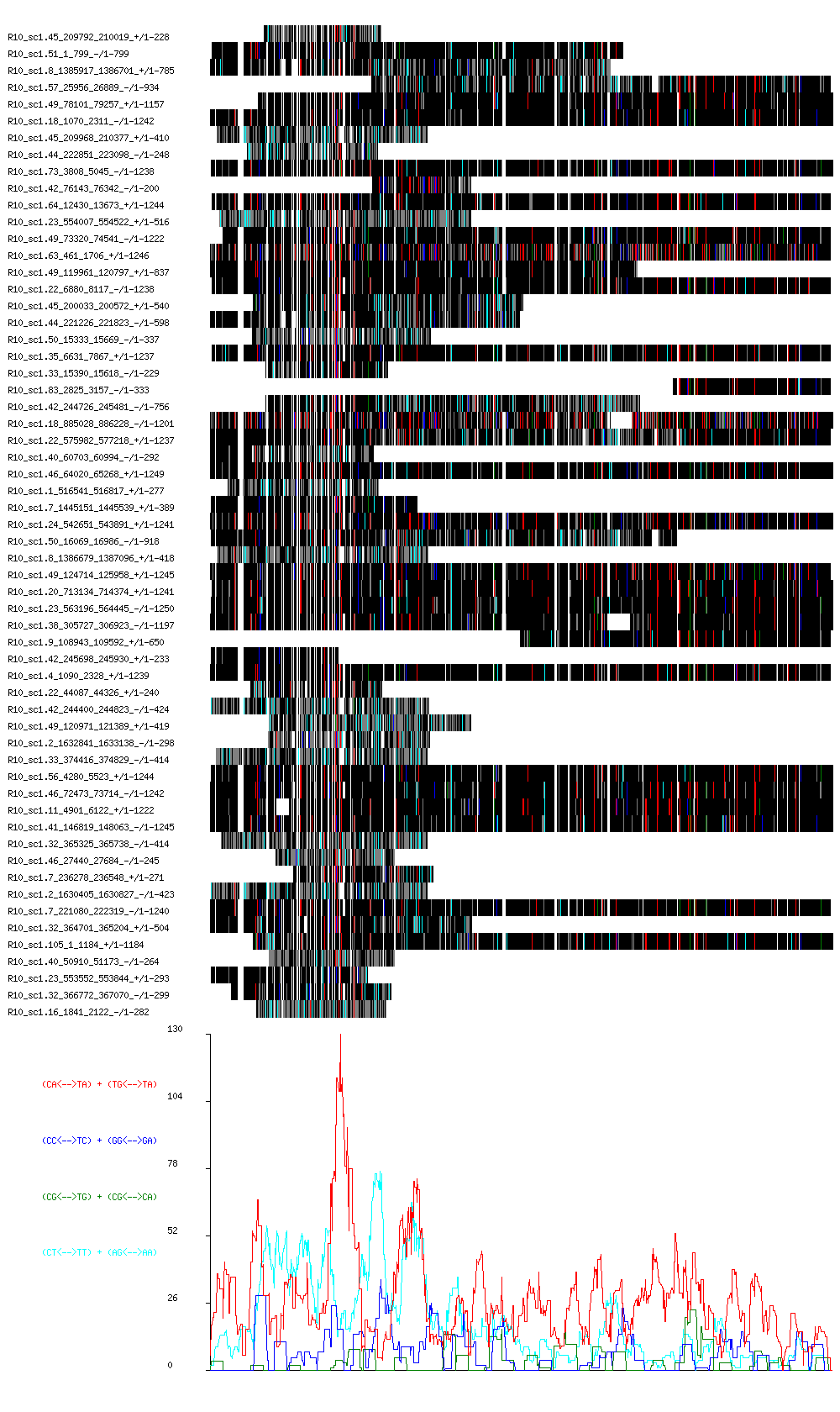

Supplement: Additional file 6 — List of predicted functional RecQ helicases in the S. nodorum genome. [file 1471-2164-11-655-S6.ZIP › consensus/r10_aln_2009_8_19_15_26_35_input.gif]

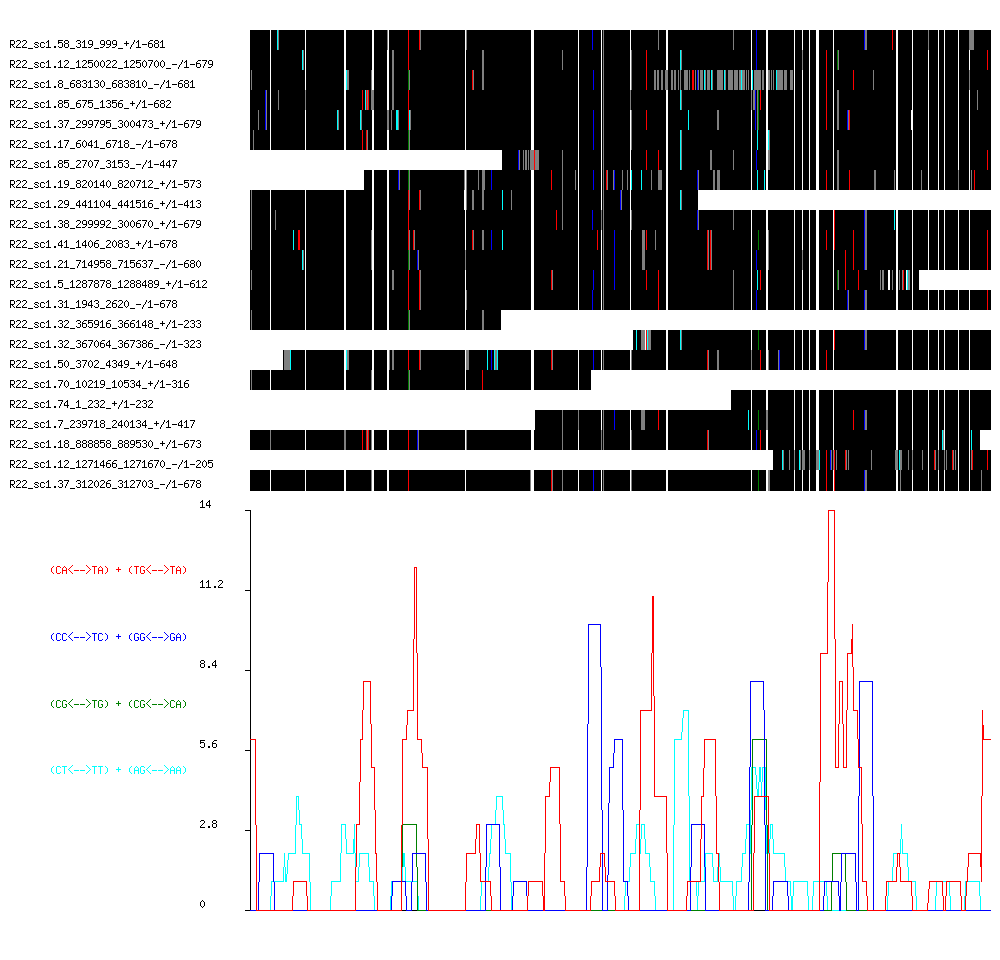

Supplement: Additional file 6 — List of predicted functional RecQ helicases in the S. nodorum genome. [file 1471-2164-11-655-S6.ZIP › consensus/r22_aln_2009_8_19_15_26_45_input.gif]

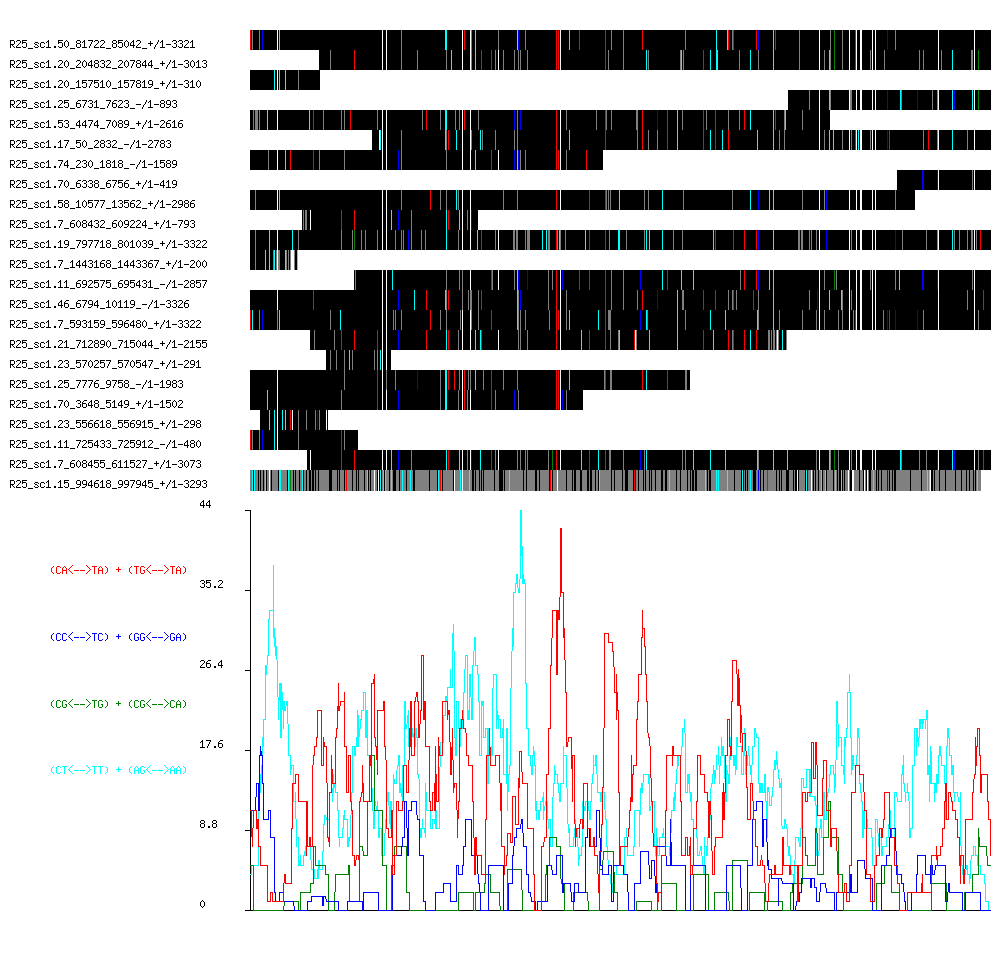

Supplement: Additional file 6 — List of predicted functional RecQ helicases in the S. nodorum genome. [file 1471-2164-11-655-S6.ZIP › consensus/r25_aln_2009_8_19_15_26_47_input.gif]

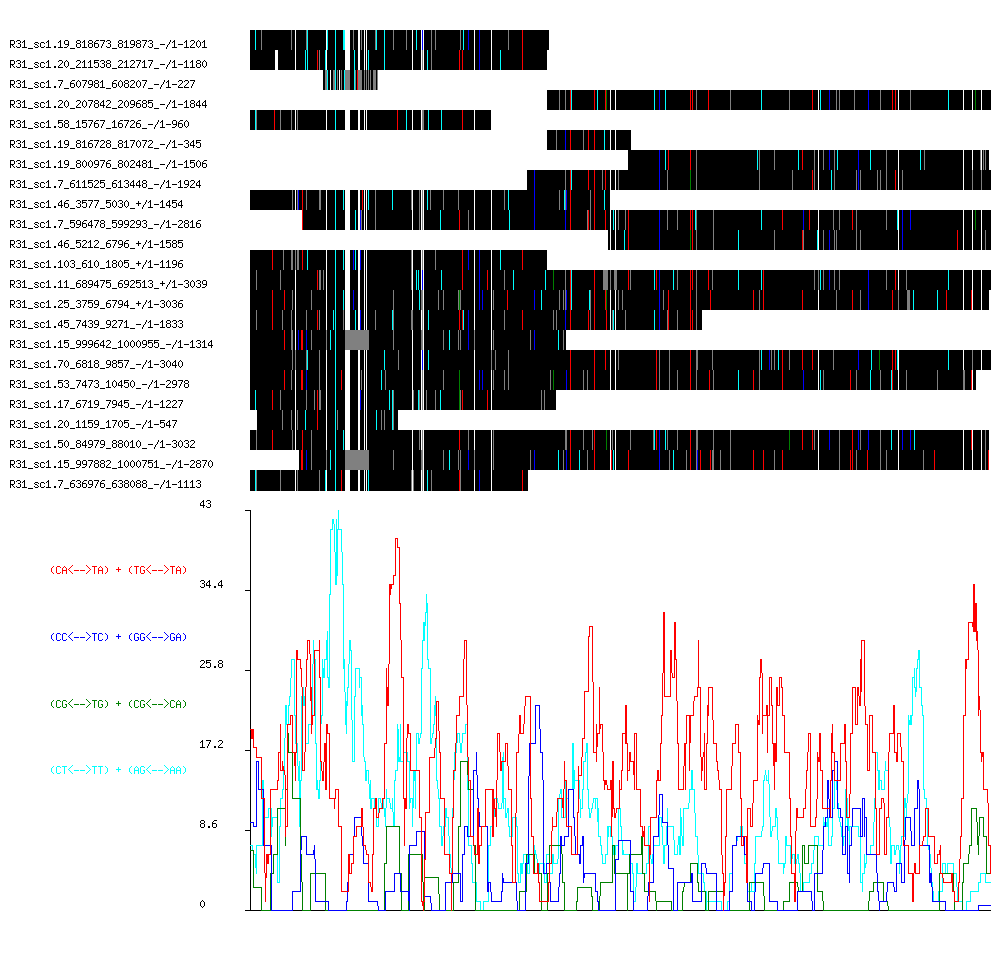

Supplement: Additional file 6 — List of predicted functional RecQ helicases in the S. nodorum genome. [file 1471-2164-11-655-S6.ZIP › consensus/r31_aln_2009_8_19_15_26_58_input.gif]

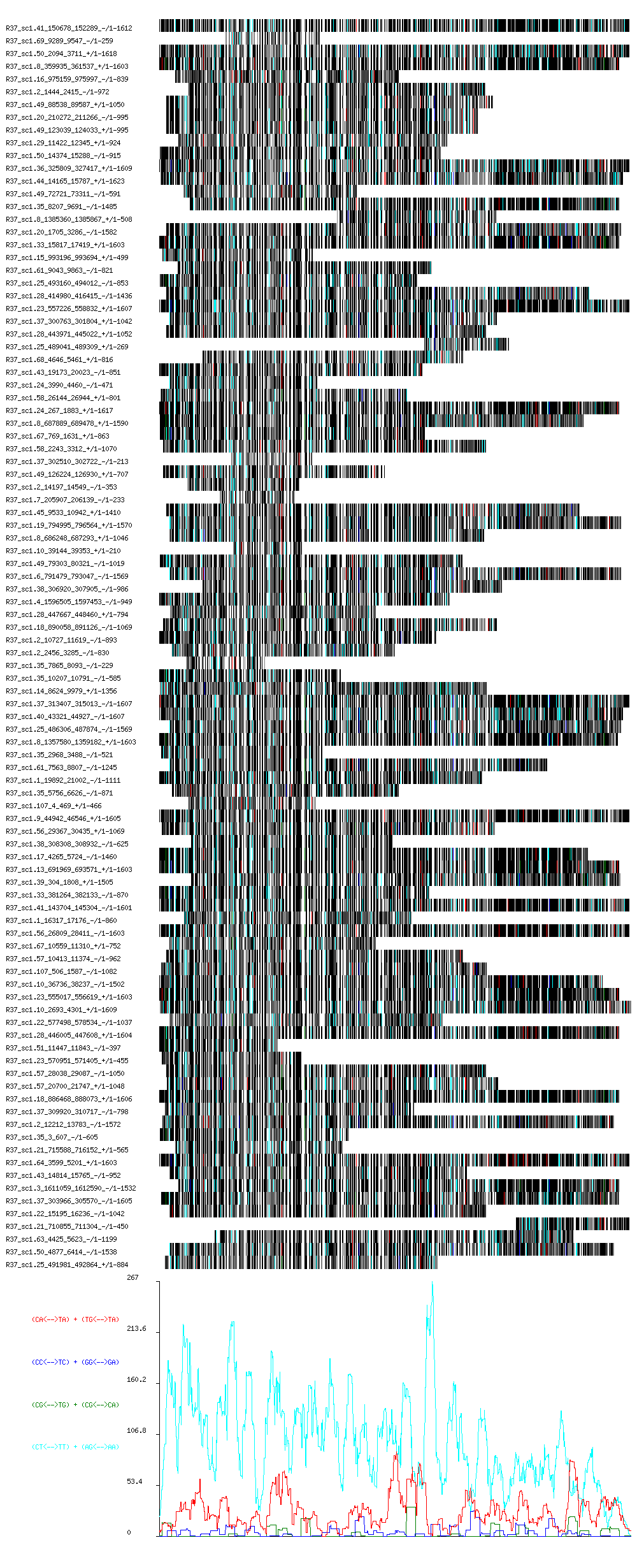

Supplement: Additional file 6 — List of predicted functional RecQ helicases in the S. nodorum genome. [file 1471-2164-11-655-S6.ZIP › consensus/r37_aln_2009_8_19_15_27_08_input.gif]

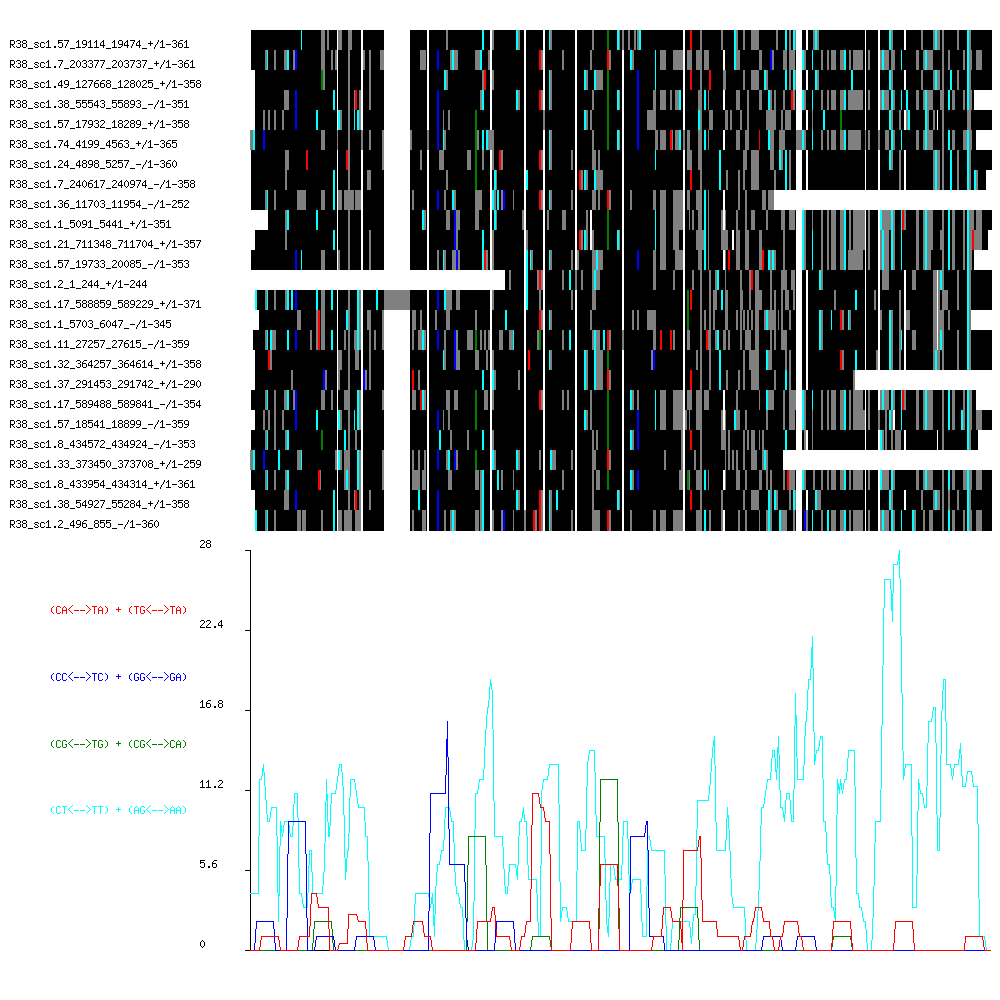

Supplement: Additional file 6 — List of predicted functional RecQ helicases in the S. nodorum genome. [file 1471-2164-11-655-S6.ZIP › consensus/r38_aln_2009_8_19_15_27_35_input.gif]

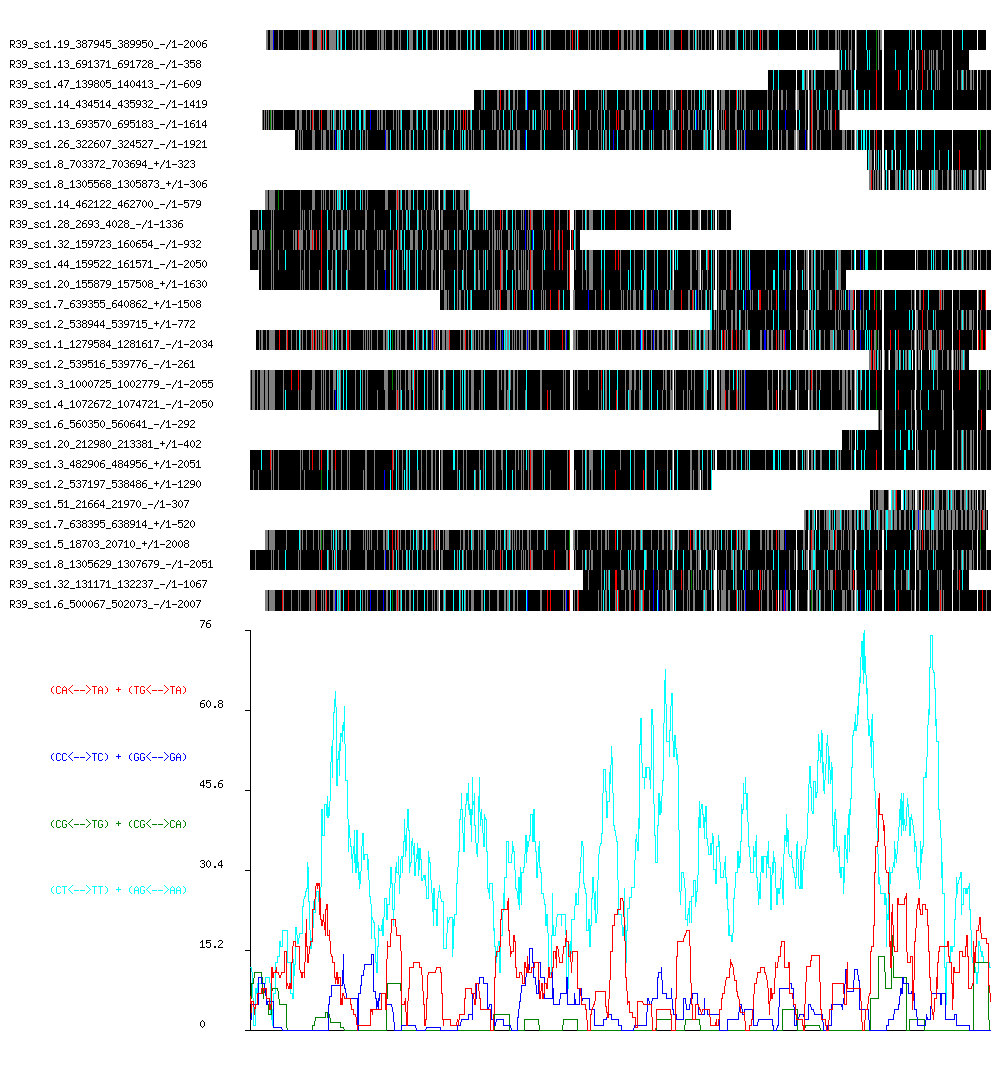

Supplement: Additional file 6 — List of predicted functional RecQ helicases in the S. nodorum genome. [file 1471-2164-11-655-S6.ZIP › consensus/r39_aln_2009_8_19_15_27_37_input.gif]

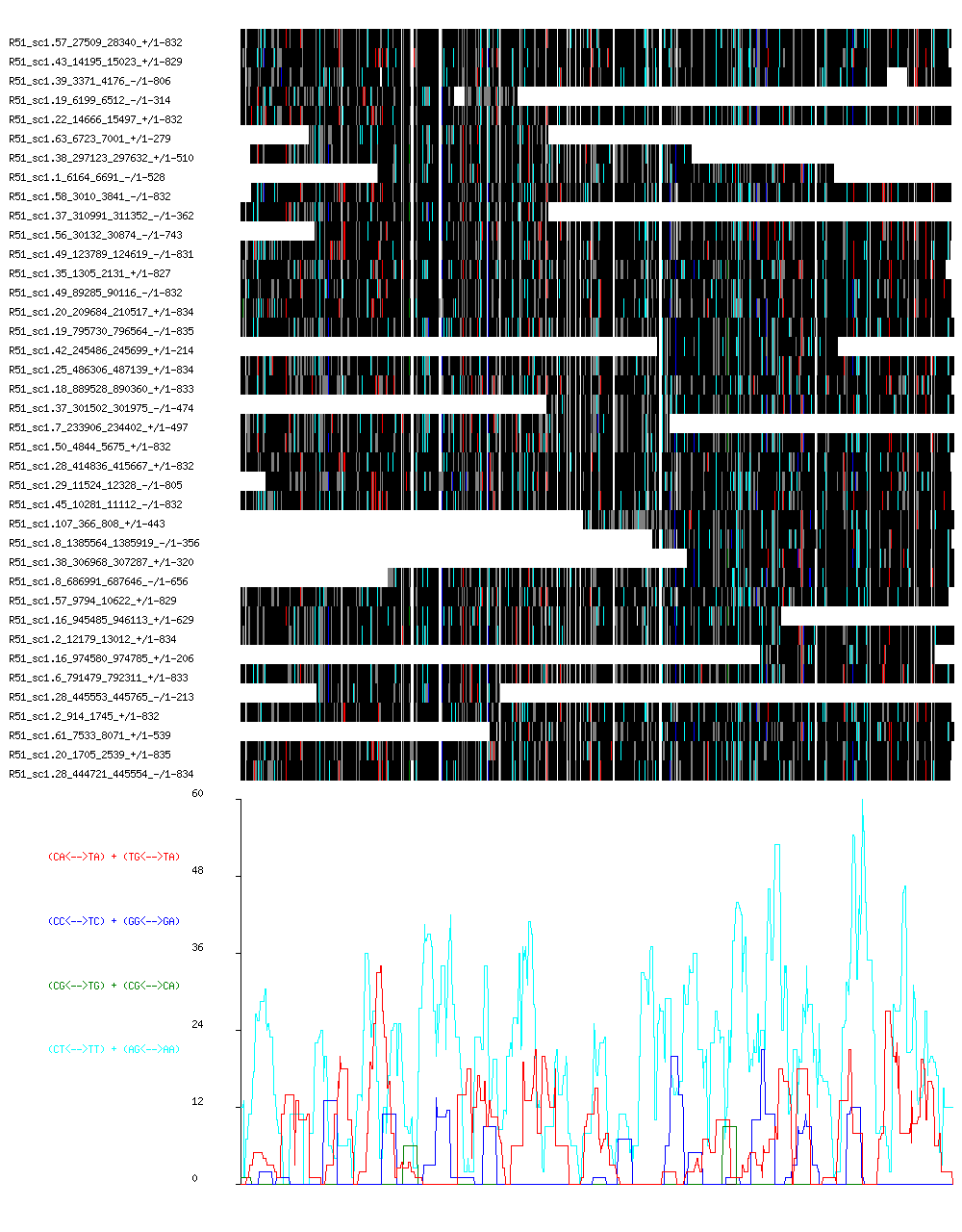

Supplement: Additional file 6 — List of predicted functional RecQ helicases in the S. nodorum genome. [file 1471-2164-11-655-S6.ZIP › consensus/r51_aln_2009_8_19_15_27_45_input.gif]

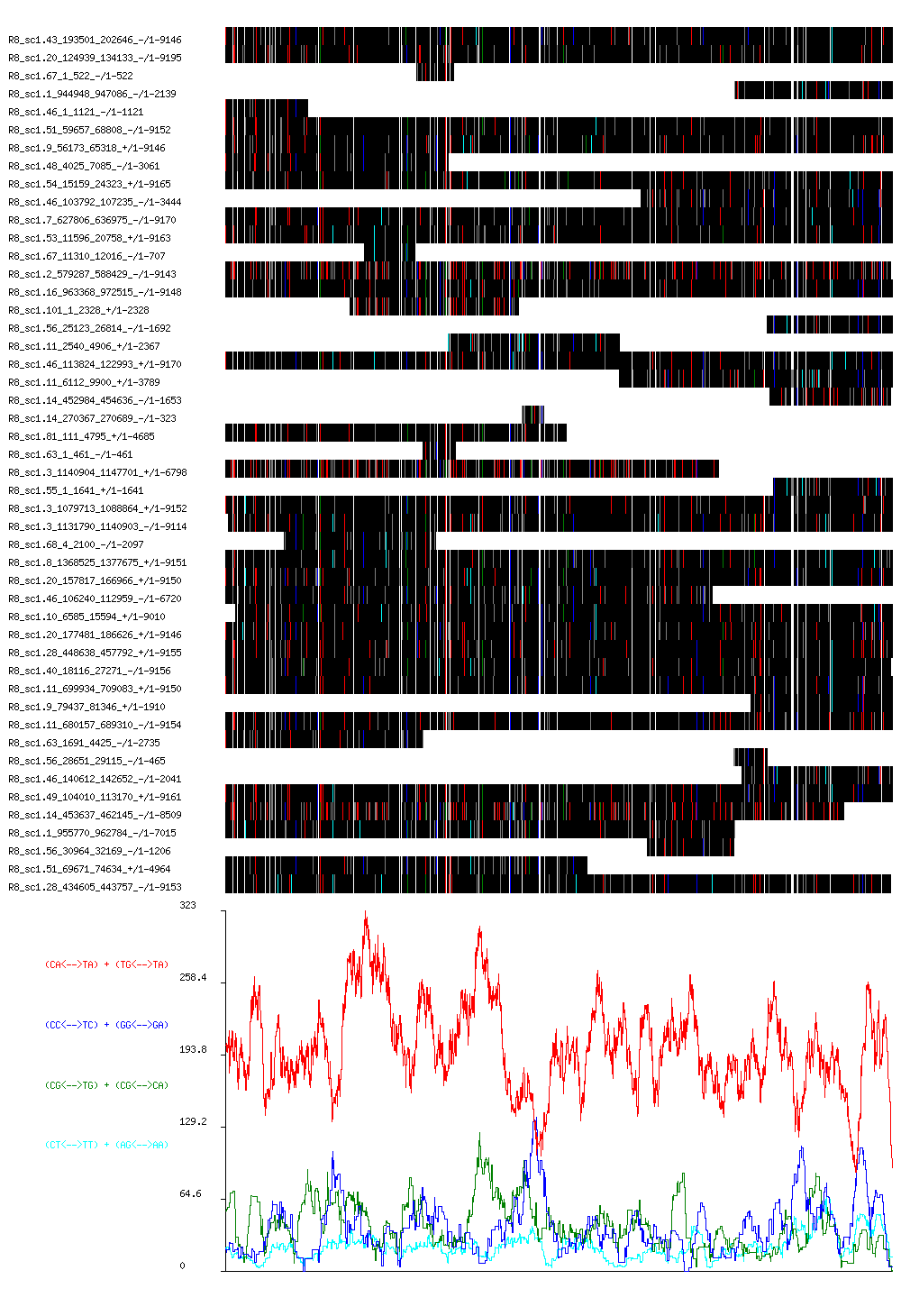

Supplement: Additional file 6 — List of predicted functional RecQ helicases in the S. nodorum genome. [file 1471-2164-11-655-S6.ZIP › consensus/r8_aln_2009_8_19_15_27_49_input.gif]

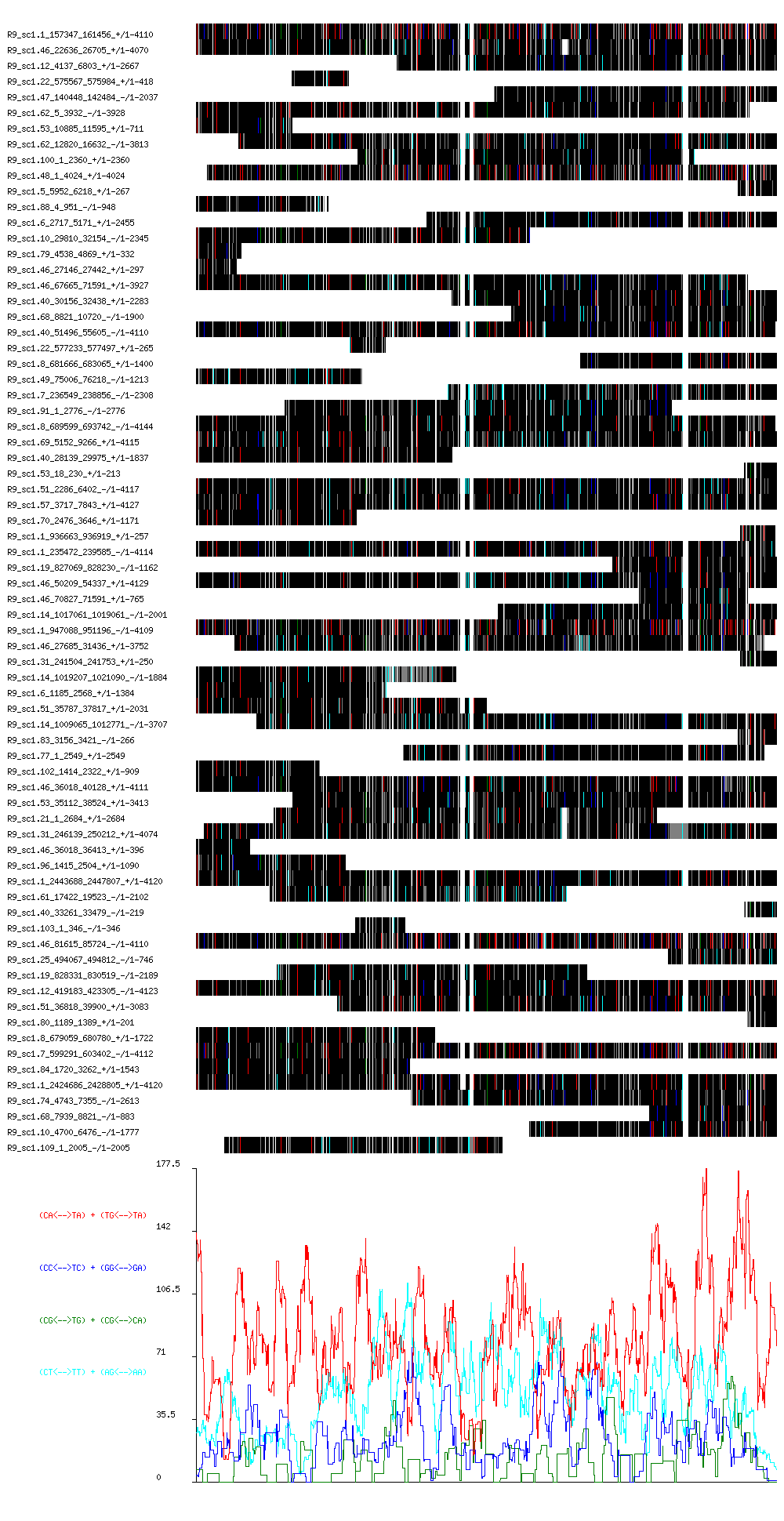

Supplement: Additional file 6 — List of predicted functional RecQ helicases in the S. nodorum genome. [file 1471-2164-11-655-S6.ZIP › consensus/r9_aln_2009_8_19_15_28_52_input.gif]

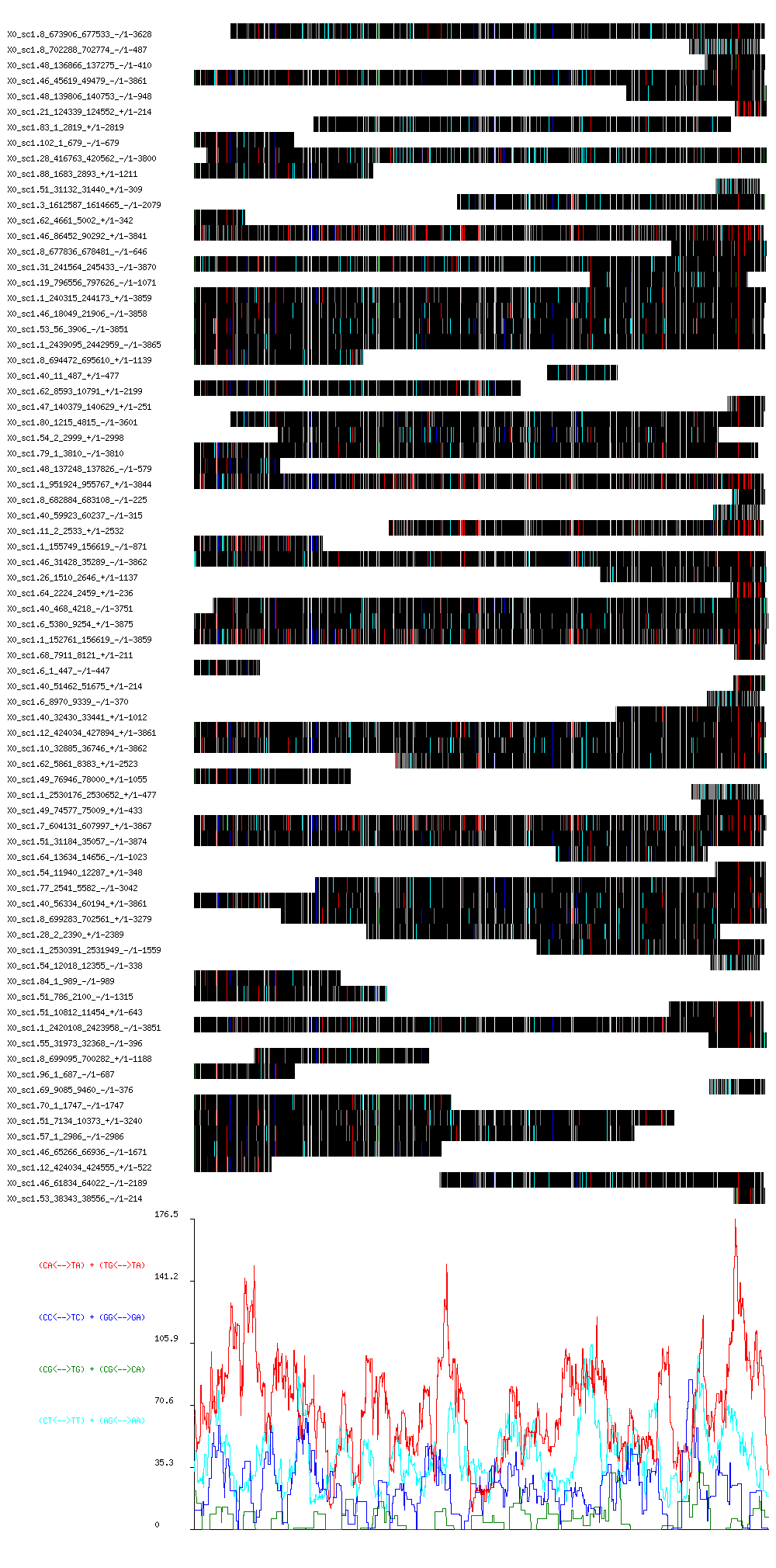

Supplement: Additional file 6 — List of predicted functional RecQ helicases in the S. nodorum genome. [file 1471-2164-11-655-S6.ZIP › consensus/x0_aln_2009_8_19_15_29_32_input.gif]

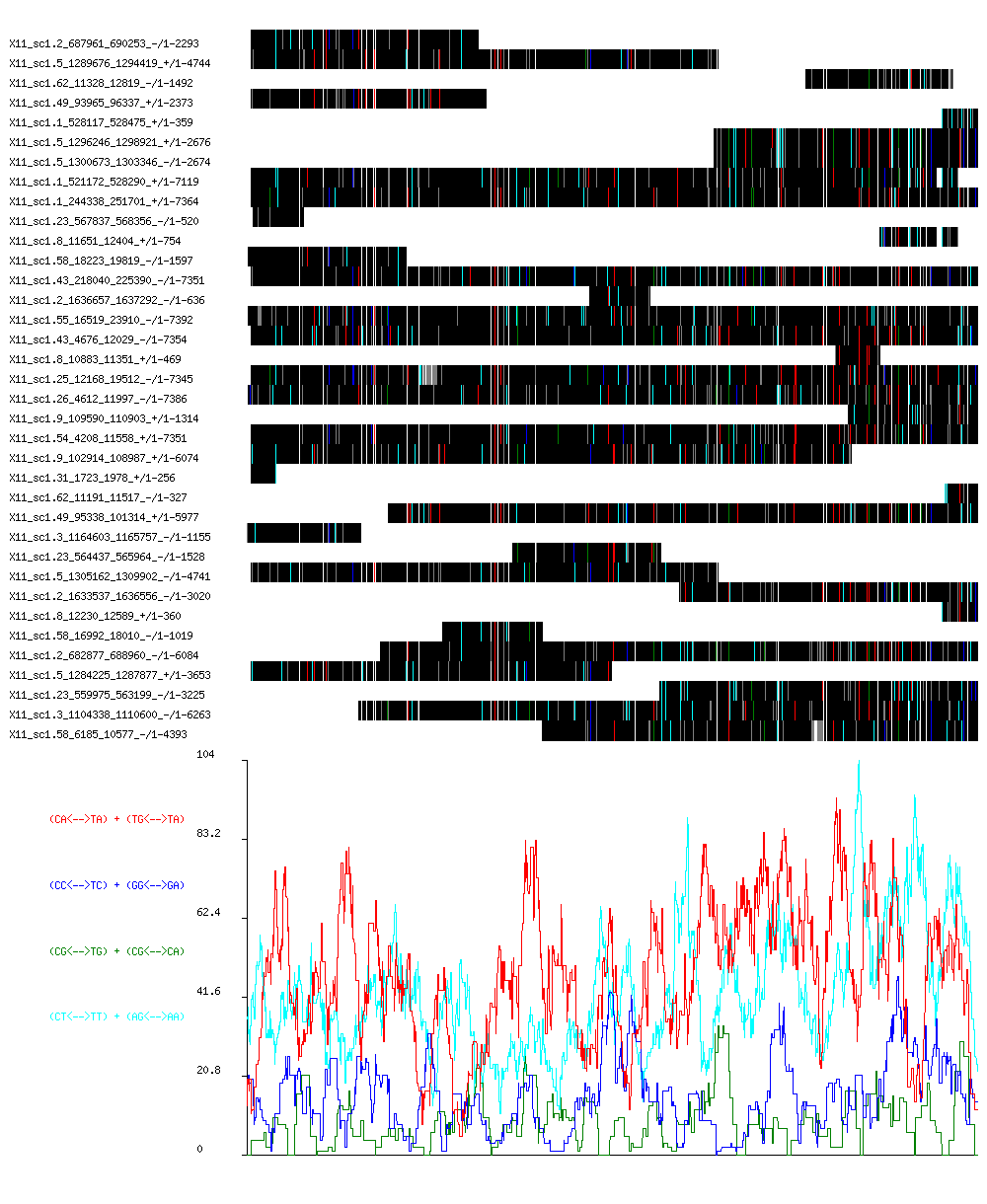

Supplement: Additional file 6 — List of predicted functional RecQ helicases in the S. nodorum genome. [file 1471-2164-11-655-S6.ZIP › consensus/x11_aln_2009_8_19_15_30_12_input.gif]

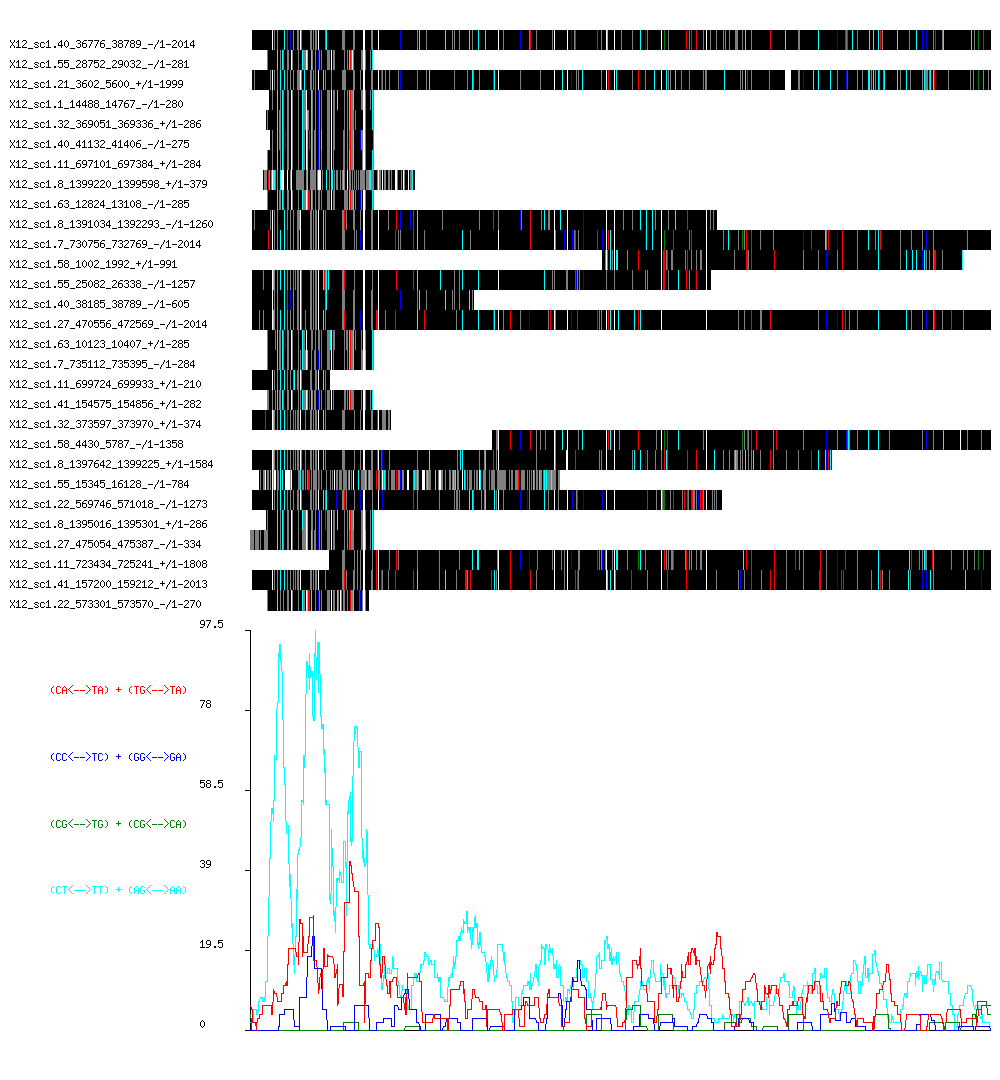

Supplement: Additional file 6 — List of predicted functional RecQ helicases in the S. nodorum genome. [file 1471-2164-11-655-S6.ZIP › consensus/x12_aln_2009_8_19_15_30_50_input.gif]

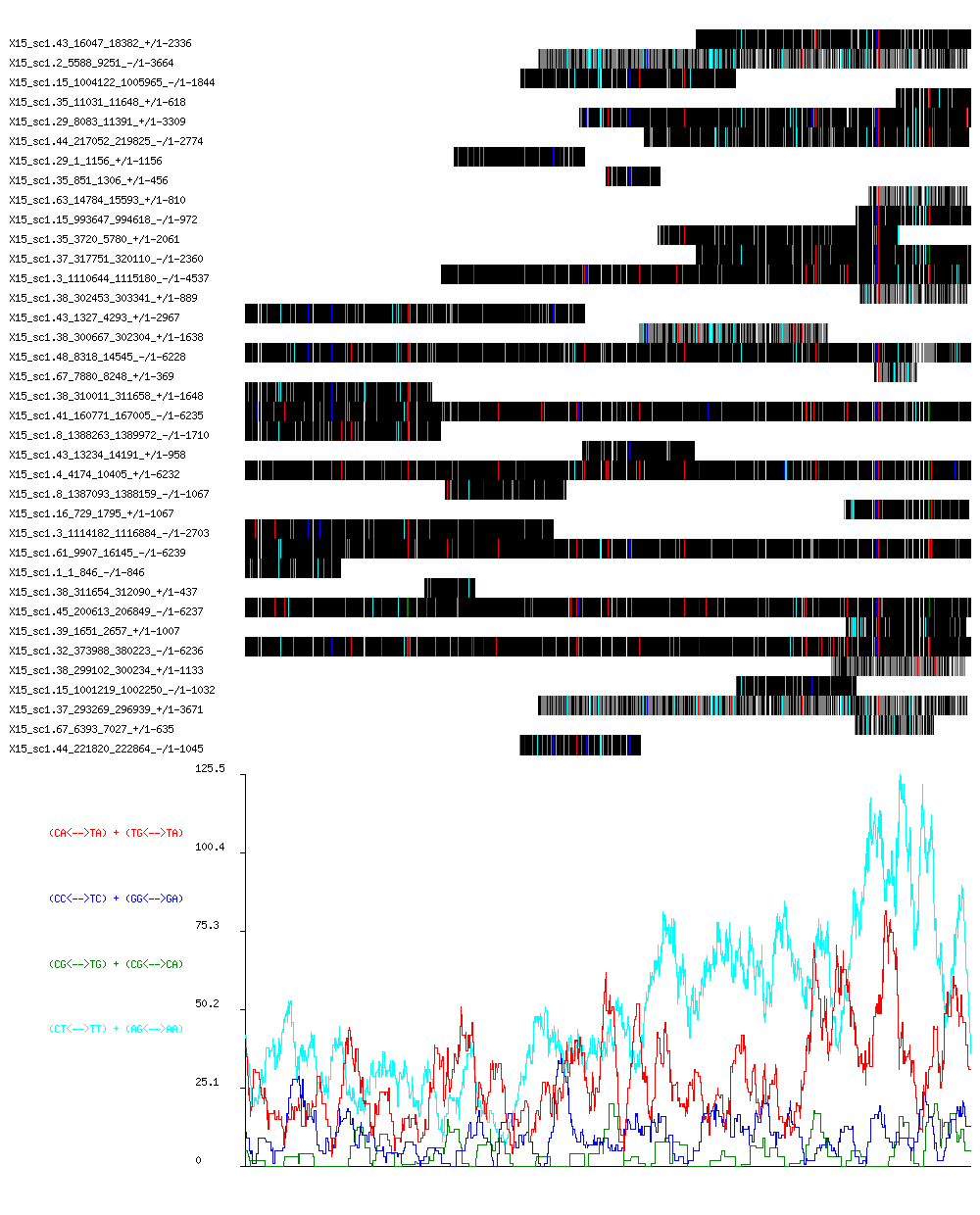

Supplement: Additional file 6 — List of predicted functional RecQ helicases in the S. nodorum genome. [file 1471-2164-11-655-S6.ZIP › consensus/x15_aln_2009_8_19_15_30_58_input.gif]

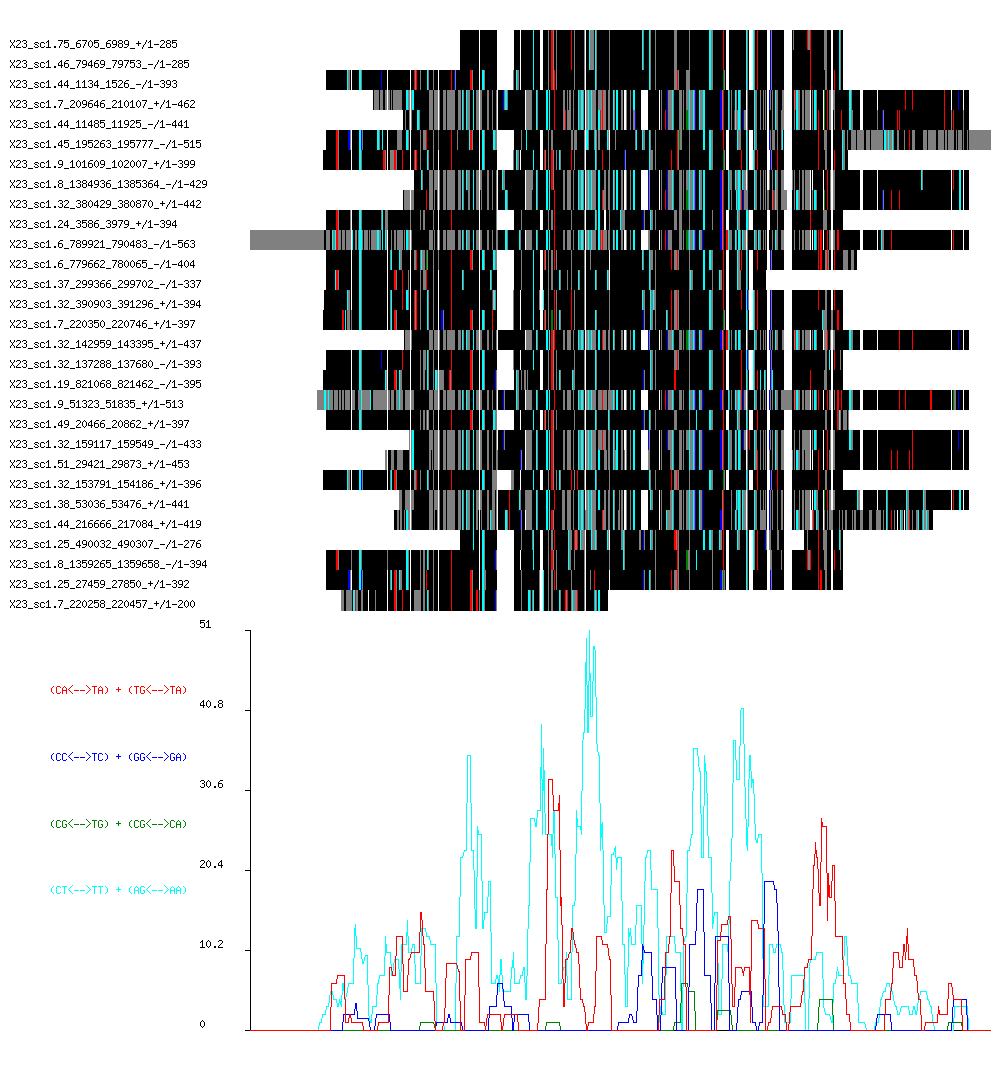

Supplement: Additional file 6 — List of predicted functional RecQ helicases in the S. nodorum genome. [file 1471-2164-11-655-S6.ZIP › consensus/x23_aln_2009_8_19_15_31_32_input.gif]

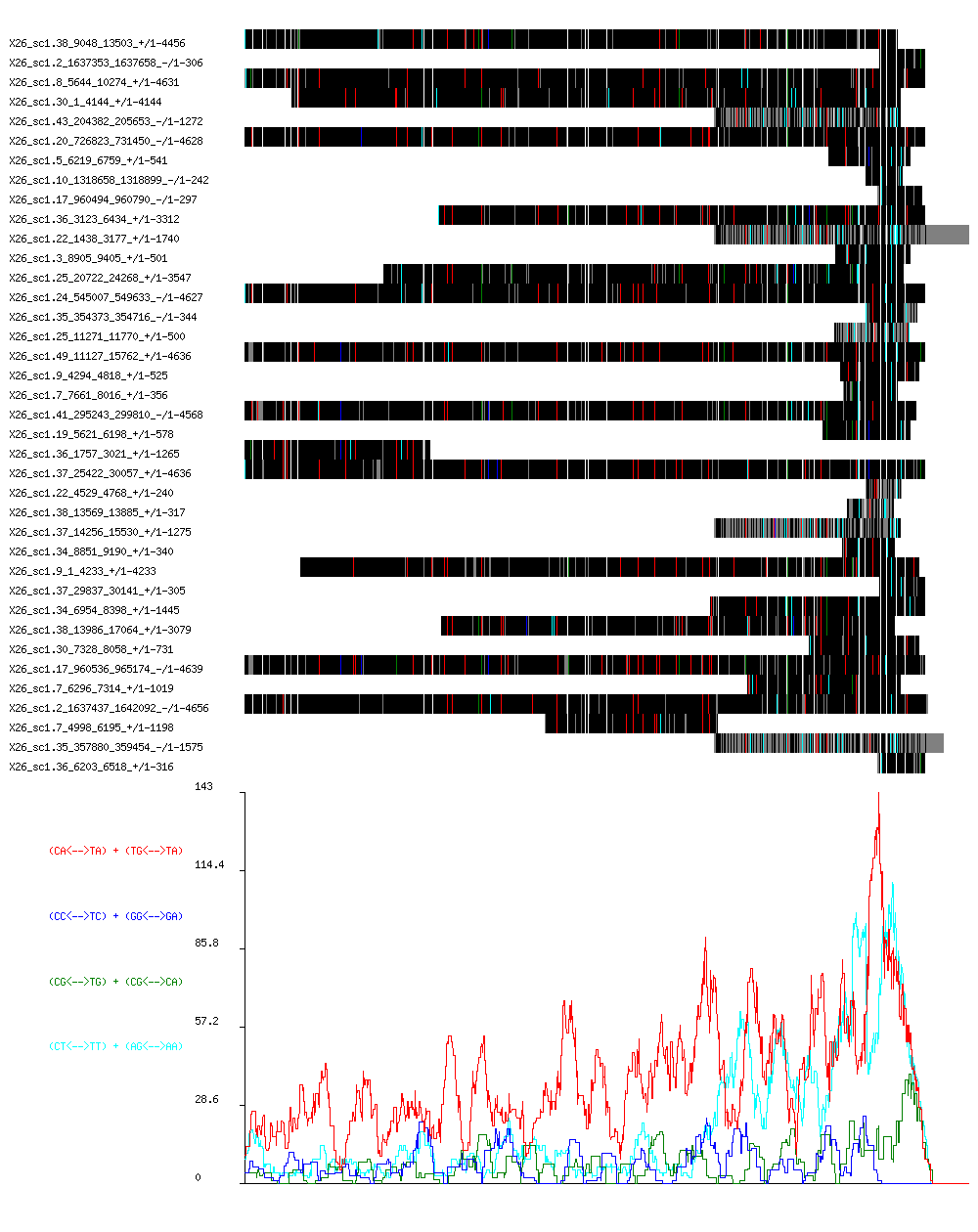

Supplement: Additional file 6 — List of predicted functional RecQ helicases in the S. nodorum genome. [file 1471-2164-11-655-S6.ZIP › consensus/x26_aln_2009_8_19_15_31_34_input.gif]

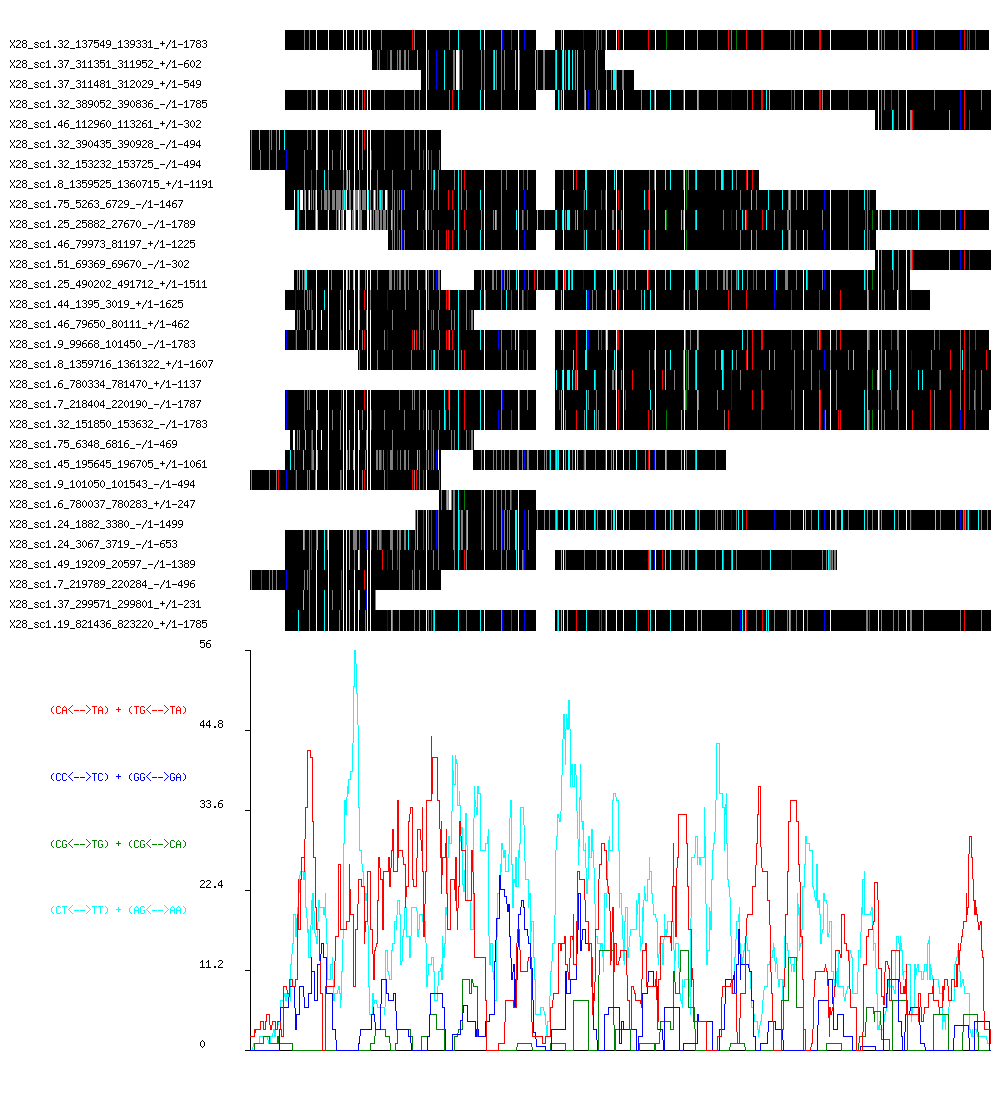

Supplement: Additional file 6 — List of predicted functional RecQ helicases in the S. nodorum genome. [file 1471-2164-11-655-S6.ZIP › consensus/x28_aln_2009_8_19_15_32_00_input.gif]

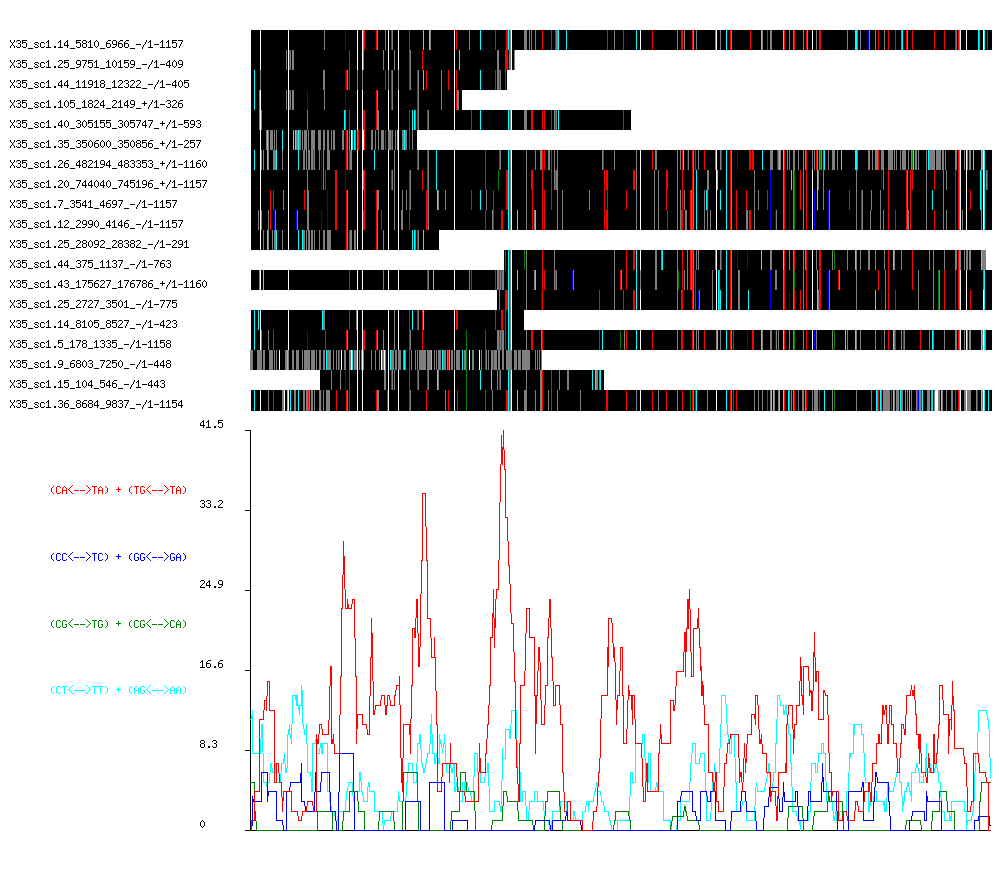

Supplement: Additional file 6 — List of predicted functional RecQ helicases in the S. nodorum genome. [file 1471-2164-11-655-S6.ZIP › consensus/x35_aln_2009_8_19_15_32_08_input.gif]

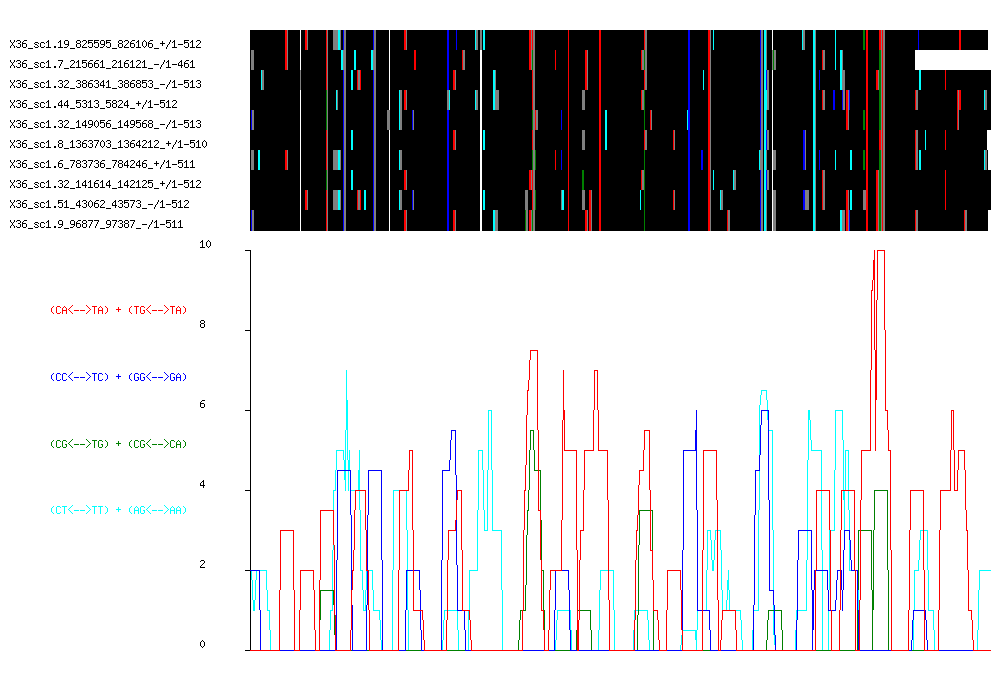

Supplement: Additional file 6 — List of predicted functional RecQ helicases in the S. nodorum genome. [file 1471-2164-11-655-S6.ZIP › consensus/x36_aln_2009_8_19_15_32_11_input.gif]

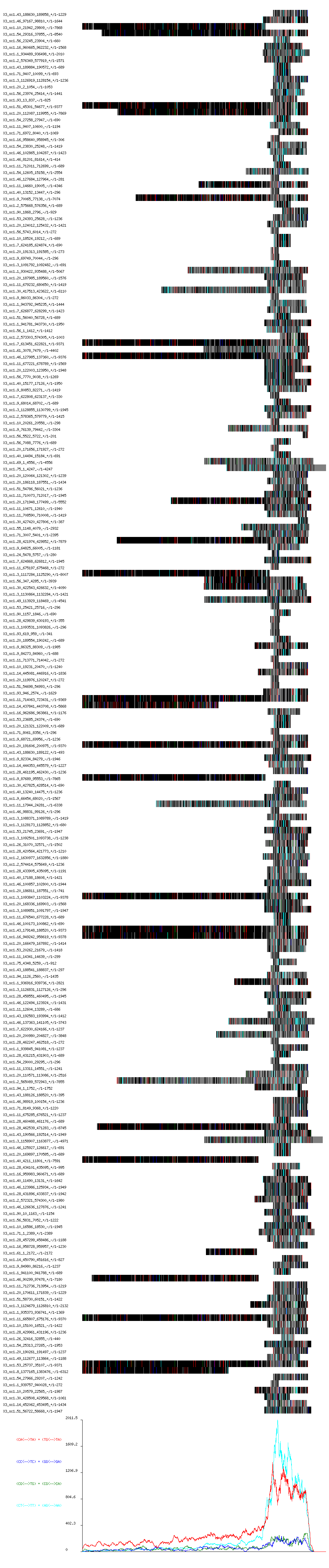

Supplement: Additional file 6 — List of predicted functional RecQ helicases in the S. nodorum genome. [file 1471-2164-11-655-S6.ZIP › consensus/x3_aln_2009_8_19_15_32_13_input.gif]

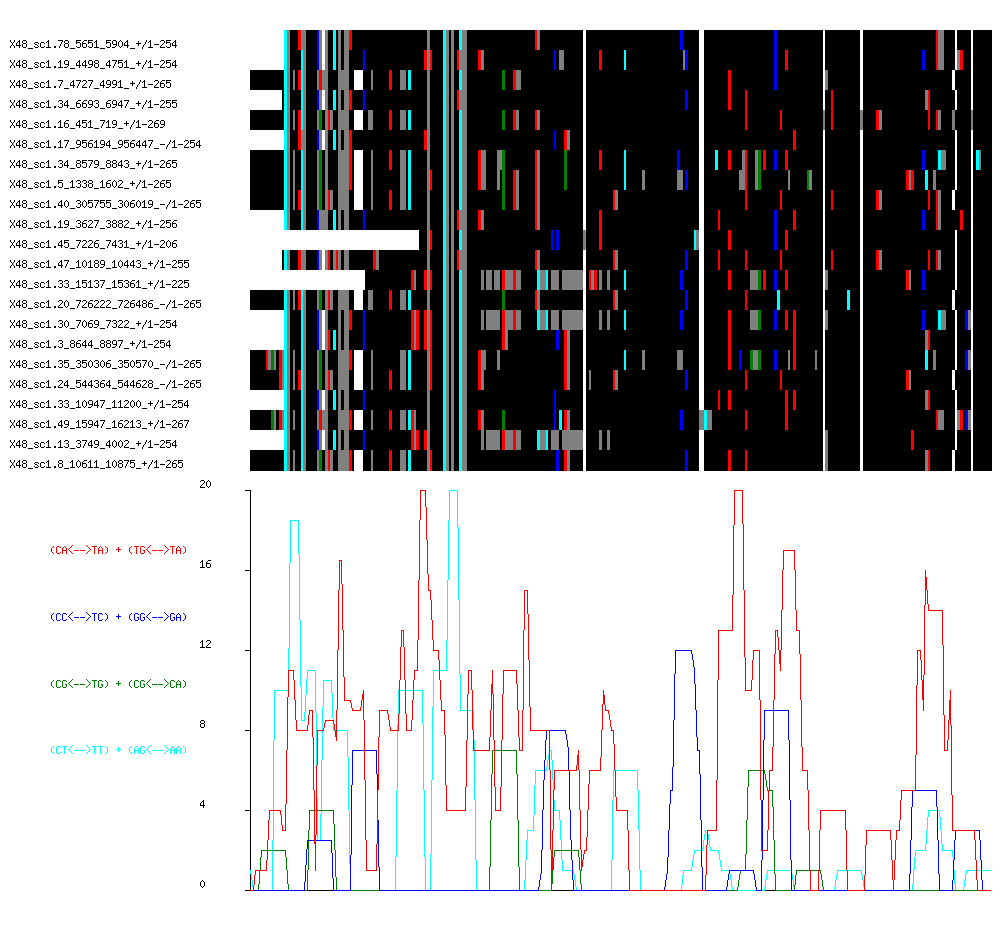

Supplement: Additional file 6 — List of predicted functional RecQ helicases in the S. nodorum genome. [file 1471-2164-11-655-S6.ZIP › consensus/x48_aln_2009_8_19_15_37_16_input.gif]

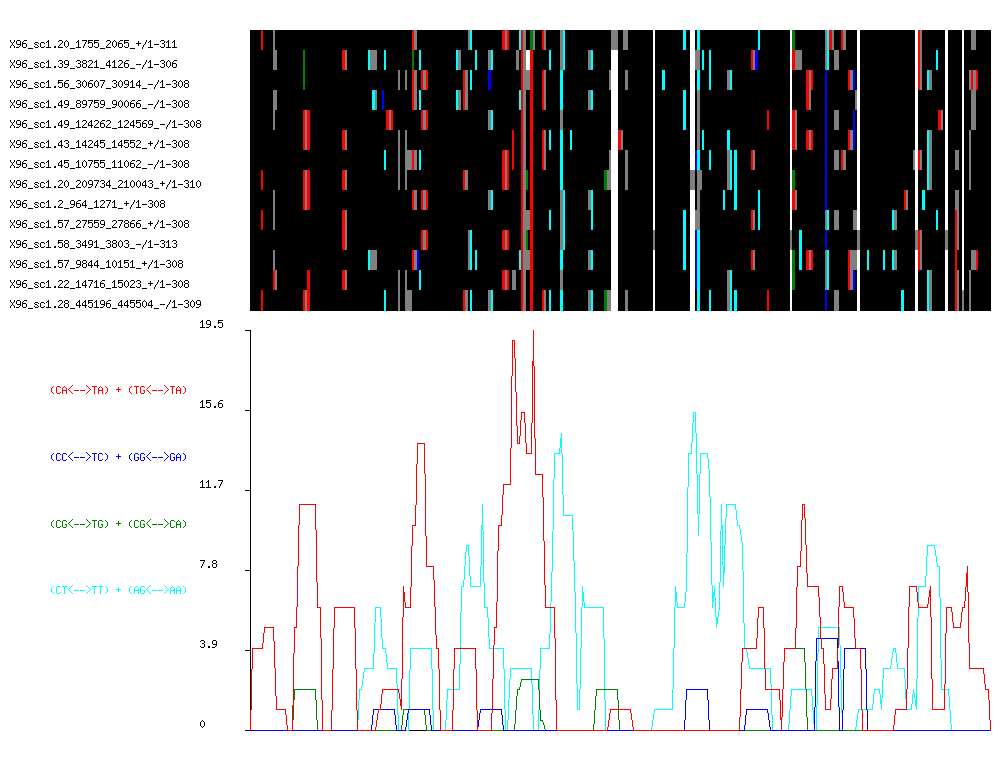

Supplement: Additional file 6 — List of predicted functional RecQ helicases in the S. nodorum genome. [file 1471-2164-11-655-S6.ZIP › consensus/x96_aln_2009_8_19_15_37_17_input.gif]

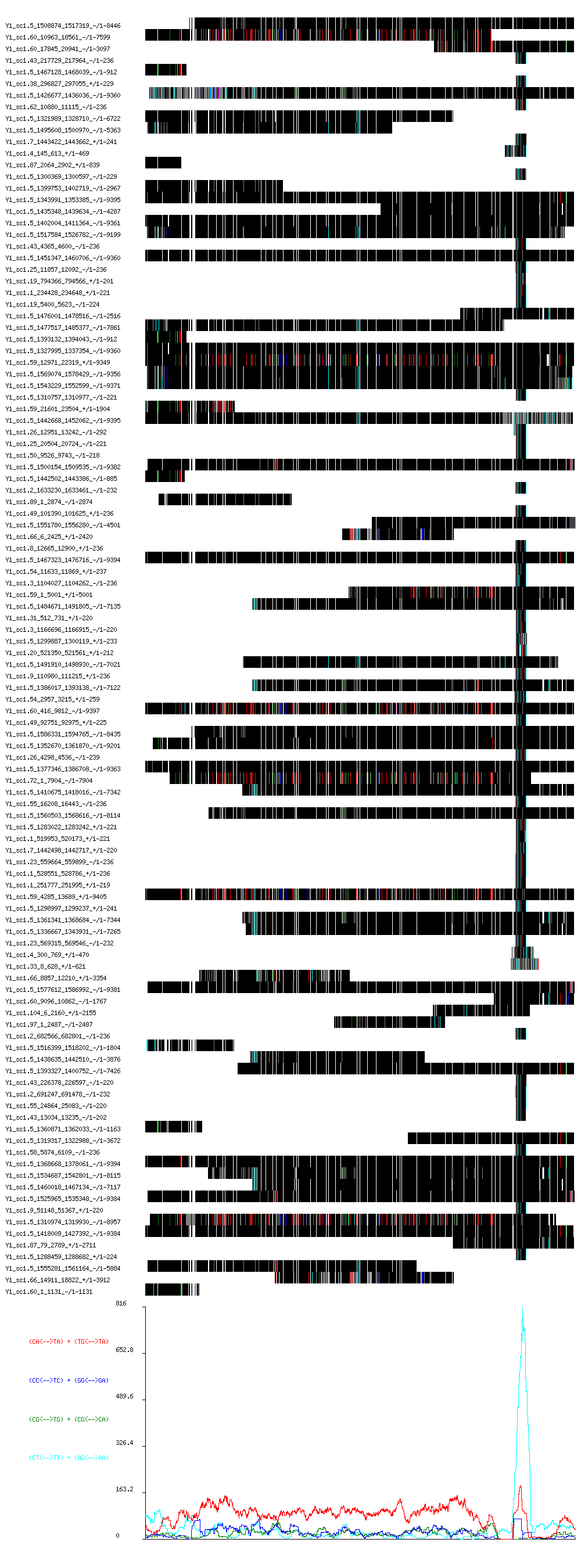

Supplement: Additional file 6 — List of predicted functional RecQ helicases in the S. nodorum genome. [file 1471-2164-11-655-S6.ZIP › consensus/y1_aln_2009_8_19_15_37_18_input.gif]

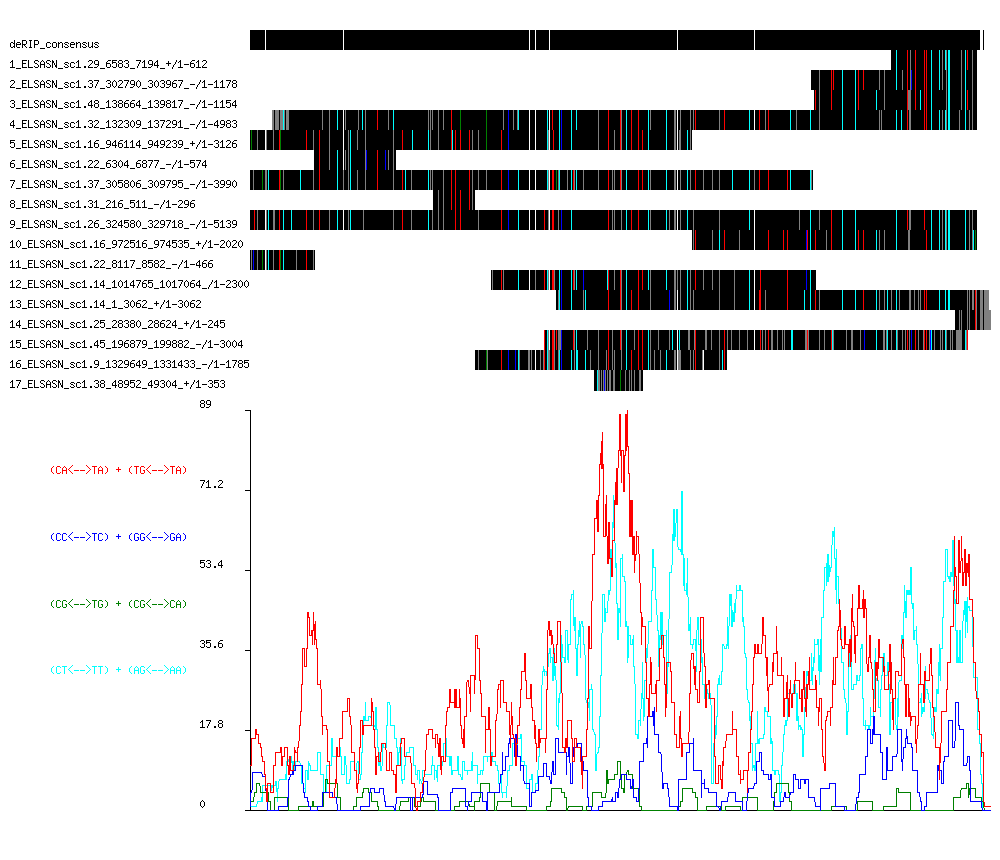

Supplement: Additional file 6 — List of predicted functional RecQ helicases in the S. nodorum genome. [file 1471-2164-11-655-S6.ZIP › derip/elsa_aln_derip_2009_8_19_15_39_44_input.gif]

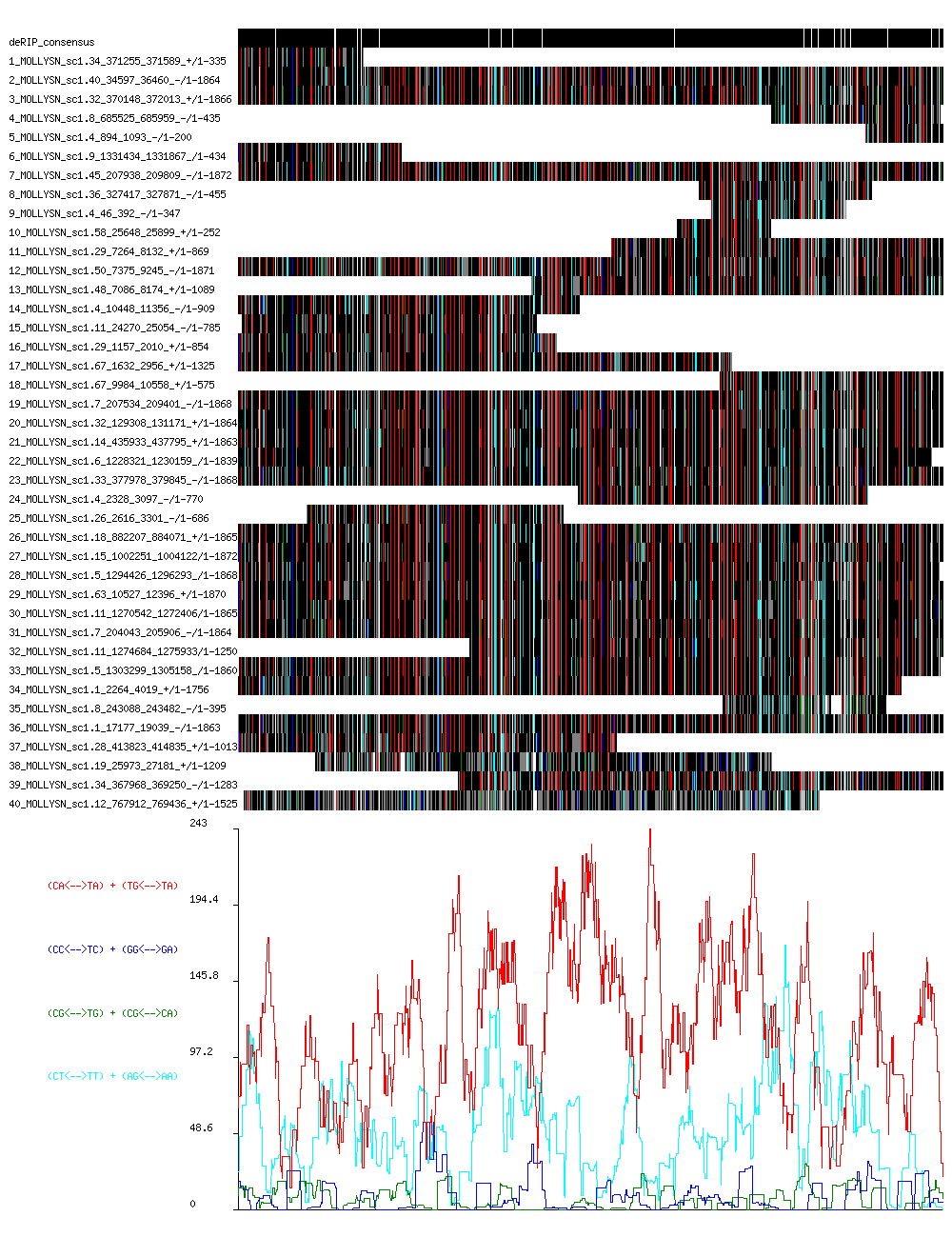

Supplement: Additional file 6 — List of predicted functional RecQ helicases in the S. nodorum genome. [file 1471-2164-11-655-S6.ZIP › derip/molly_aln_derip_2009_8_19_15_39_59_input.gif]

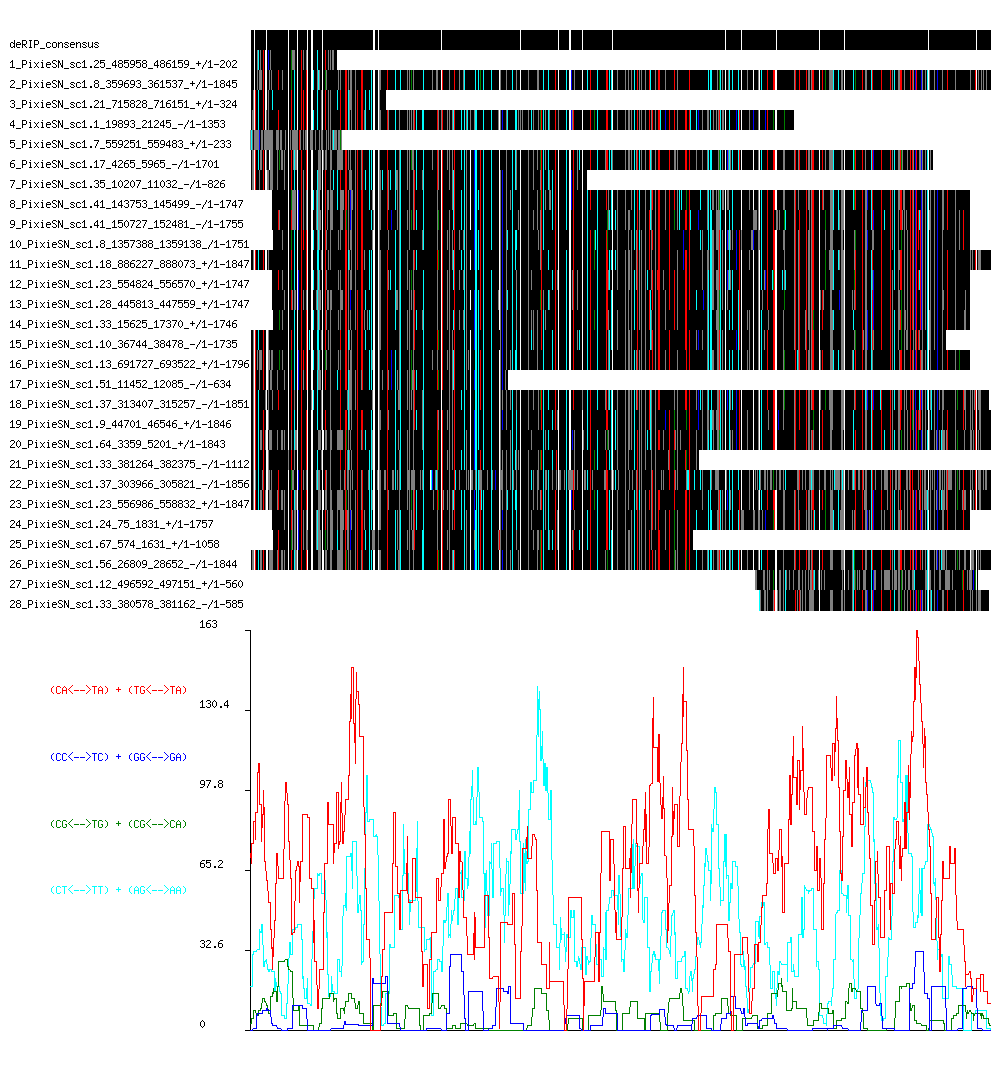

Supplement: Additional file 6 — List of predicted functional RecQ helicases in the S. nodorum genome. [file 1471-2164-11-655-S6.ZIP › derip/pixie_aln_derip_2009_8_19_15_40_09_input.gif]

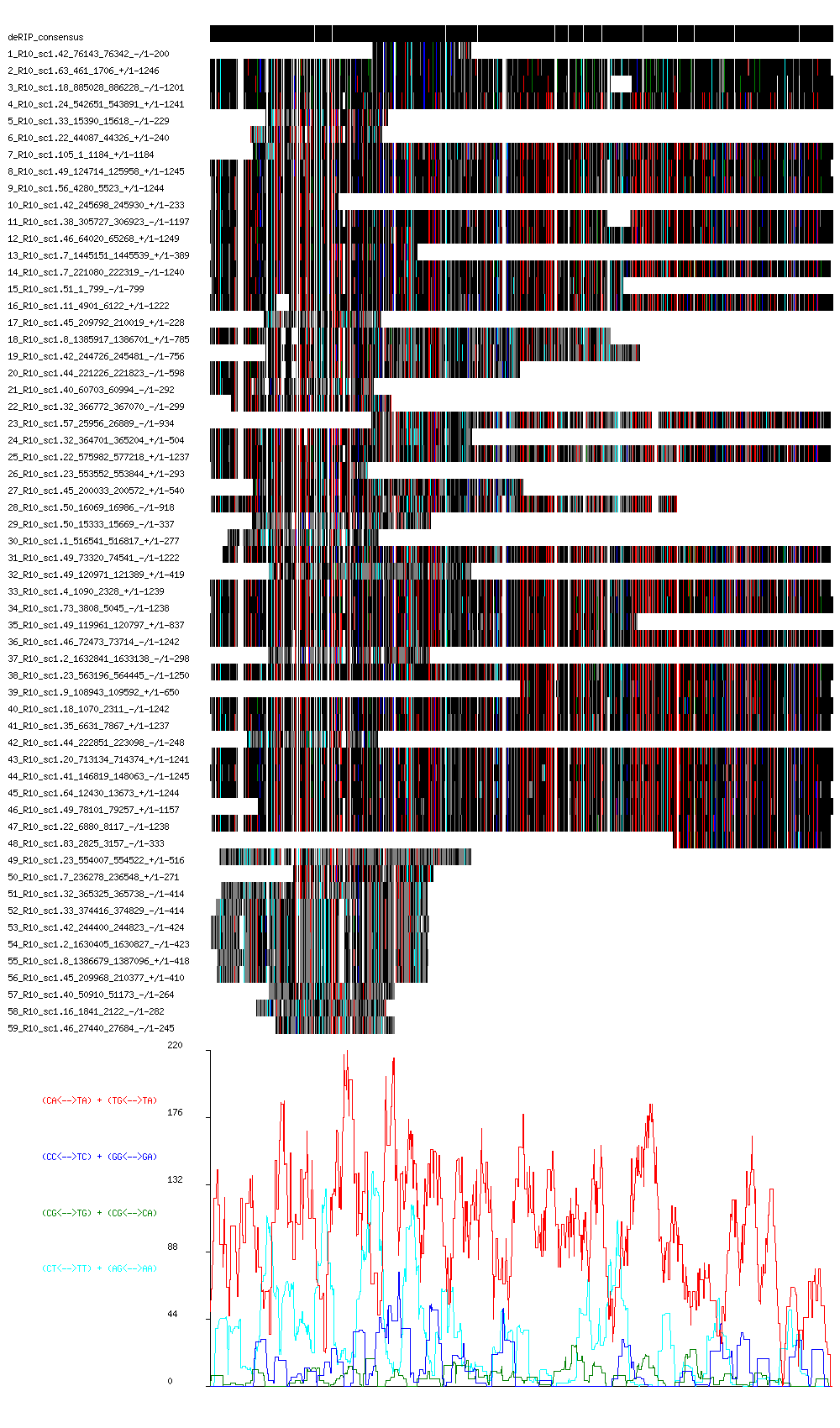

Supplement: Additional file 6 — List of predicted functional RecQ helicases in the S. nodorum genome. [file 1471-2164-11-655-S6.ZIP › derip/r10_aln_derip_2009_8_19_15_40_16_input.gif]

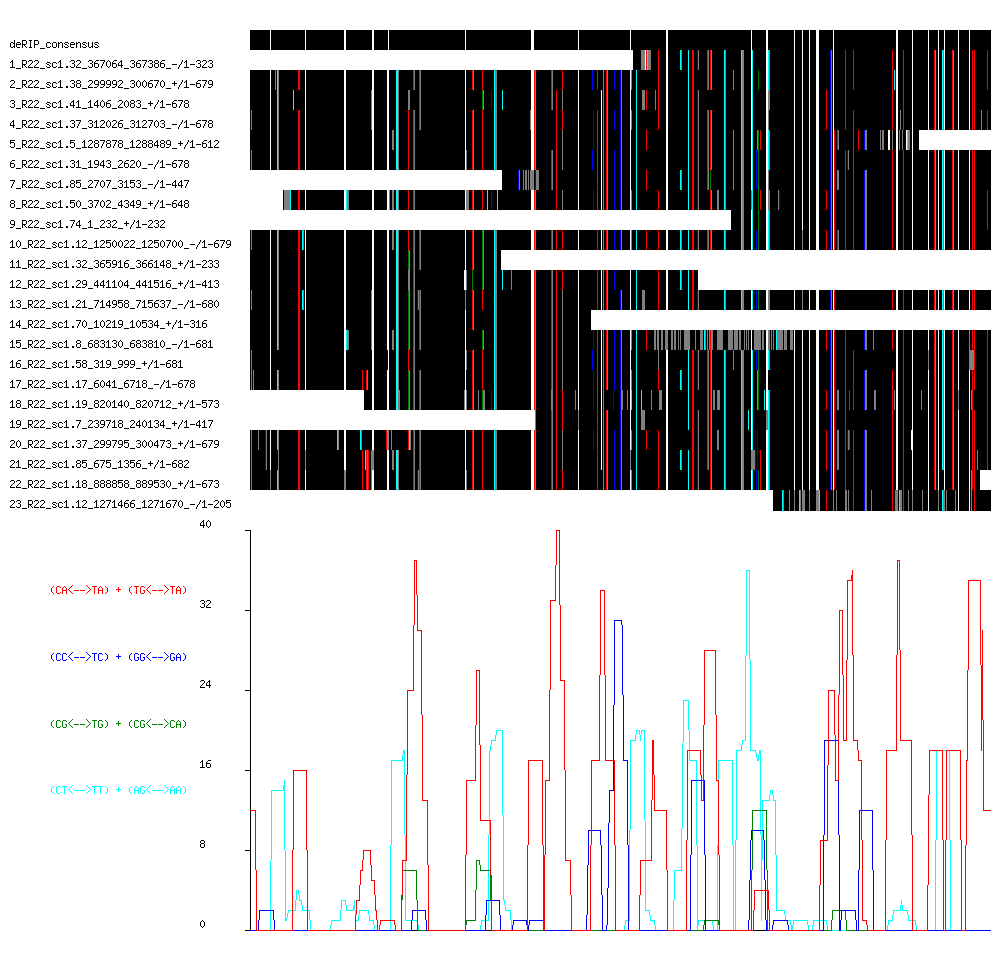

Supplement: Additional file 6 — List of predicted functional RecQ helicases in the S. nodorum genome. [file 1471-2164-11-655-S6.ZIP › derip/r22_aln_derip_2009_8_19_15_40_27_input.gif]

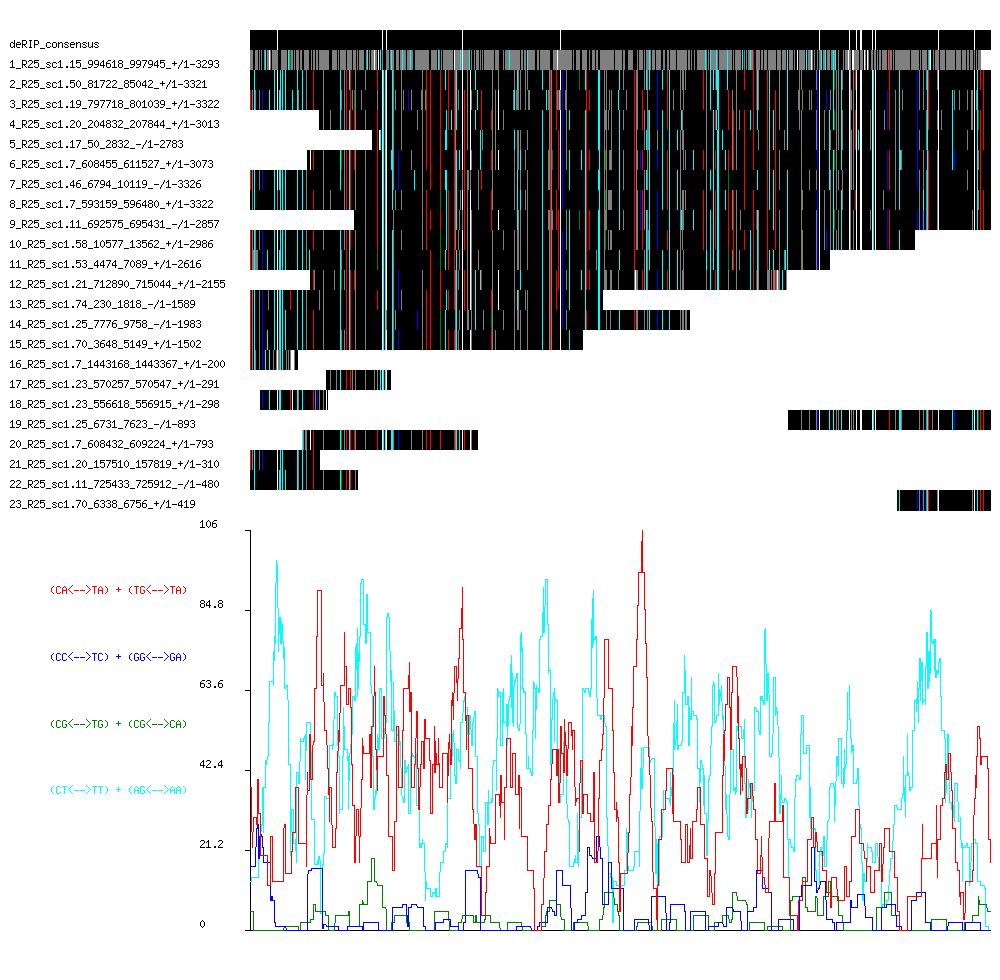

Supplement: Additional file 6 — List of predicted functional RecQ helicases in the S. nodorum genome. [file 1471-2164-11-655-S6.ZIP › derip/r25_aln_derip_2009_8_19_15_40_29_input.gif]

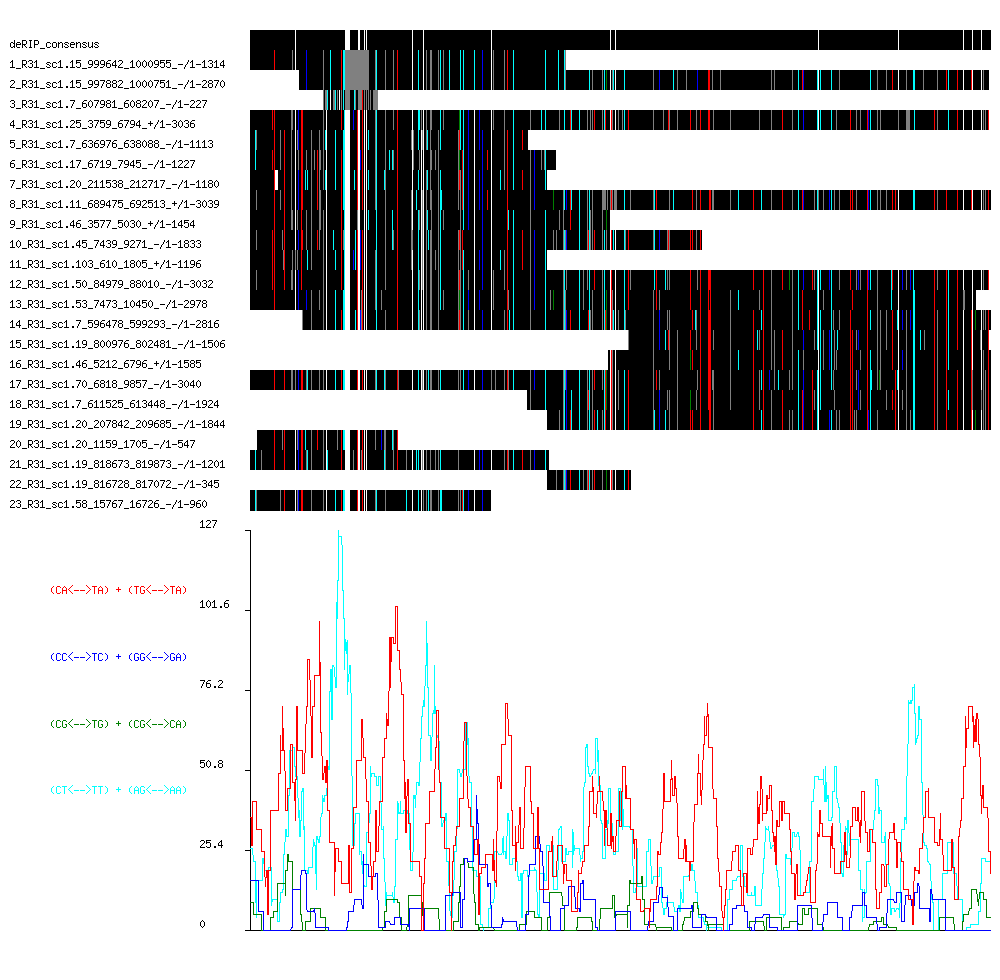

Supplement: Additional file 6 — List of predicted functional RecQ helicases in the S. nodorum genome. [file 1471-2164-11-655-S6.ZIP › derip/r31_aln_derip_2009_8_19_15_40_40_input.gif]

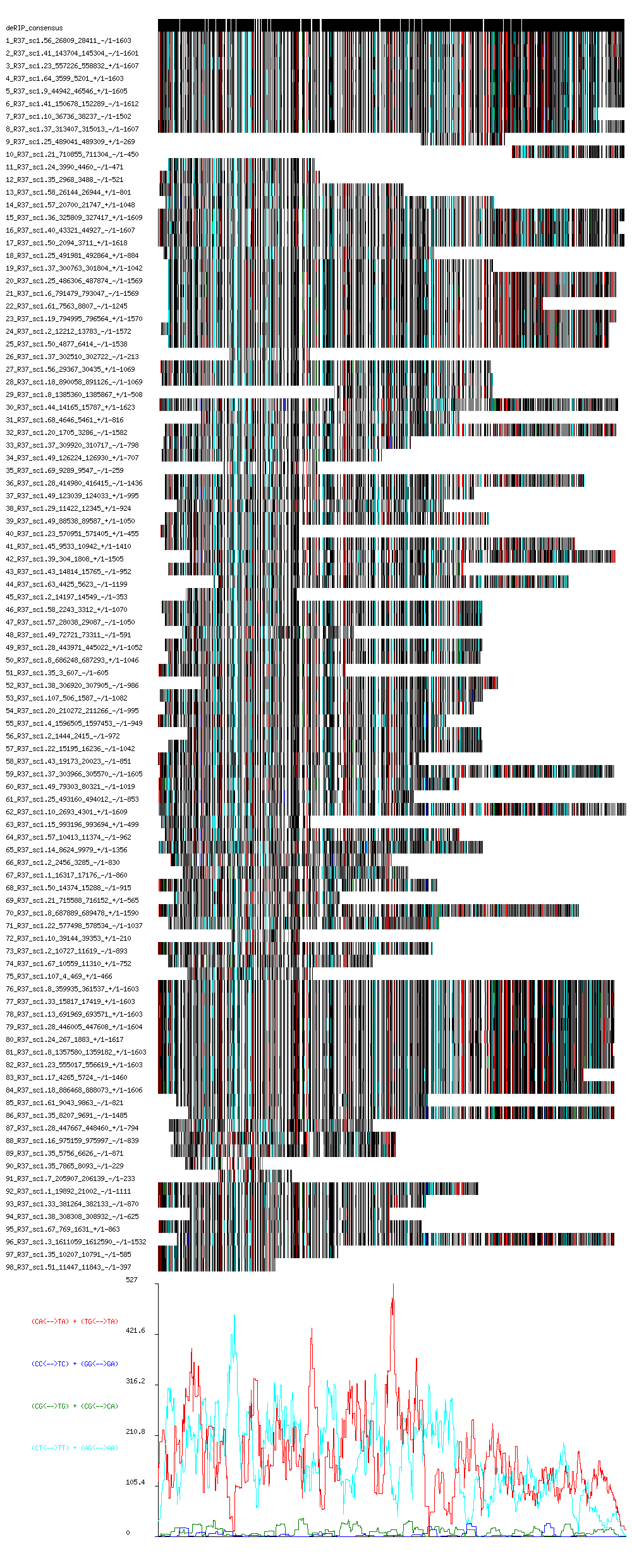

Supplement: Additional file 6 — List of predicted functional RecQ helicases in the S. nodorum genome. [file 1471-2164-11-655-S6.ZIP › derip/r37_aln_derip_2009_8_19_15_40_50_input.gif]

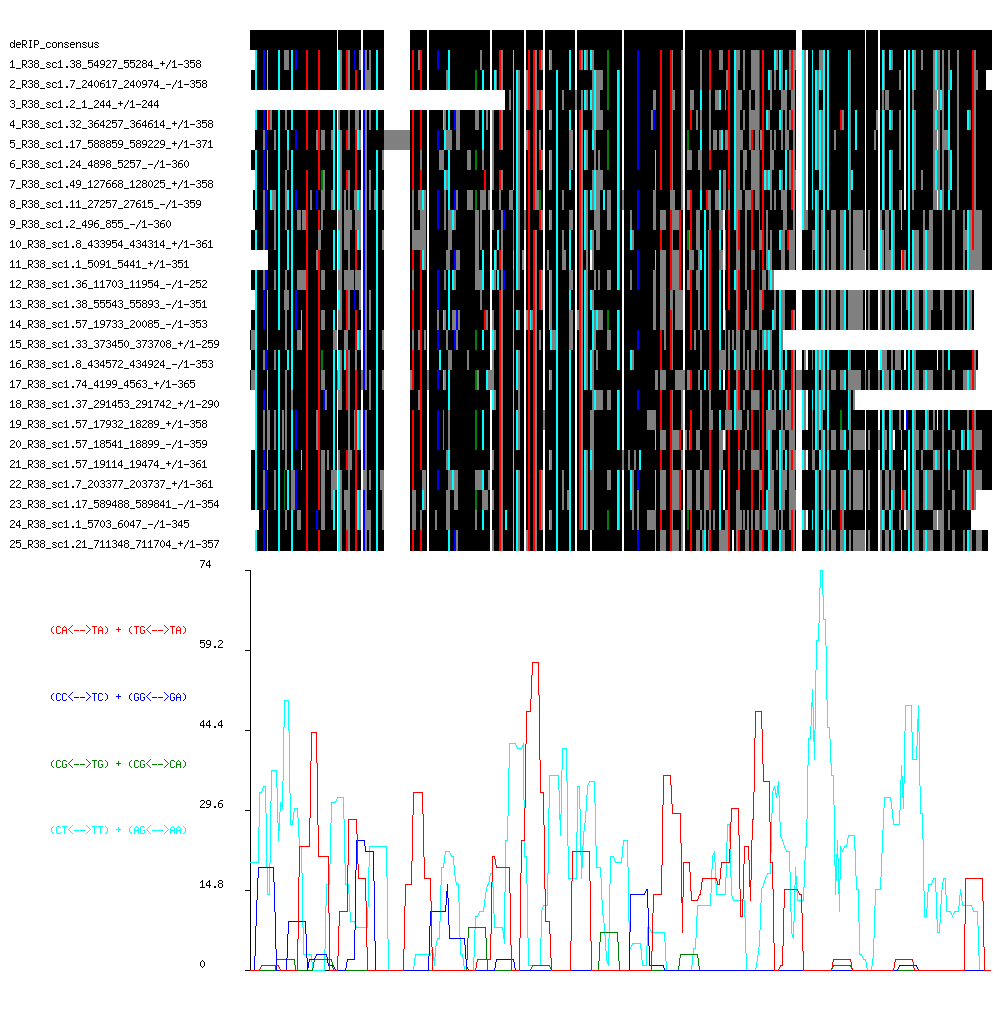

Supplement: Additional file 6 — List of predicted functional RecQ helicases in the S. nodorum genome. [file 1471-2164-11-655-S6.ZIP › derip/r38_aln_derip_2009_8_19_15_41_19_input.gif]

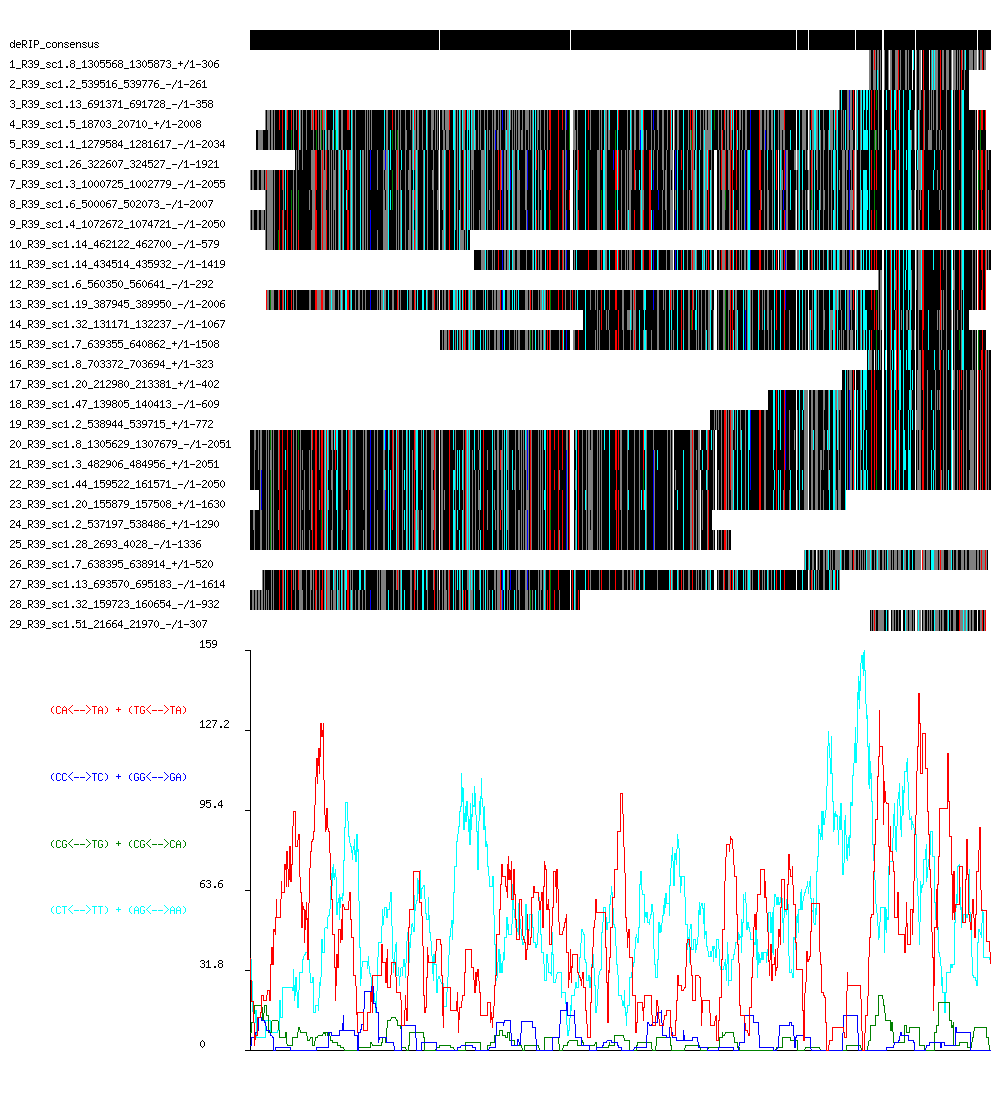

Supplement: Additional file 6 — List of predicted functional RecQ helicases in the S. nodorum genome. [file 1471-2164-11-655-S6.ZIP › derip/r39_aln_derip_2009_8_19_15_41_20_input.gif]

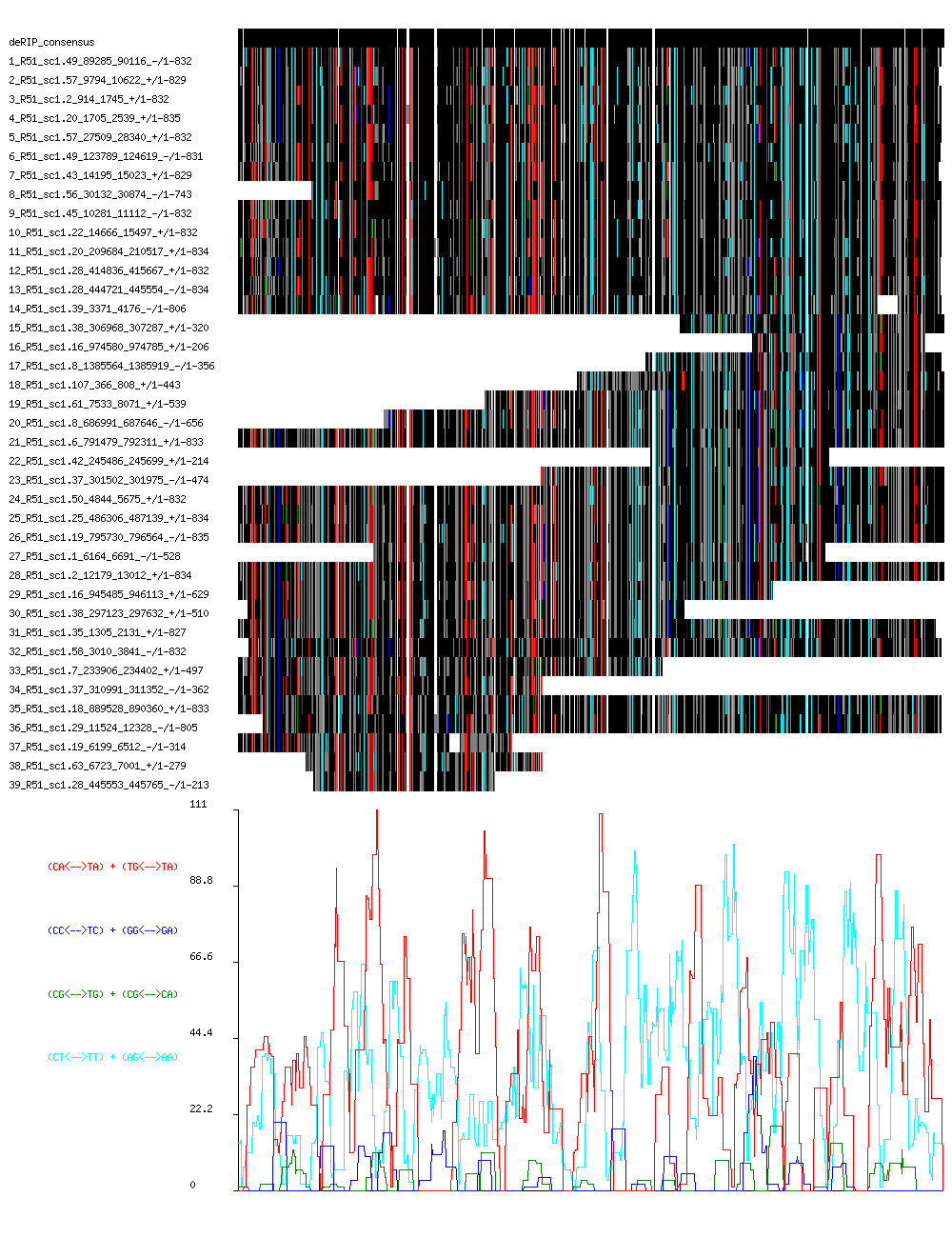

Supplement: Additional file 6 — List of predicted functional RecQ helicases in the S. nodorum genome. [file 1471-2164-11-655-S6.ZIP › derip/r51_aln_derip_2009_8_19_15_41_28_input.gif]

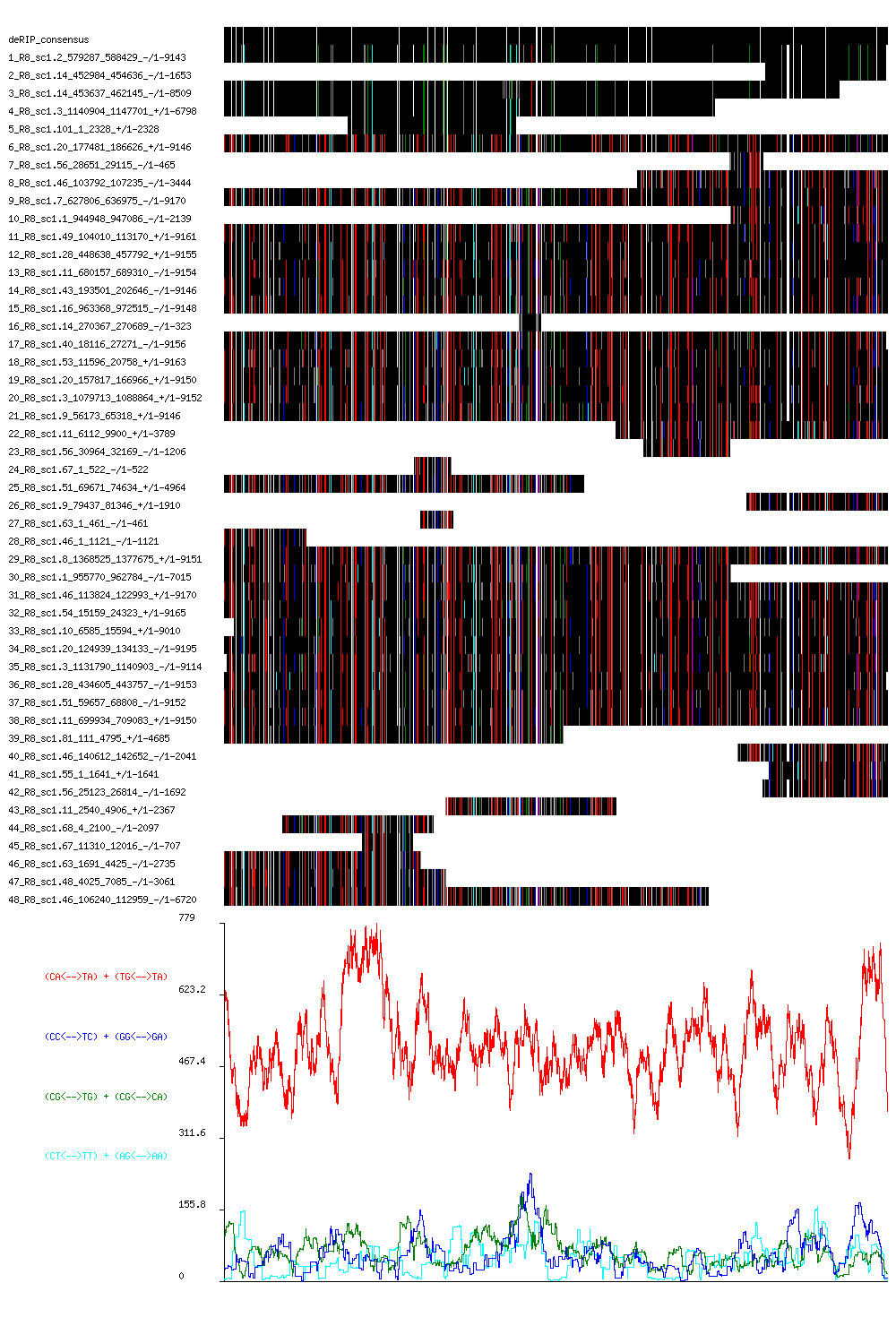

Supplement: Additional file 6 — List of predicted functional RecQ helicases in the S. nodorum genome. [file 1471-2164-11-655-S6.ZIP › derip/r8_aln_derip_2009_8_19_15_41_33_input.gif]

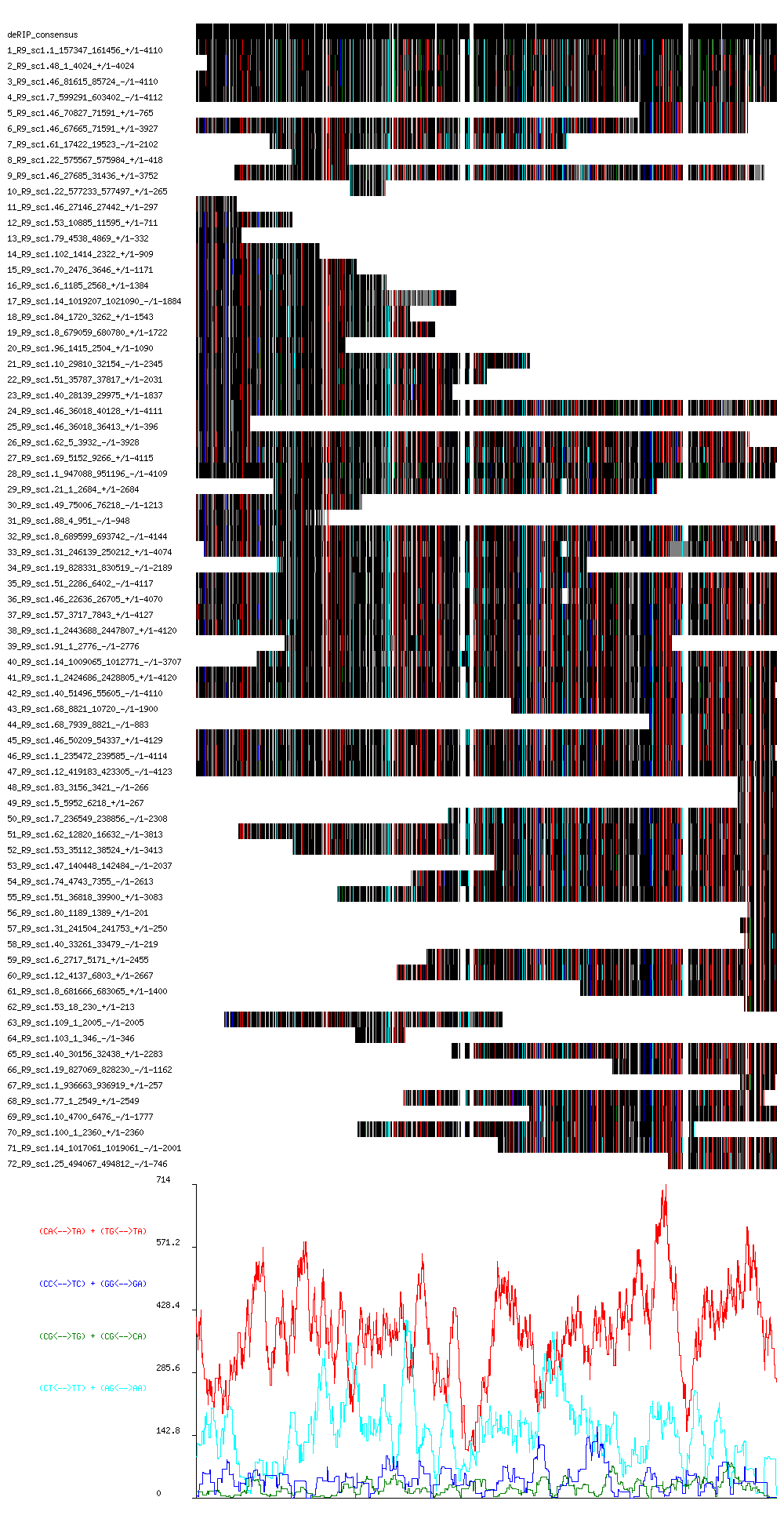

Supplement: Additional file 6 — List of predicted functional RecQ helicases in the S. nodorum genome. [file 1471-2164-11-655-S6.ZIP › derip/r9_aln_derip_2009_8_19_15_42_38_input.gif]

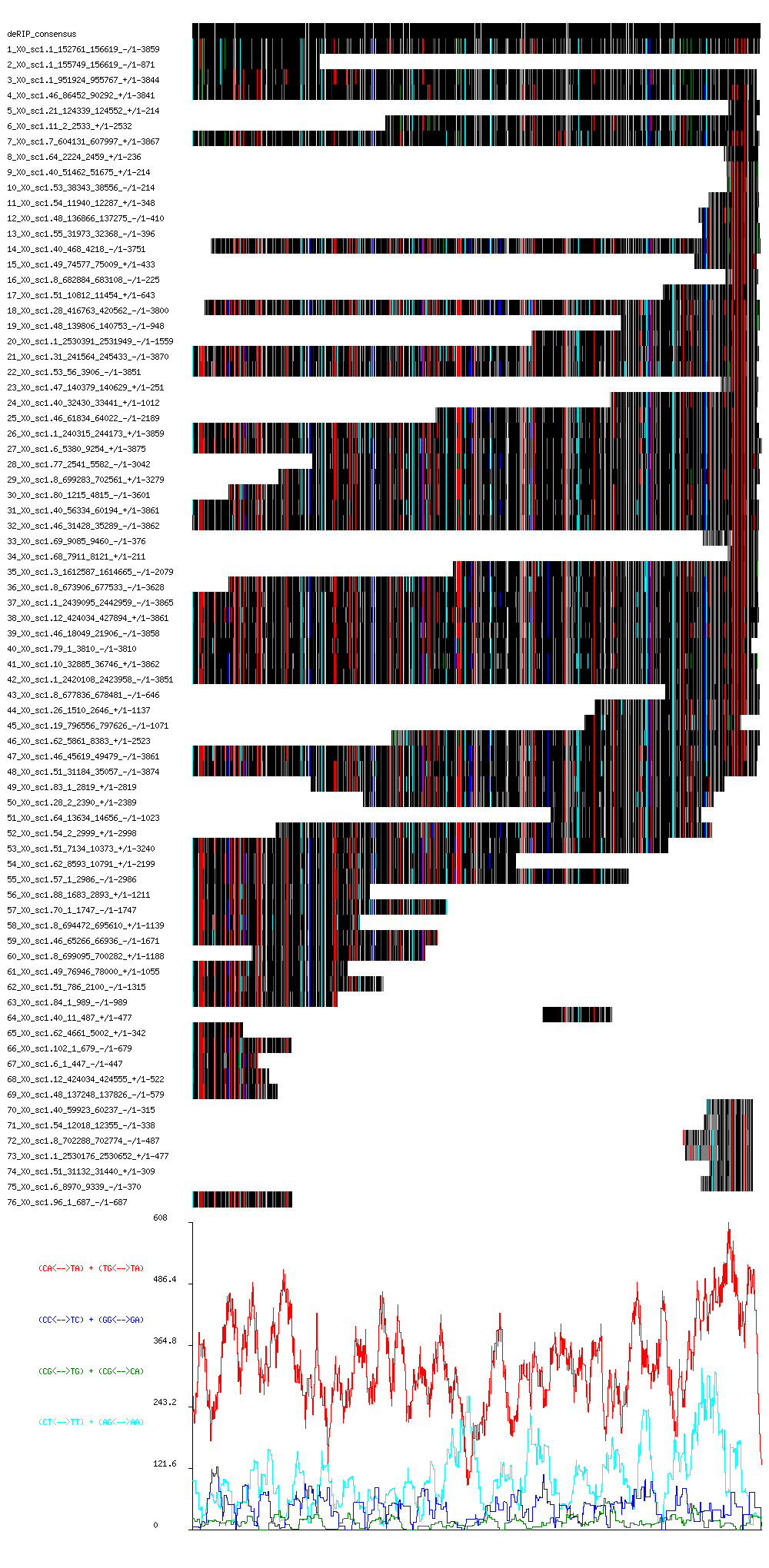

Supplement: Additional file 6 — List of predicted functional RecQ helicases in the S. nodorum genome. [file 1471-2164-11-655-S6.ZIP › derip/x0_aln_derip_2009_8_19_15_43_19_input.gif]

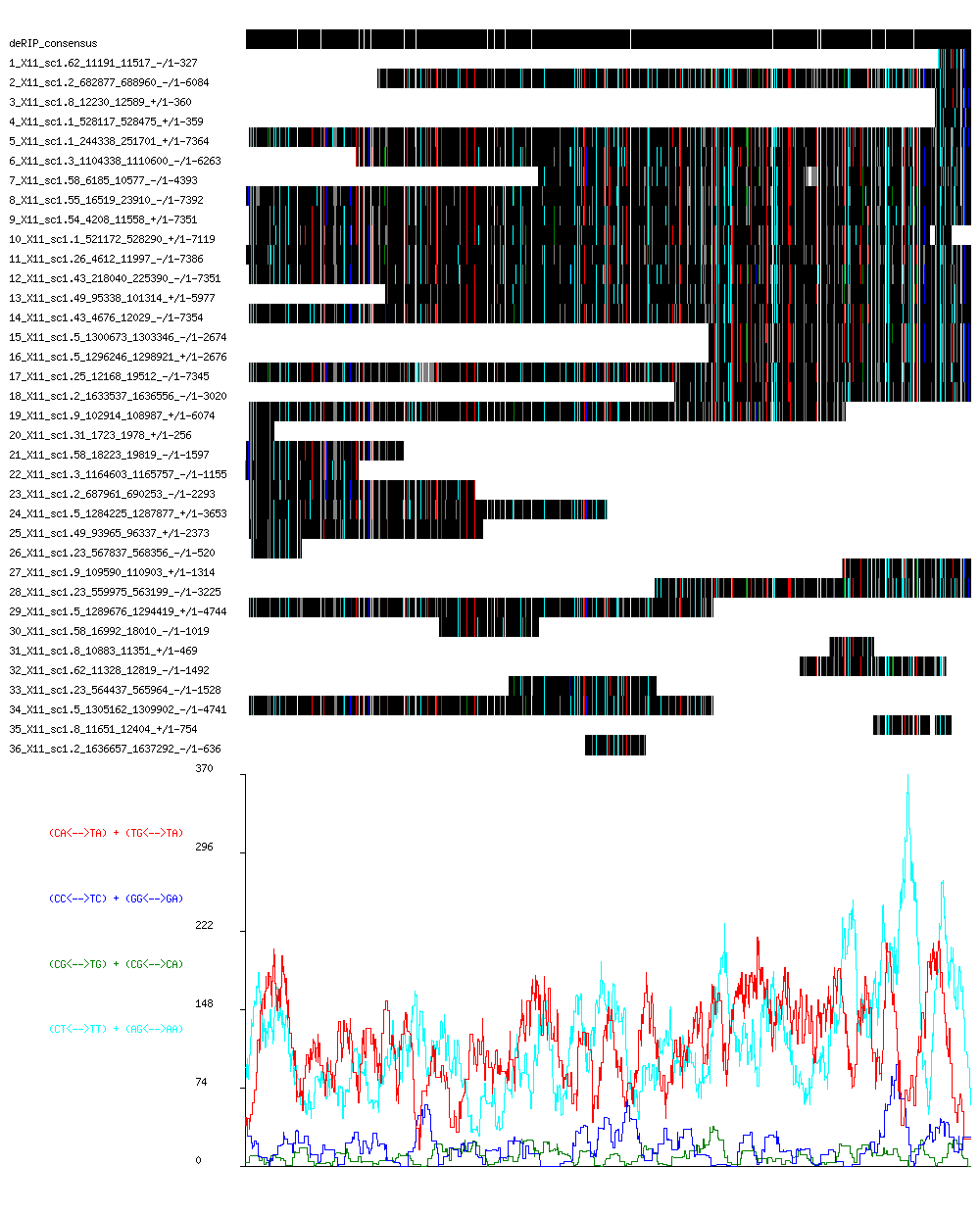

Supplement: Additional file 6 — List of predicted functional RecQ helicases in the S. nodorum genome. [file 1471-2164-11-655-S6.ZIP › derip/x11_aln_derip_2009_8_19_15_44_01_input.gif]

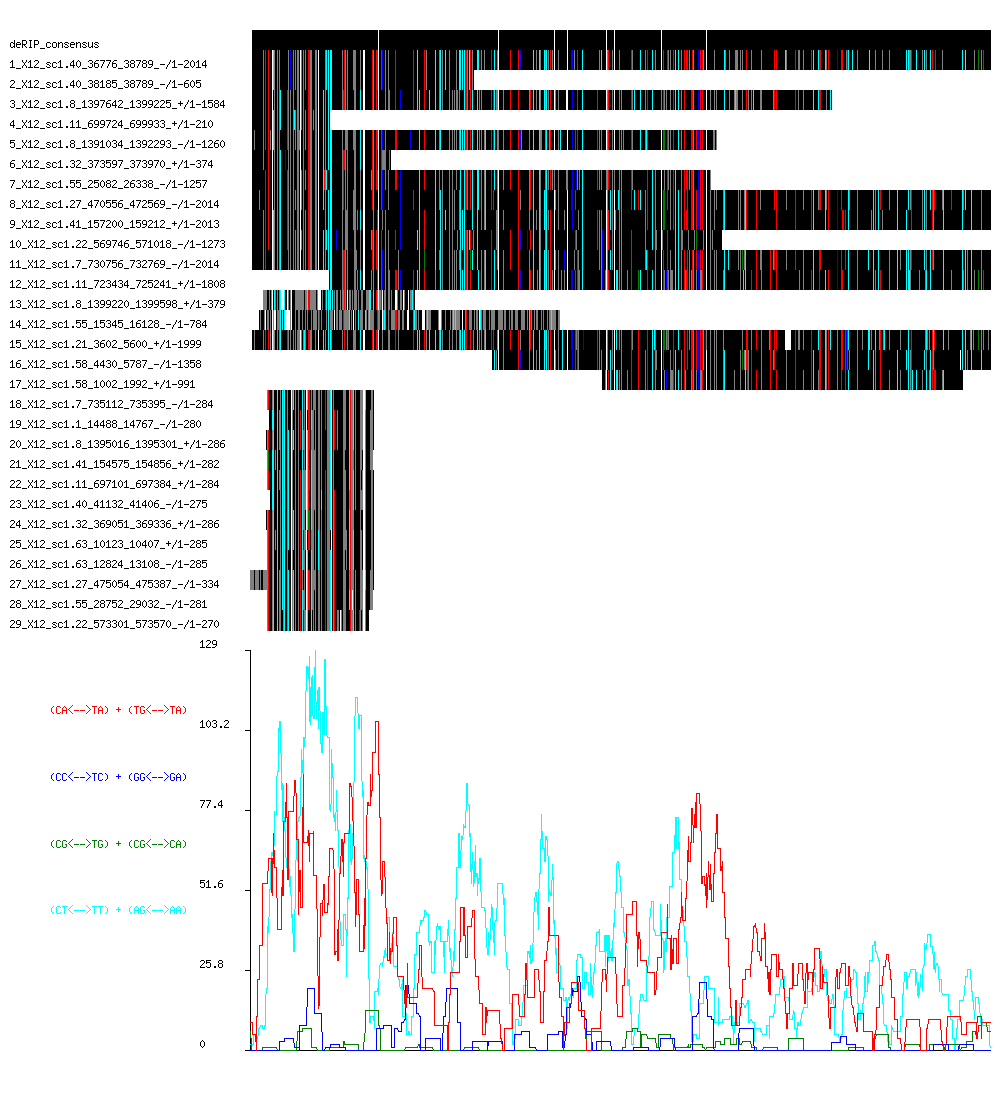

Supplement: Additional file 6 — List of predicted functional RecQ helicases in the S. nodorum genome. [file 1471-2164-11-655-S6.ZIP › derip/x12_aln_derip_2009_8_19_15_44_39_input.gif]

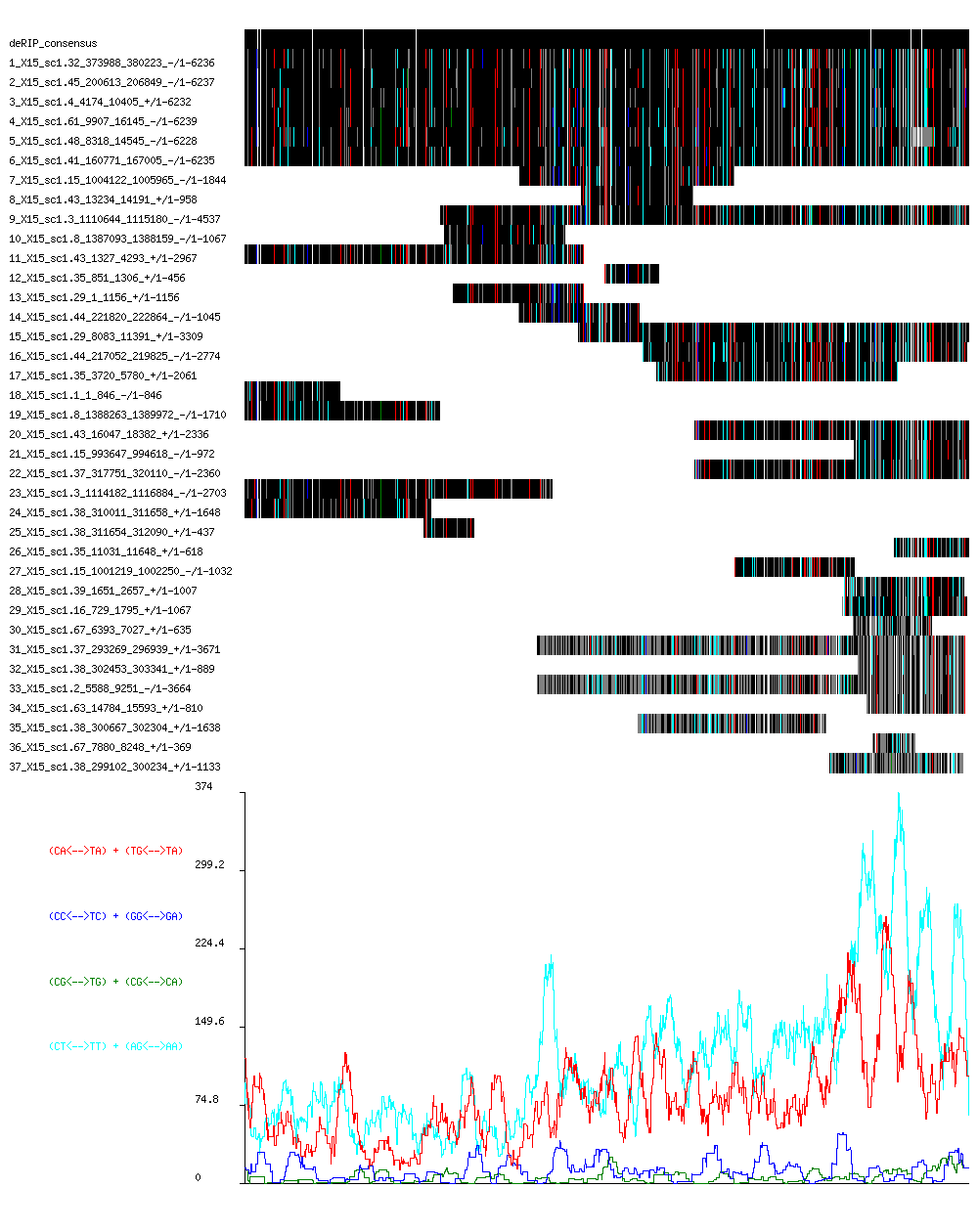

Supplement: Additional file 6 — List of predicted functional RecQ helicases in the S. nodorum genome. [file 1471-2164-11-655-S6.ZIP › derip/x15_aln_derip_2009_8_19_15_44_48_input.gif]

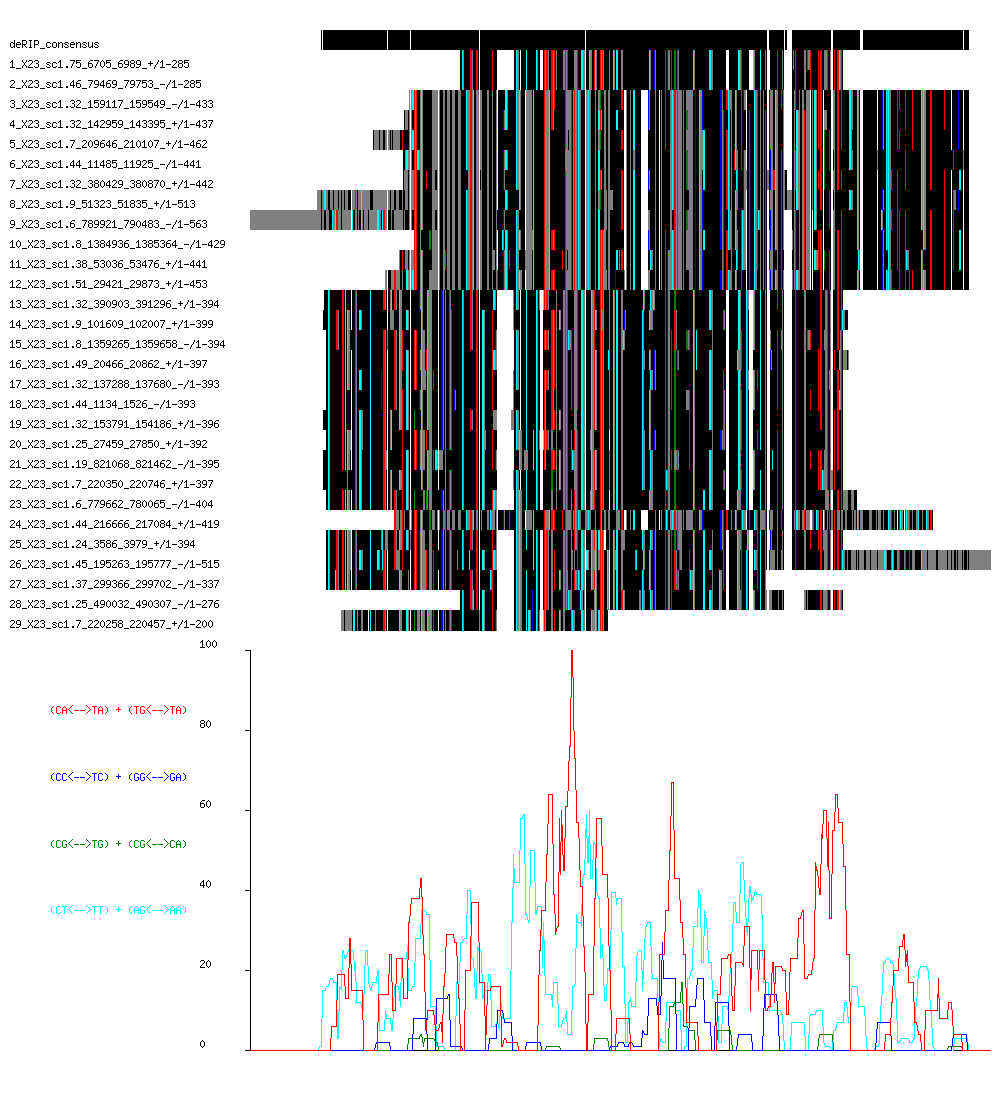

Supplement: Additional file 6 — List of predicted functional RecQ helicases in the S. nodorum genome. [file 1471-2164-11-655-S6.ZIP › derip/x23_aln_derip_2009_8_19_15_45_22_input.gif]

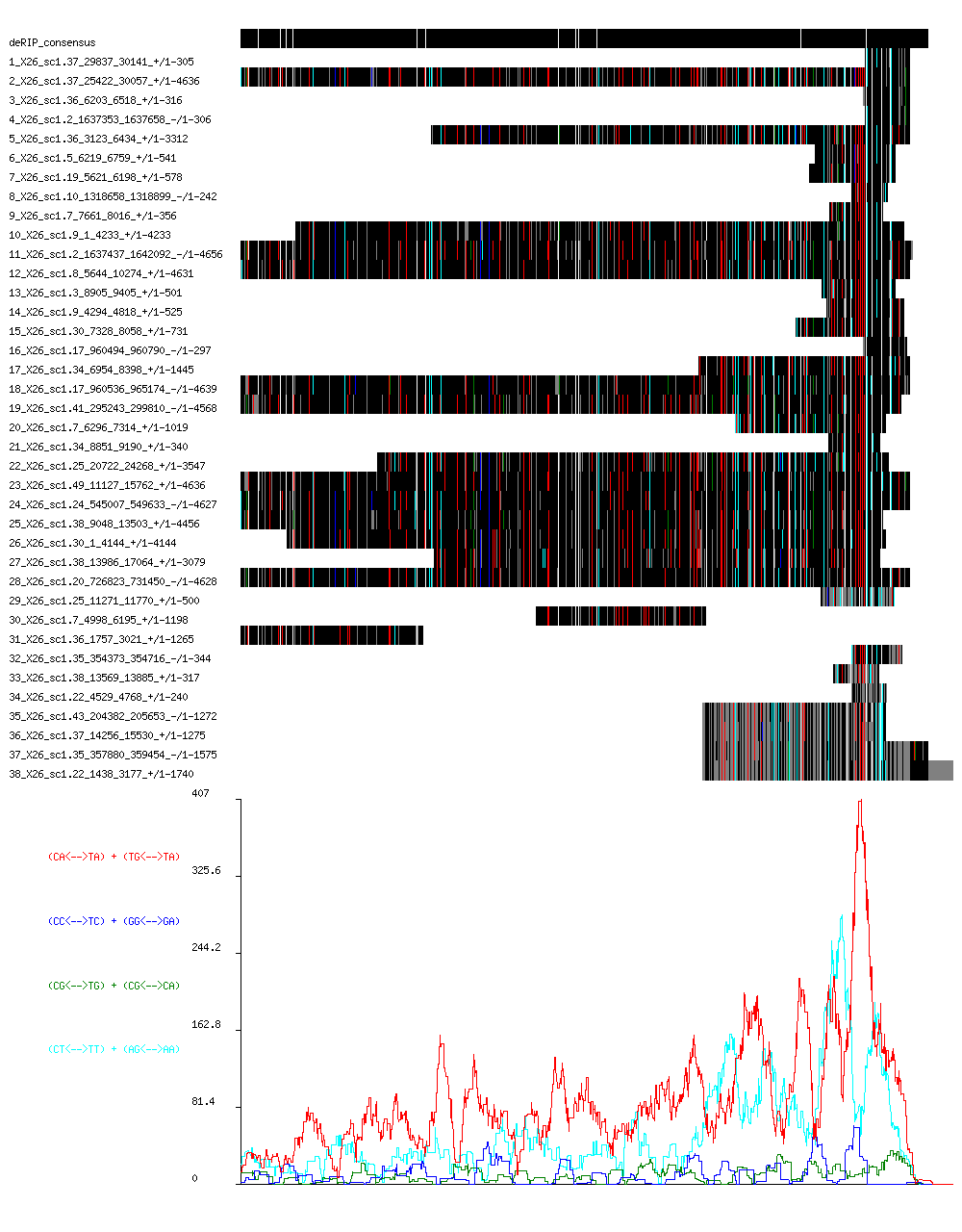

Supplement: Additional file 6 — List of predicted functional RecQ helicases in the S. nodorum genome. [file 1471-2164-11-655-S6.ZIP › derip/x26_aln_derip_2009_8_19_15_45_24_input.gif]

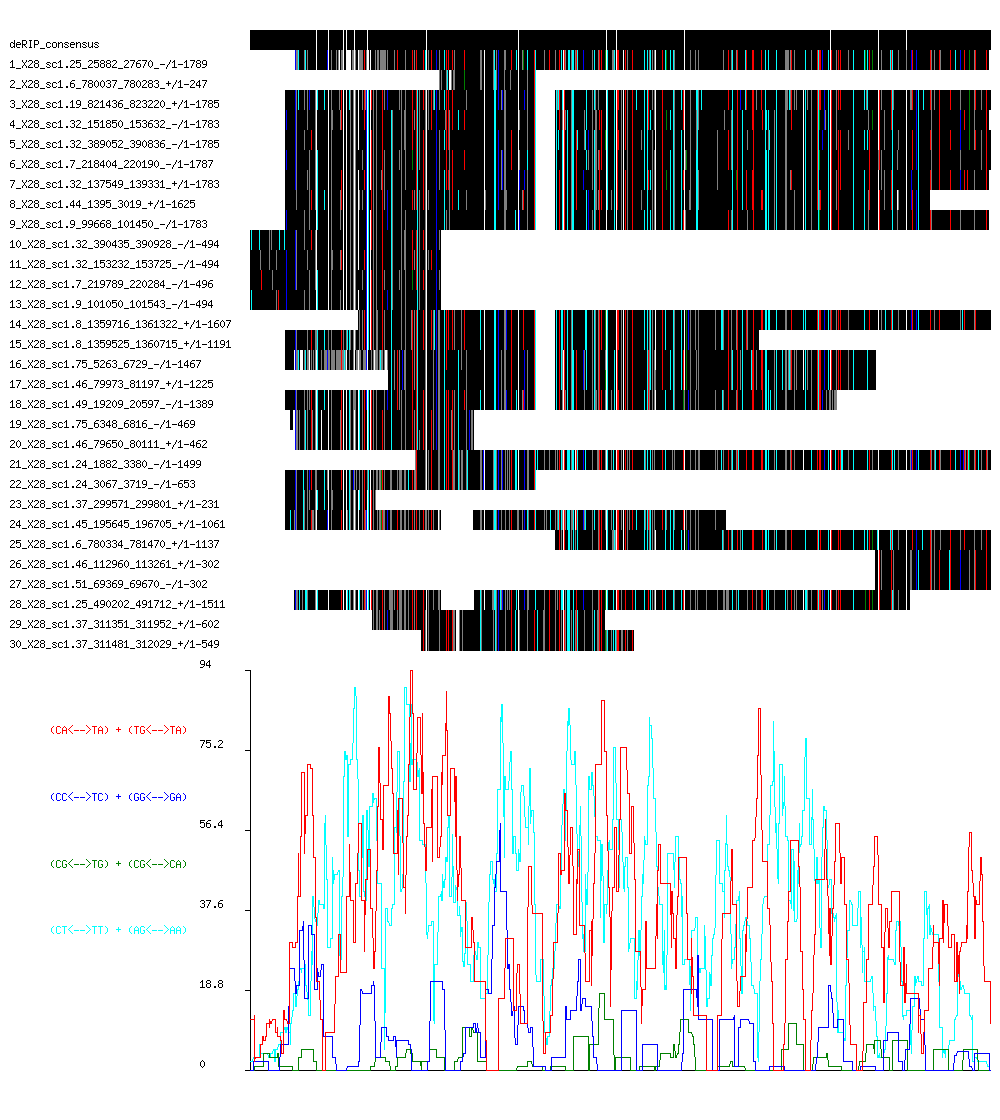

Supplement: Additional file 6 — List of predicted functional RecQ helicases in the S. nodorum genome. [file 1471-2164-11-655-S6.ZIP › derip/x28_aln_derip_2009_8_19_15_45_51_input.gif]

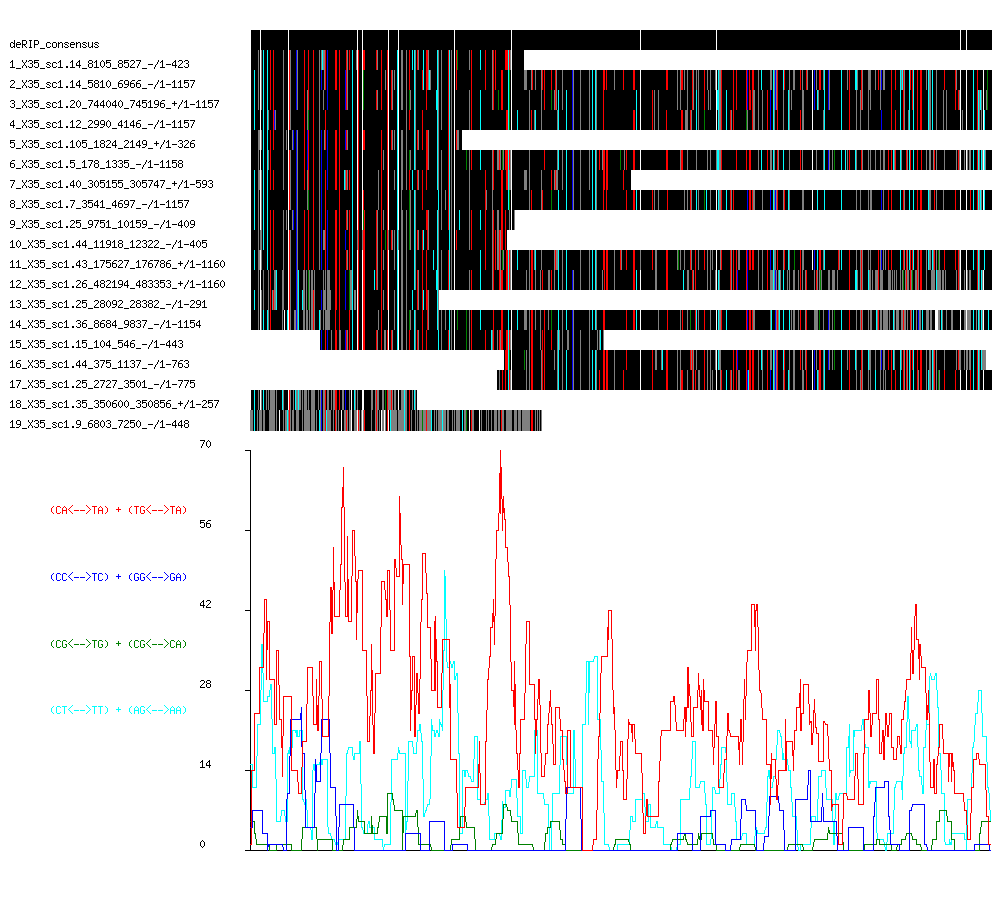

Supplement: Additional file 6 — List of predicted functional RecQ helicases in the S. nodorum genome. [file 1471-2164-11-655-S6.ZIP › derip/x35_aln_derip_2009_8_19_15_45_59_input.gif]

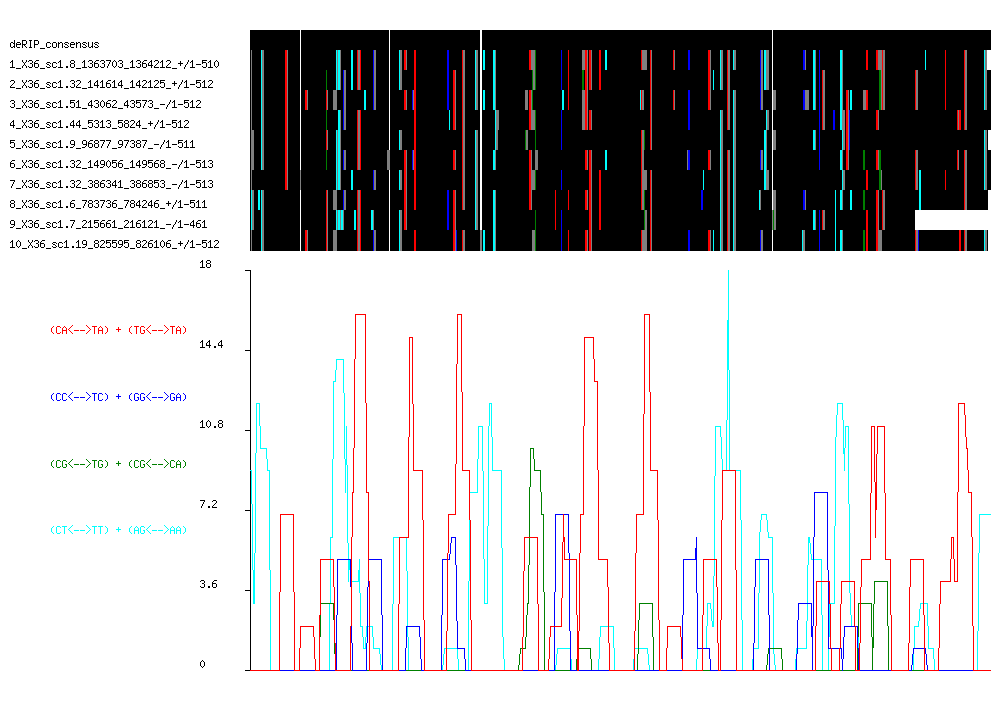

Supplement: Additional file 6 — List of predicted functional RecQ helicases in the S. nodorum genome. [file 1471-2164-11-655-S6.ZIP › derip/x36_aln_derip_2009_8_19_15_46_02_input.gif]

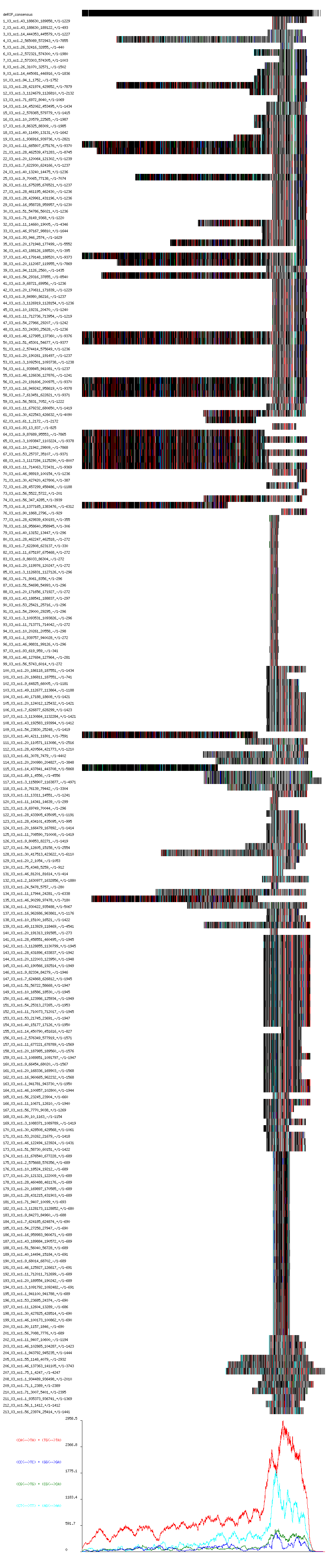

Supplement: Additional file 6 — List of predicted functional RecQ helicases in the S. nodorum genome. [file 1471-2164-11-655-S6.ZIP › derip/x3_aln_derip_2009_8_19_15_46_03_input.gif]

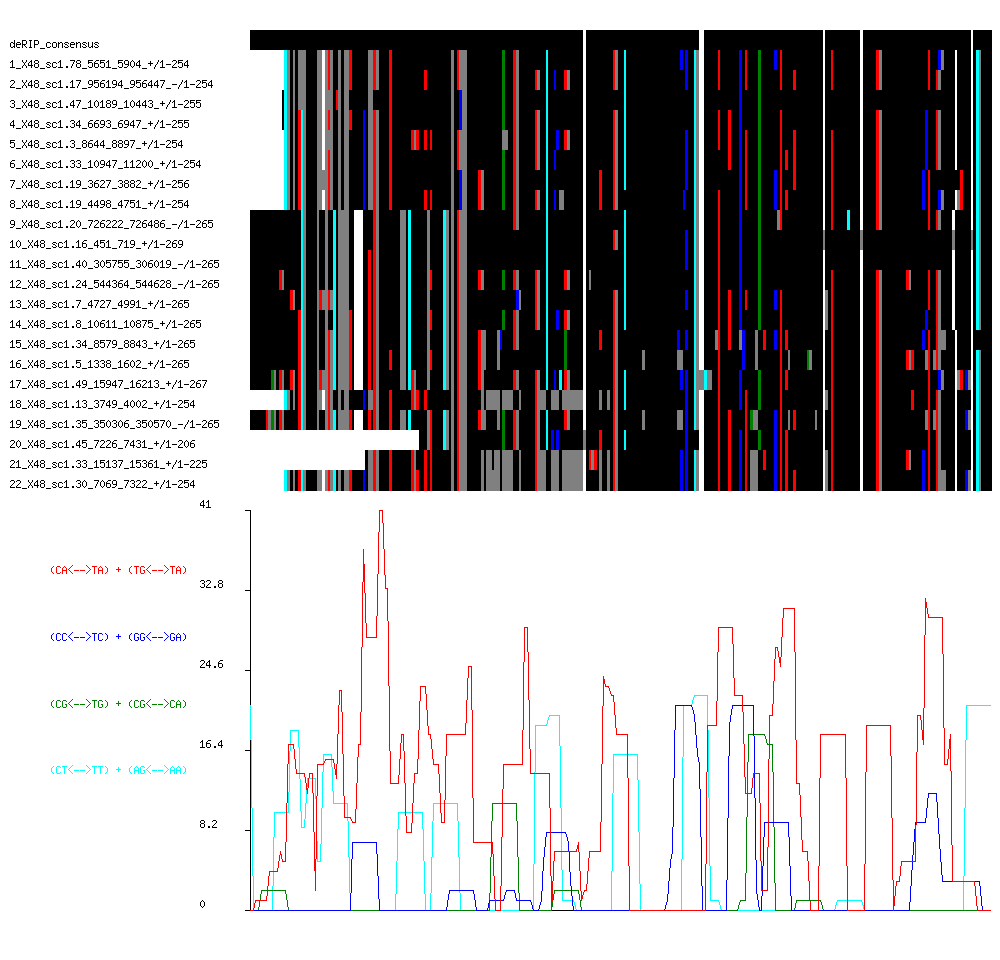

Supplement: Additional file 6 — List of predicted functional RecQ helicases in the S. nodorum genome. [file 1471-2164-11-655-S6.ZIP › derip/x48_aln_derip_2009_8_19_15_51_05_input.gif]

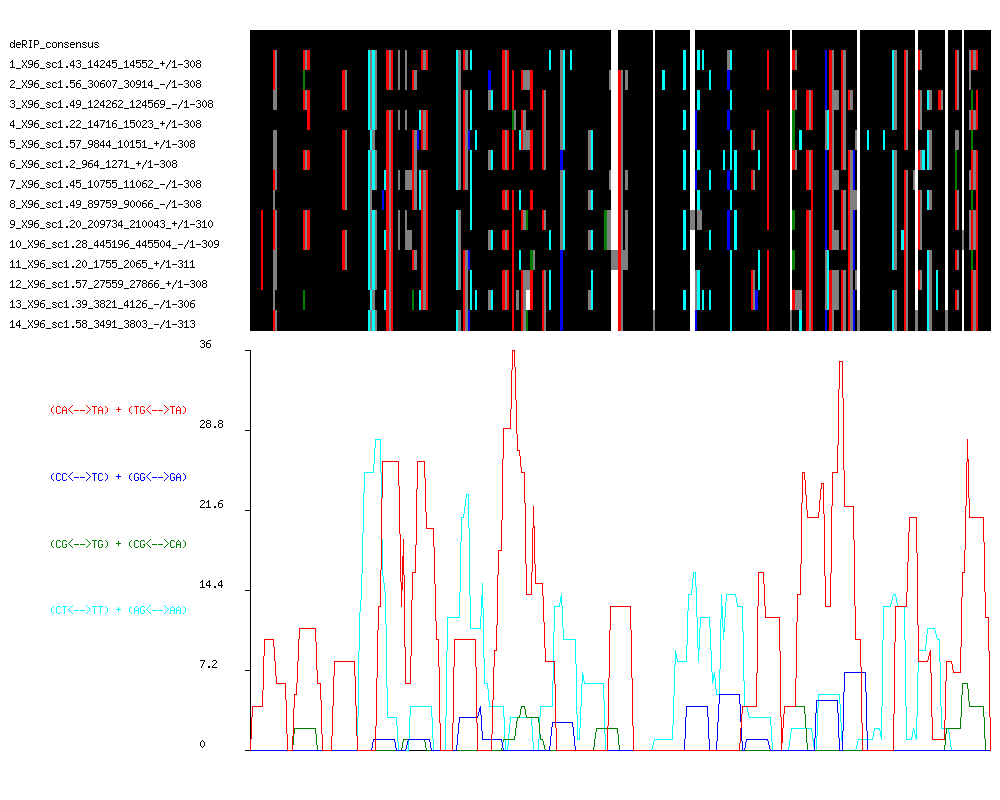

Supplement: Additional file 6 — List of predicted functional RecQ helicases in the S. nodorum genome. [file 1471-2164-11-655-S6.ZIP › derip/x96_aln_derip_2009_8_19_15_51_06_input.gif]

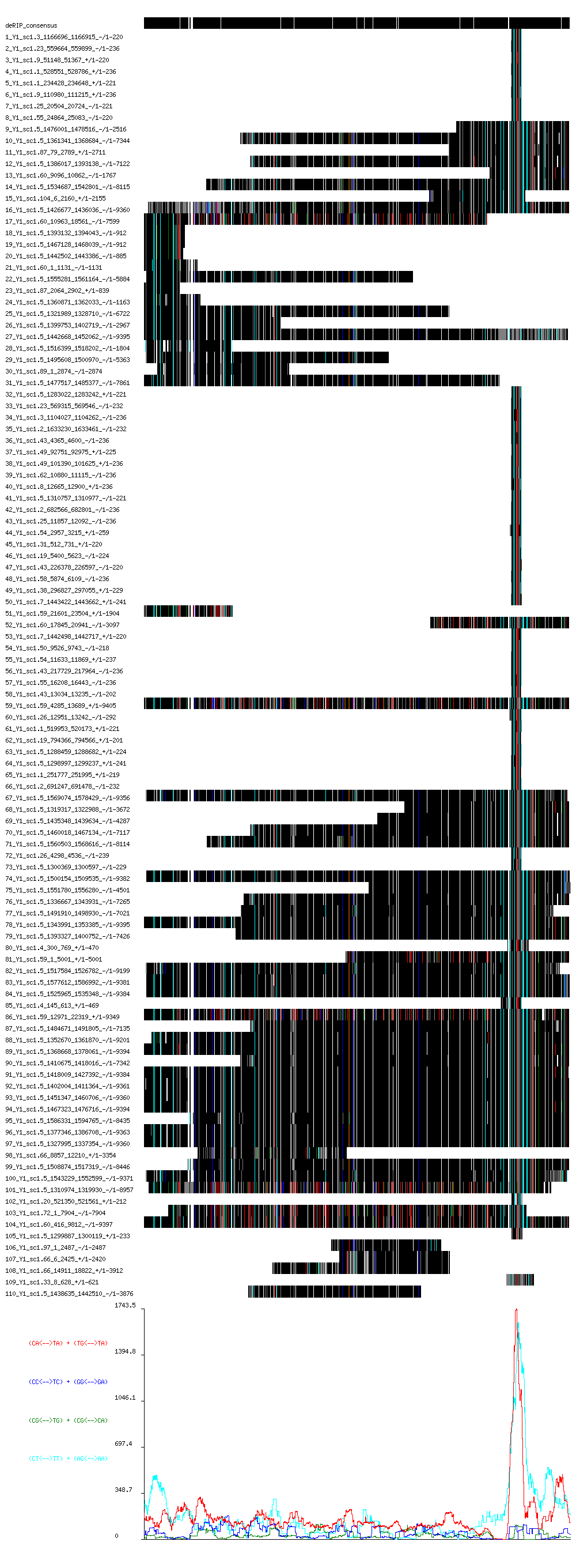

Supplement: Additional file 6 — List of predicted functional RecQ helicases in the S. nodorum genome. [file 1471-2164-11-655-S6.ZIP › derip/y1_aln_derip_2009_8_19_15_51_07_input.gif]

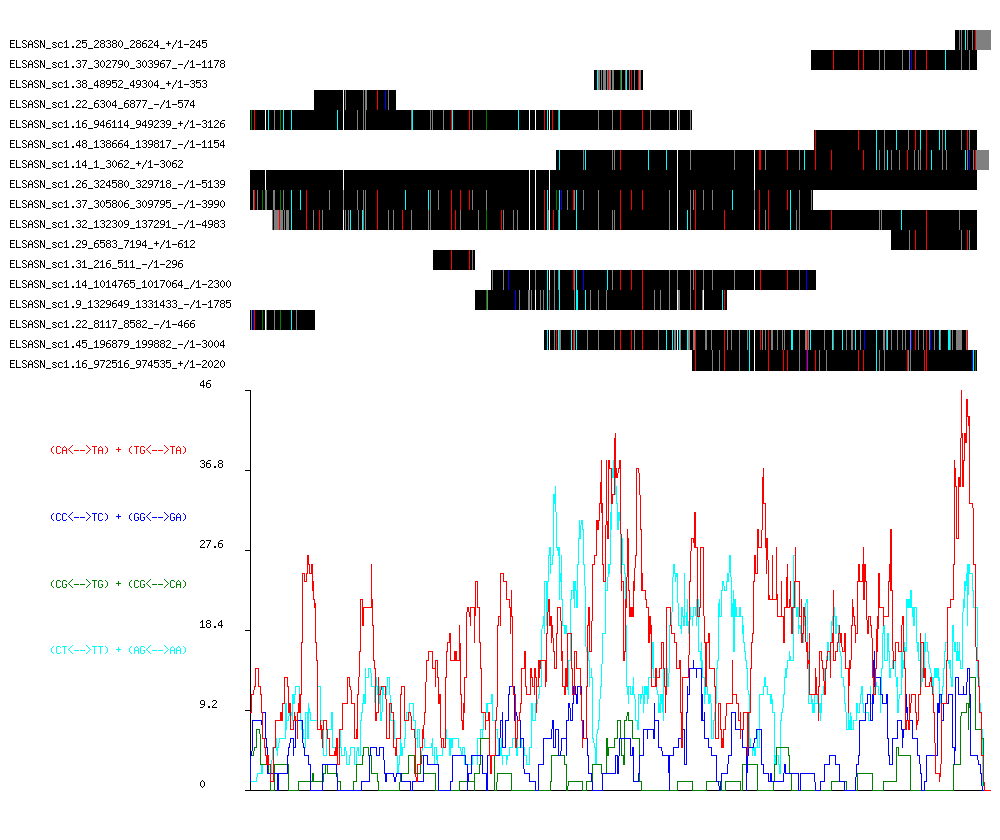

Supplement: Additional file 6 — List of predicted functional RecQ helicases in the S. nodorum genome. [file 1471-2164-11-655-S6.ZIP › highestgc/elsa_aln_2009_9_24_11_13_22_ELSASN_sc1.26_324580_329718_-_1-5139.gif]

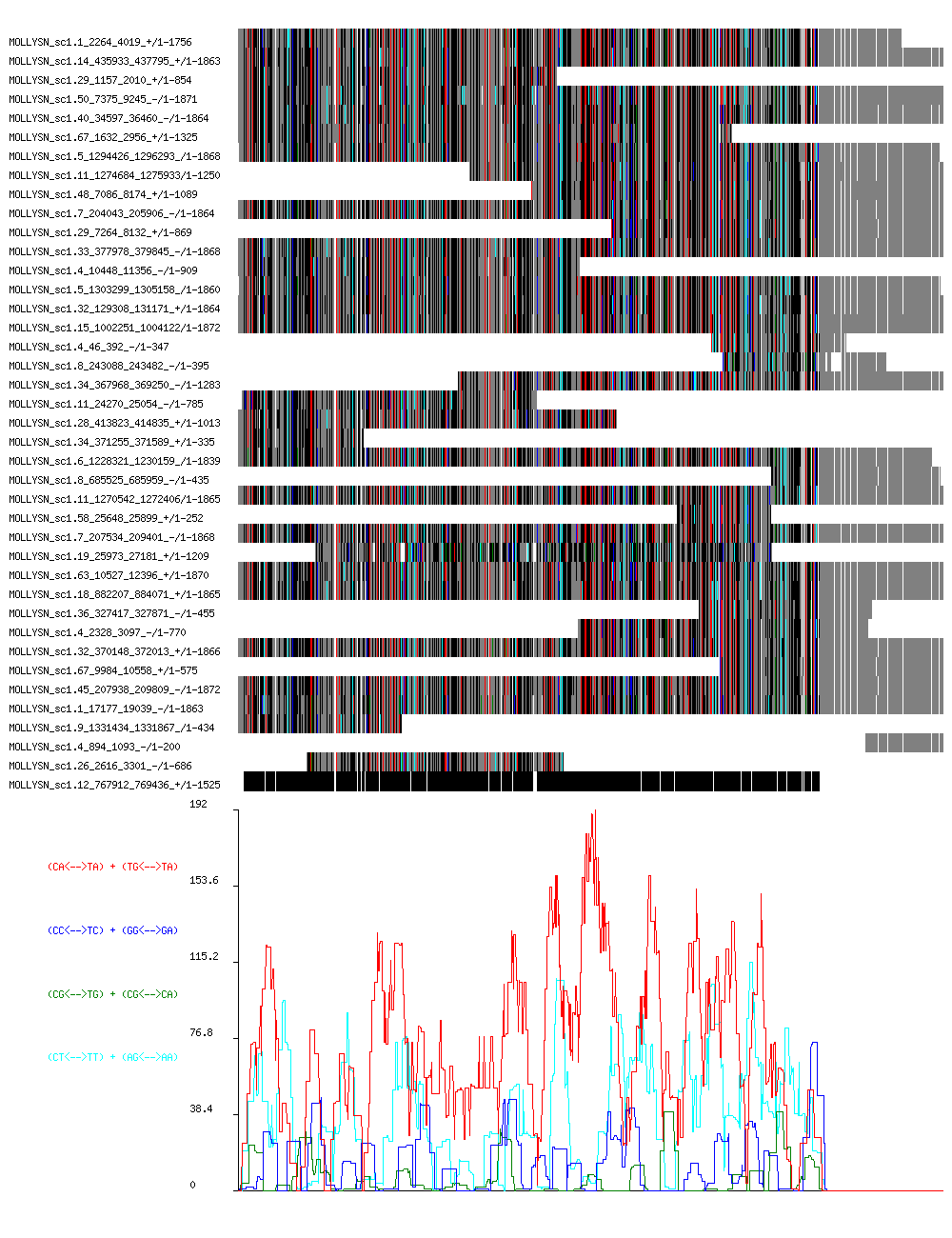

Supplement: Additional file 6 — List of predicted functional RecQ helicases in the S. nodorum genome. [file 1471-2164-11-655-S6.ZIP › highestgc/molly_aln_2009_9_24_11_13_36_MOLLYSN_sc1.12_767912_769436_+_1-1525.gif]

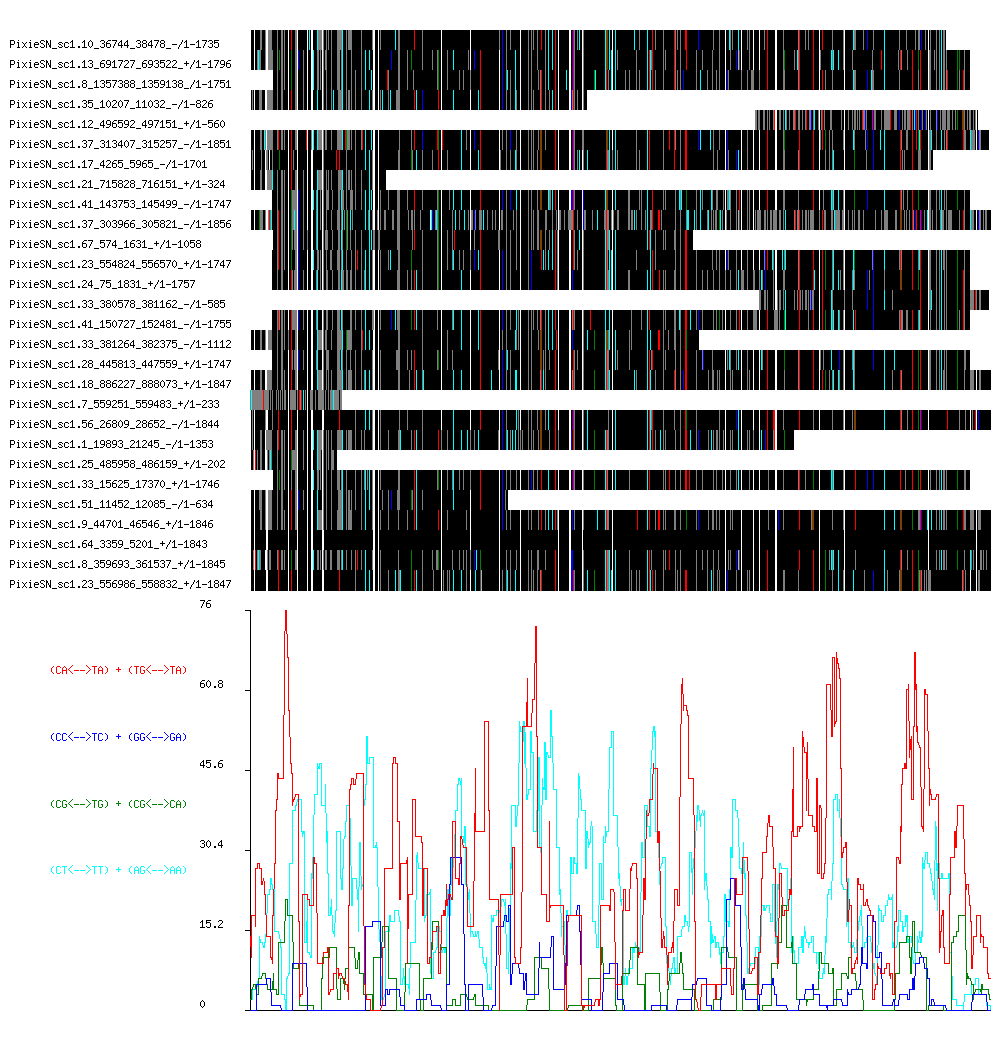

Supplement: Additional file 6 — List of predicted functional RecQ helicases in the S. nodorum genome. [file 1471-2164-11-655-S6.ZIP › highestgc/pixie_aln_2009_9_24_11_13_45_PixieSN_sc1.64_3359_5201_+_1-1843.gif]

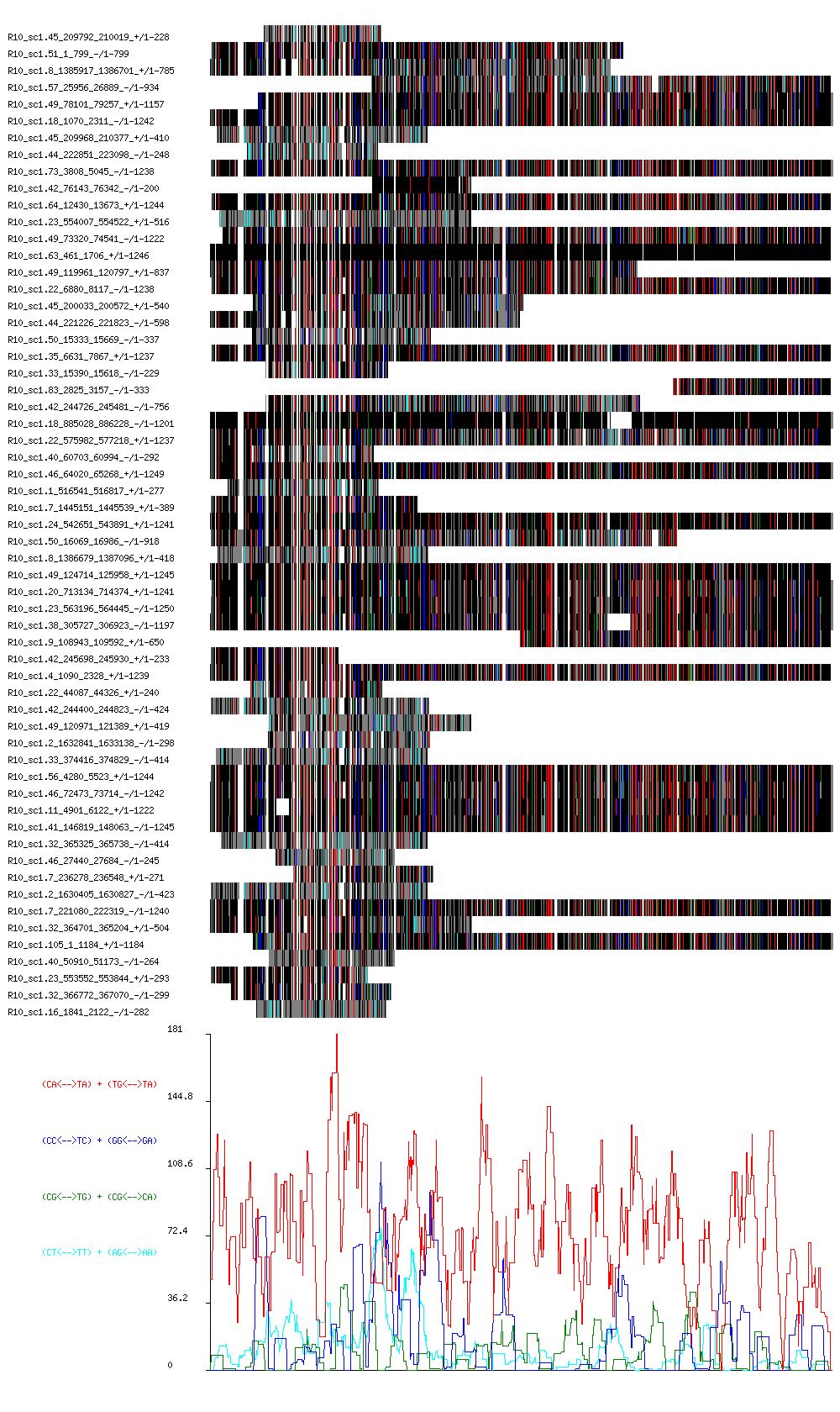

Supplement: Additional file 6 — List of predicted functional RecQ helicases in the S. nodorum genome. [file 1471-2164-11-655-S6.ZIP › highestgc/r10_aln_2009_9_24_11_13_52_R10_sc1.63_461_1706_+_1-1246.gif]

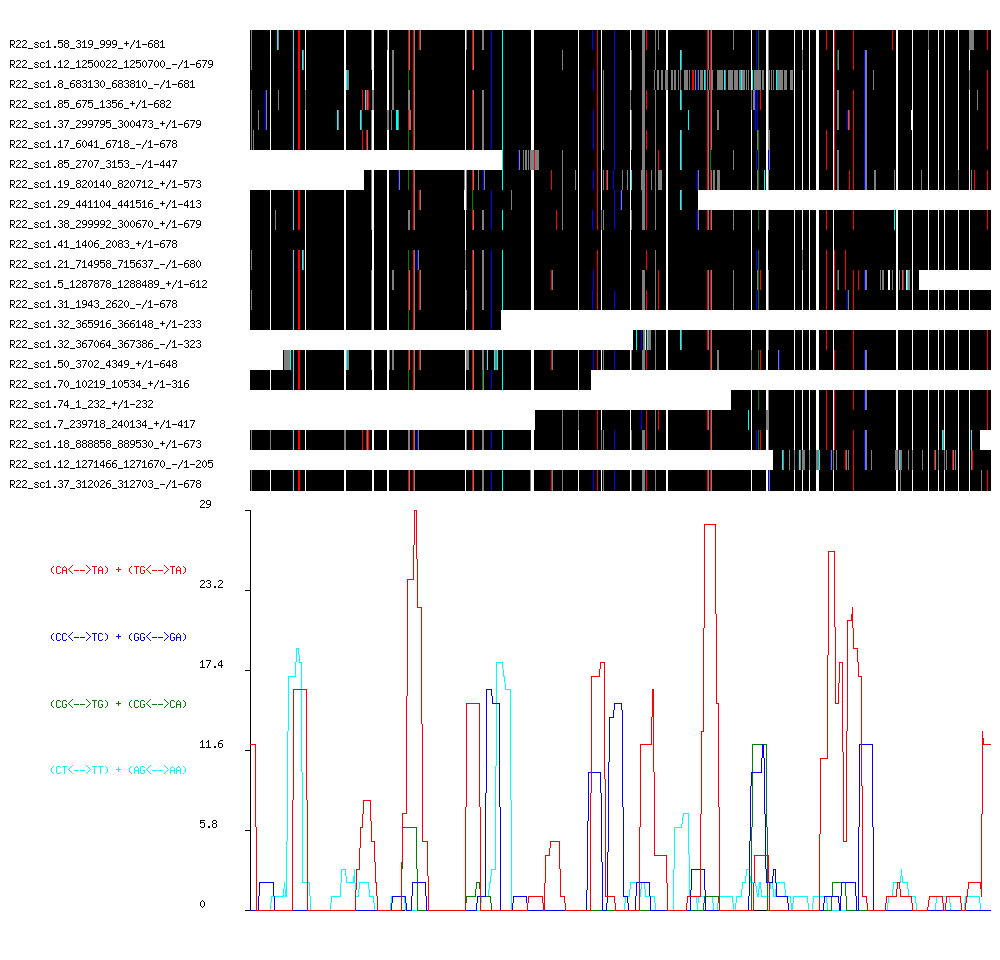

Supplement: Additional file 6 — List of predicted functional RecQ helicases in the S. nodorum genome. [file 1471-2164-11-655-S6.ZIP › highestgc/r22_aln_2009_9_24_11_14_02_R22_sc1.41_1406_2083_+_1-678.gif]

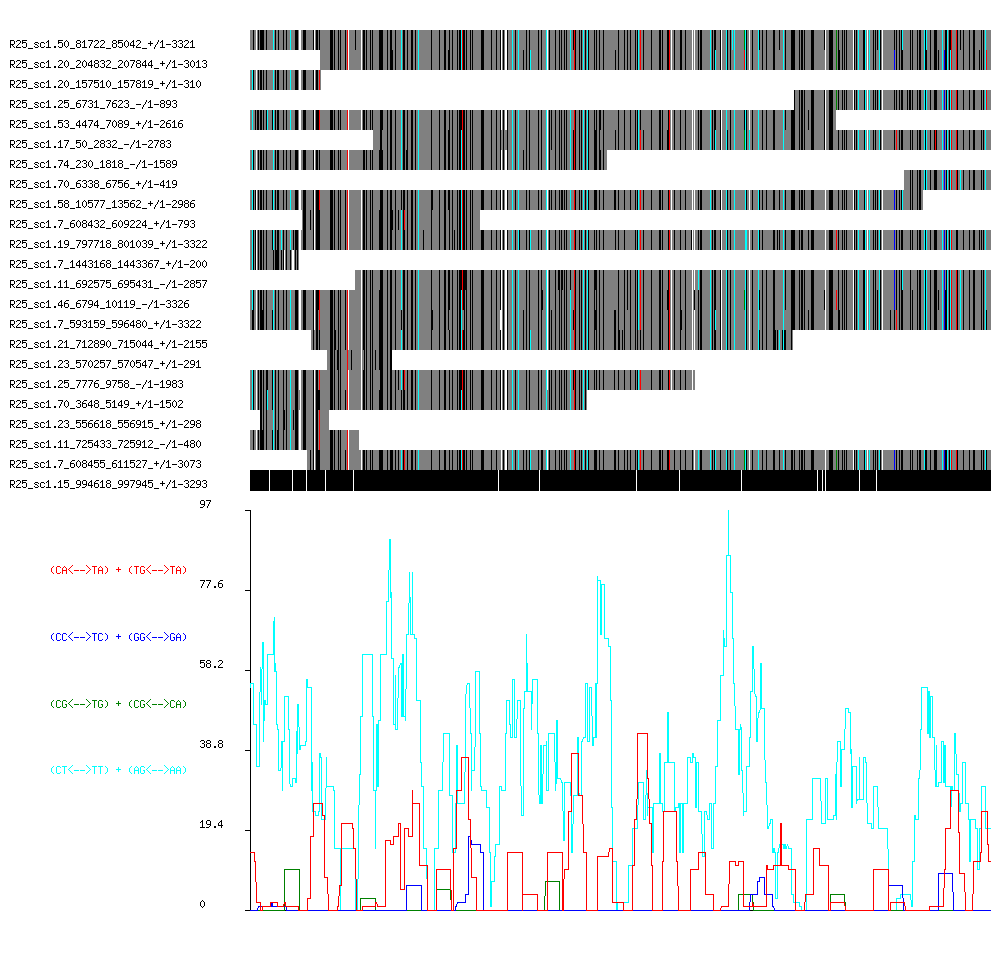

Supplement: Additional file 6 — List of predicted functional RecQ helicases in the S. nodorum genome. [file 1471-2164-11-655-S6.ZIP › highestgc/r25_aln_2009_9_24_11_14_05_R25_sc1.15_994618_997945_+_1-3293.gif]

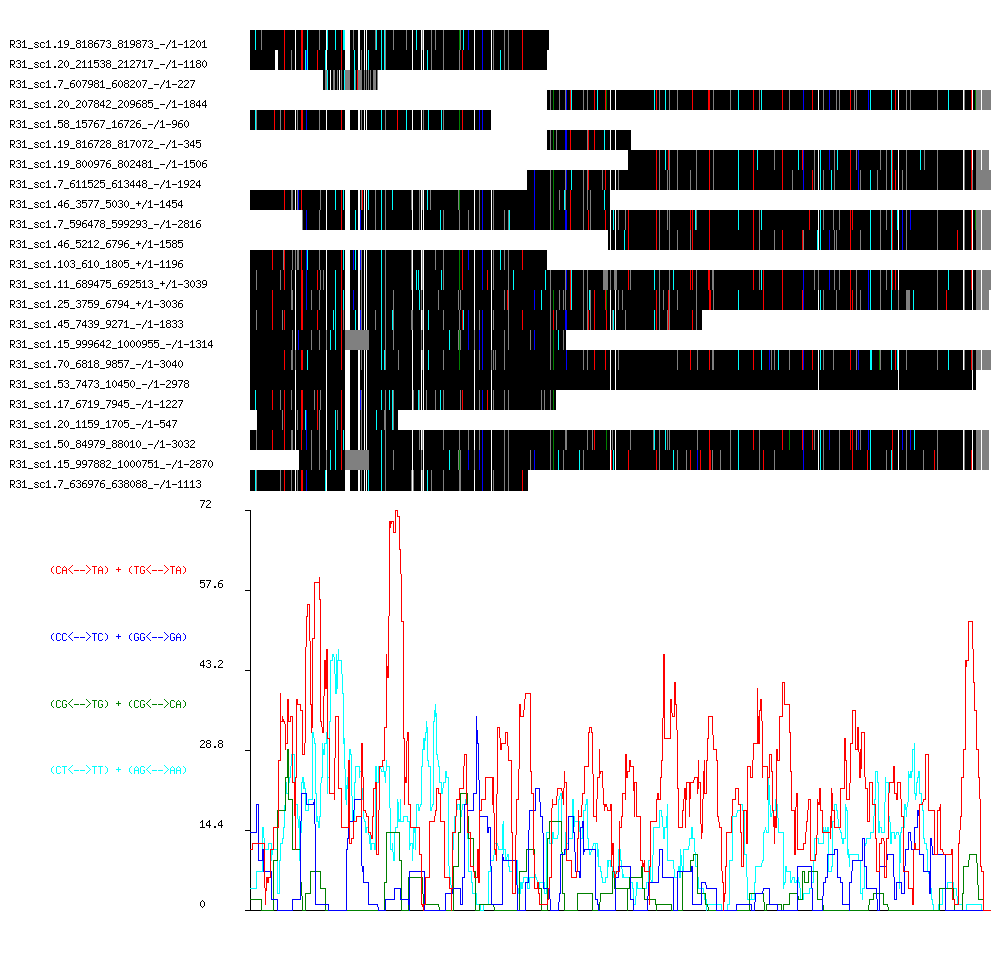

Supplement: Additional file 6 — List of predicted functional RecQ helicases in the S. nodorum genome. [file 1471-2164-11-655-S6.ZIP › highestgc/r31_aln_2009_9_24_11_14_15_R31_sc1.53_7473_10450_-_1-2978.gif]

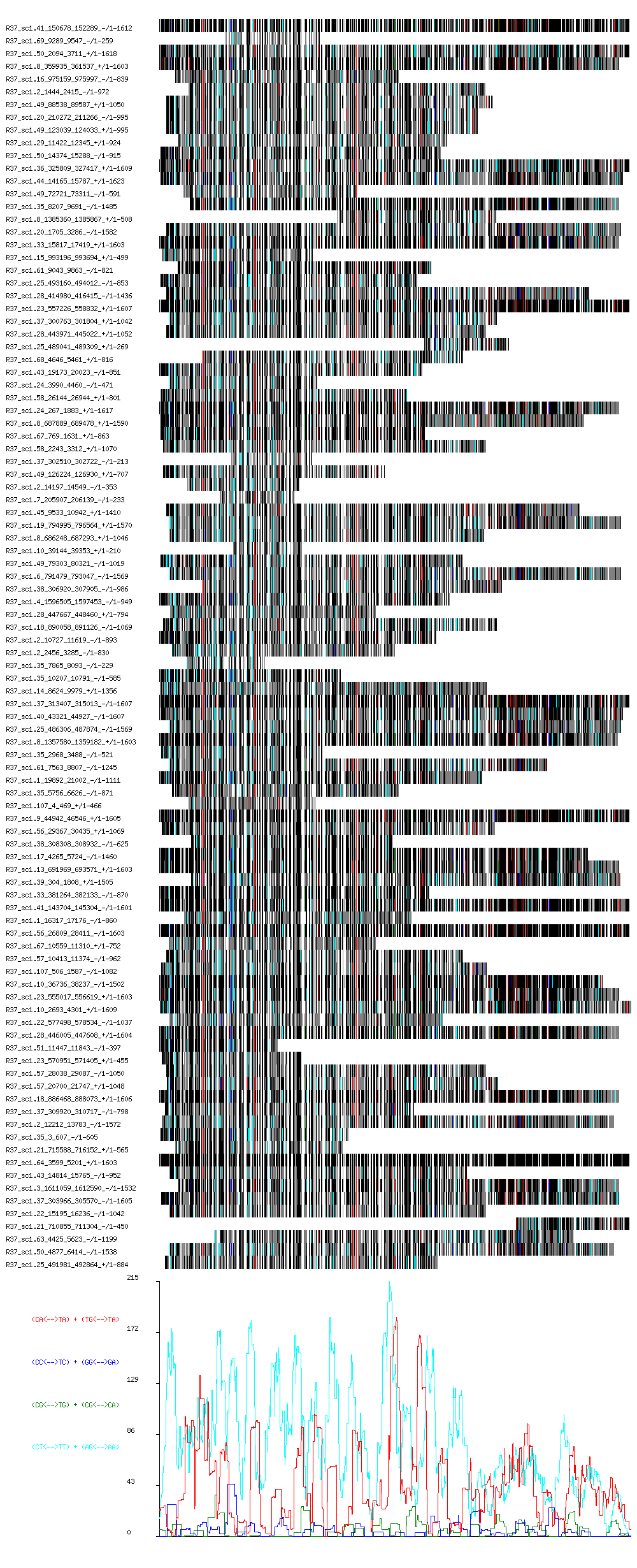

Supplement: Additional file 6 — List of predicted functional RecQ helicases in the S. nodorum genome. [file 1471-2164-11-655-S6.ZIP › highestgc/r37_aln_2009_9_24_11_14_24_R37_sc1.64_3599_5201_+_1-1603.gif]

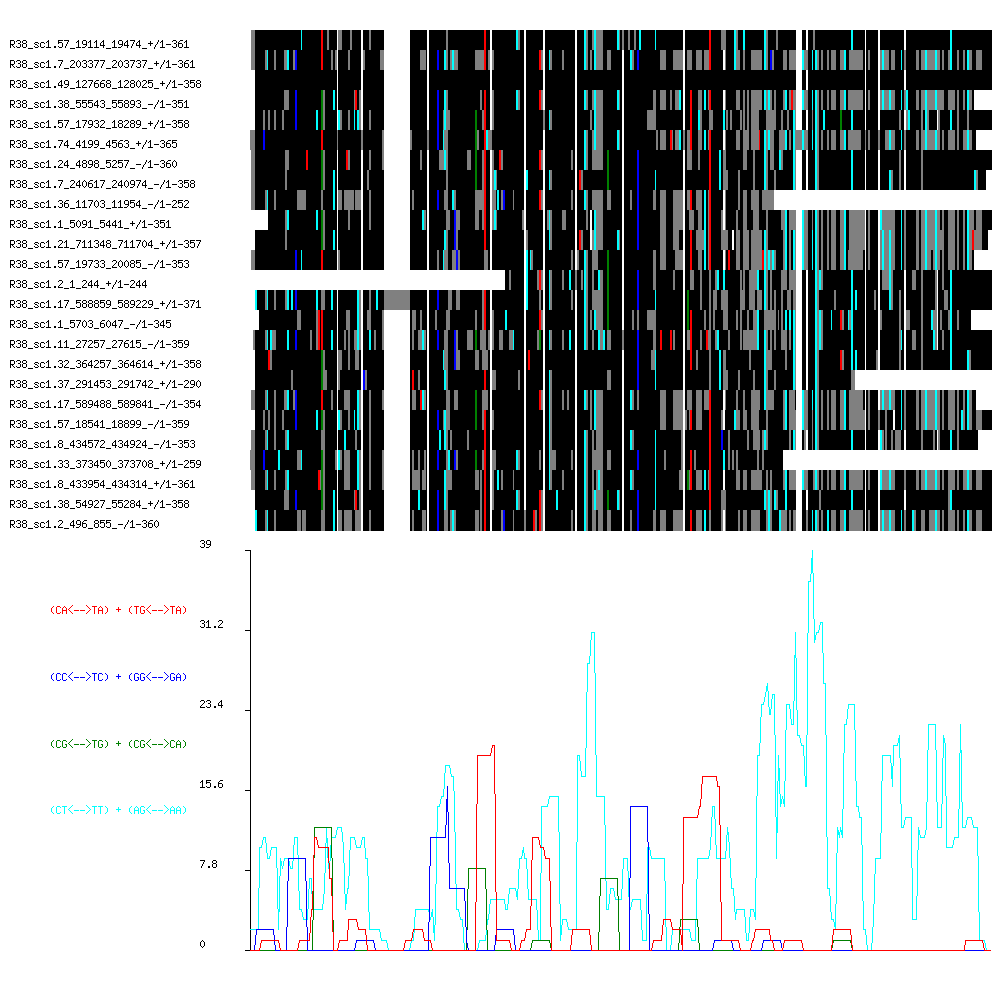

Supplement: Additional file 6 — List of predicted functional RecQ helicases in the S. nodorum genome. [file 1471-2164-11-655-S6.ZIP › highestgc/r38_aln_2009_9_24_11_14_49_R38_sc1.49_127668_128025_+_1-358.gif]

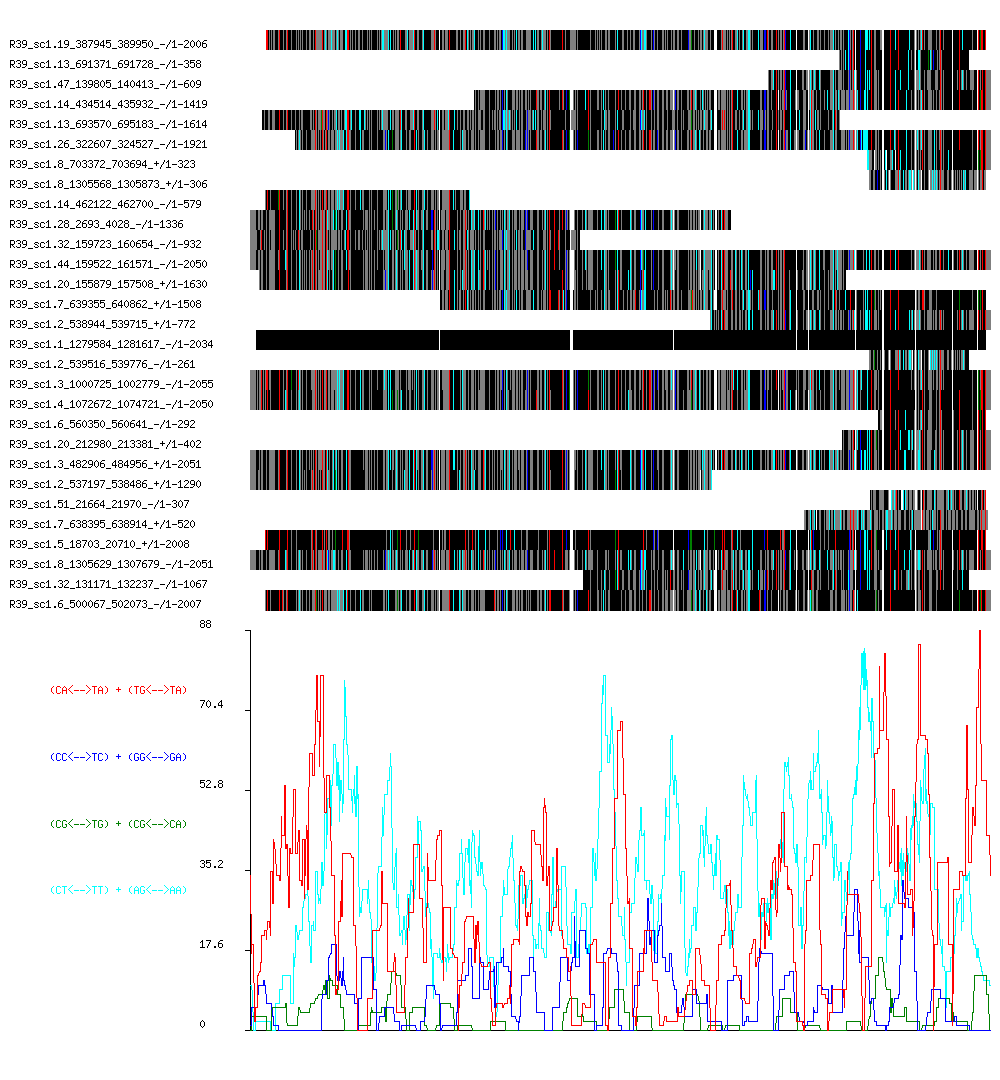

Supplement: Additional file 6 — List of predicted functional RecQ helicases in the S. nodorum genome. [file 1471-2164-11-655-S6.ZIP › highestgc/r39_aln_2009_9_24_11_14_51_R39_sc1.1_1279584_1281617_-_1-2034.gif]

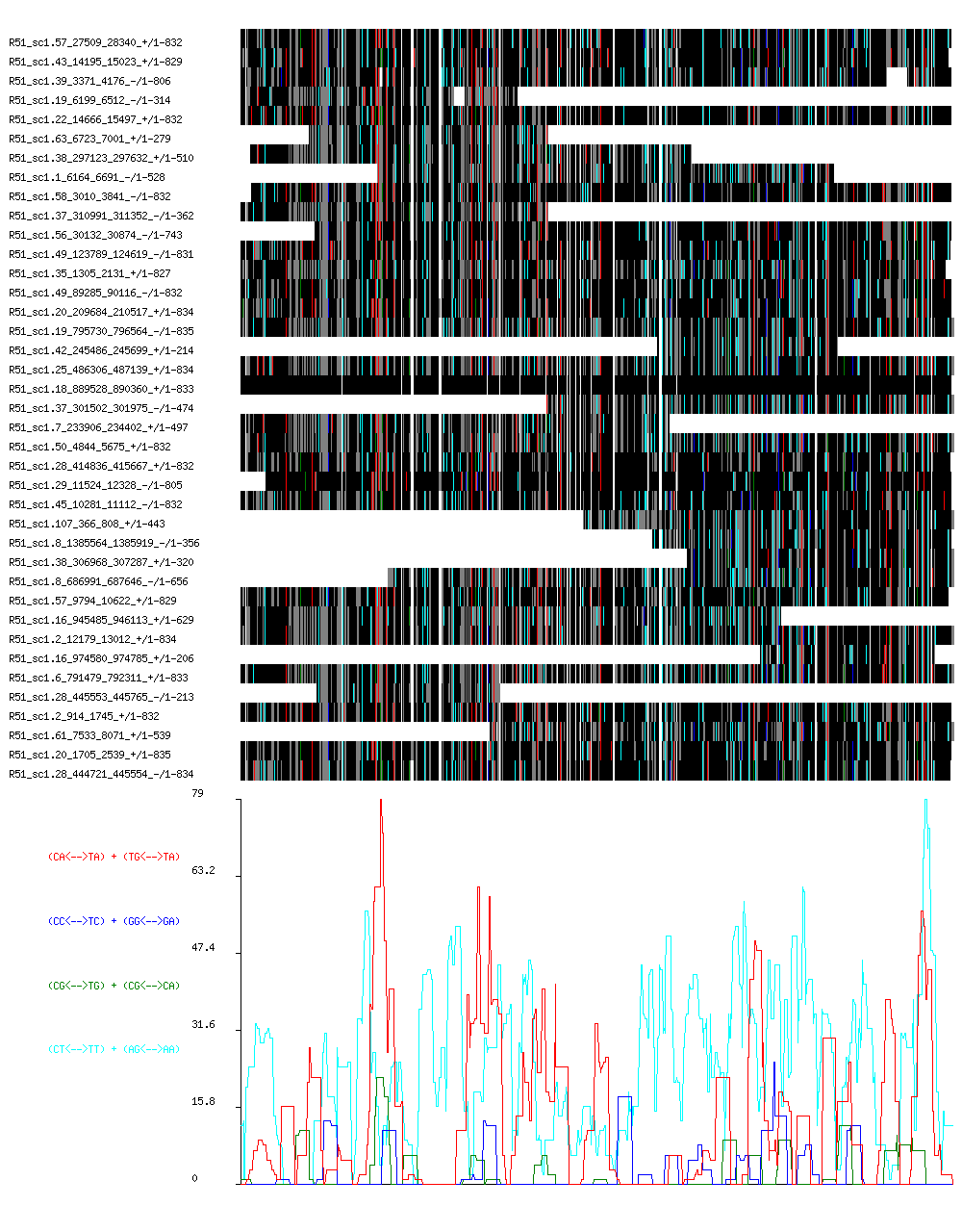

Supplement: Additional file 6 — List of predicted functional RecQ helicases in the S. nodorum genome. [file 1471-2164-11-655-S6.ZIP › highestgc/r51_aln_2009_9_24_11_14_59_R51_sc1.18_889528_890360_+_1-833.gif]

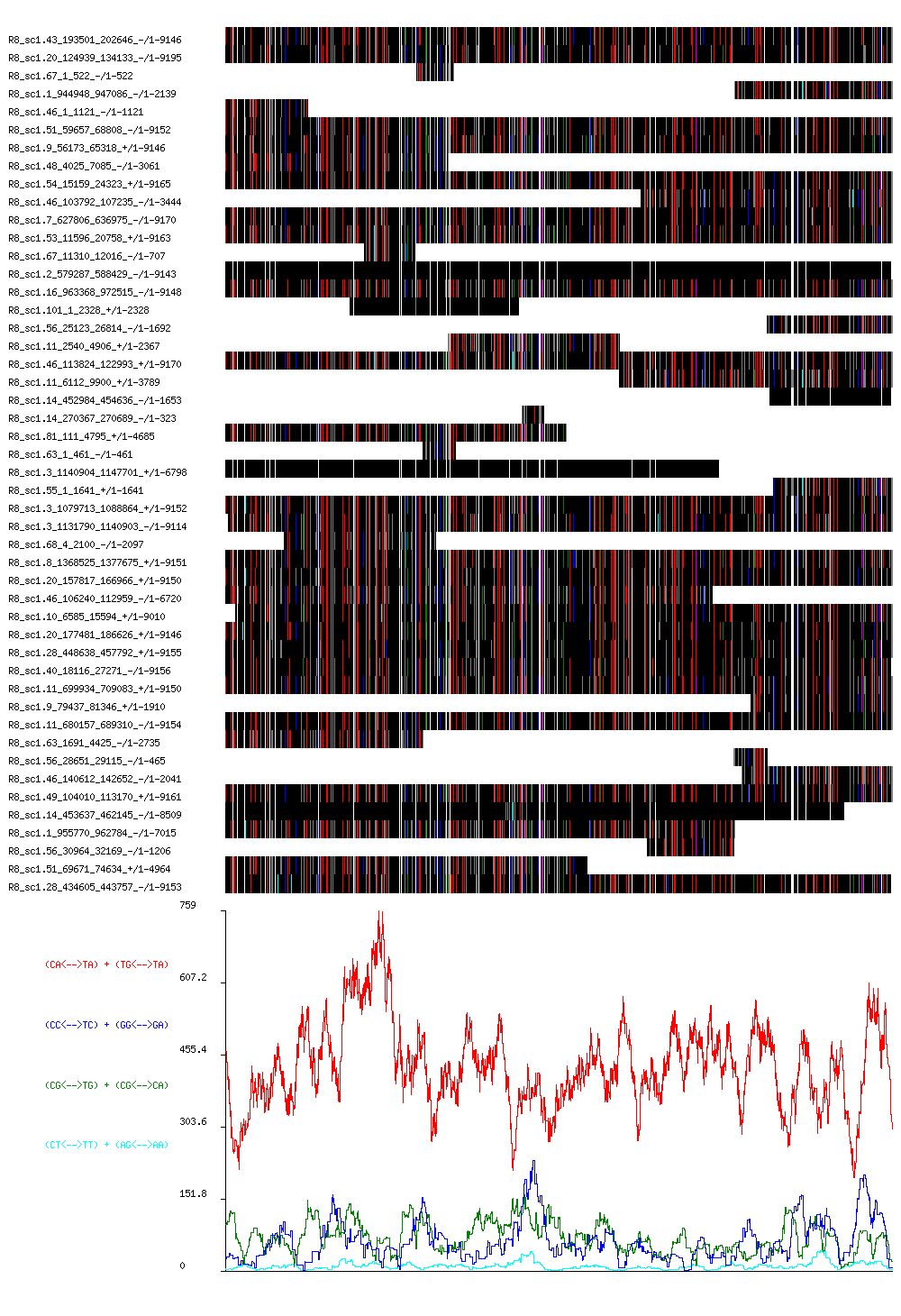

Supplement: Additional file 6 — List of predicted functional RecQ helicases in the S. nodorum genome. [file 1471-2164-11-655-S6.ZIP › highestgc/r8_aln_2009_9_24_11_15_04_R8_sc1.2_579287_588429_-_1-9143.gif]

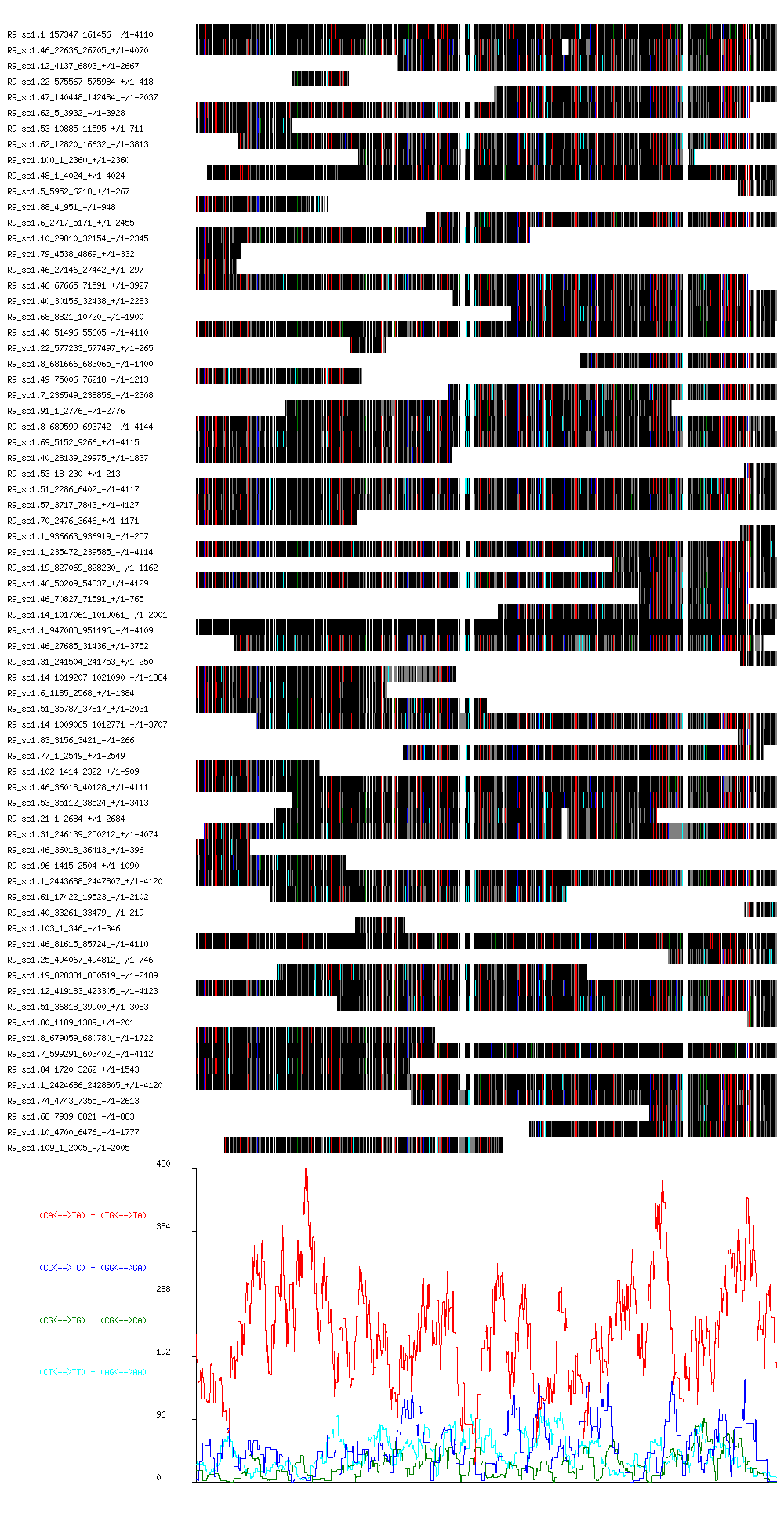

Supplement: Additional file 6 — List of predicted functional RecQ helicases in the S. nodorum genome. [file 1471-2164-11-655-S6.ZIP › highestgc/r9_aln_2009_9_24_11_16_03_R9_sc1.1_947088_951196_-_1-4109.gif]

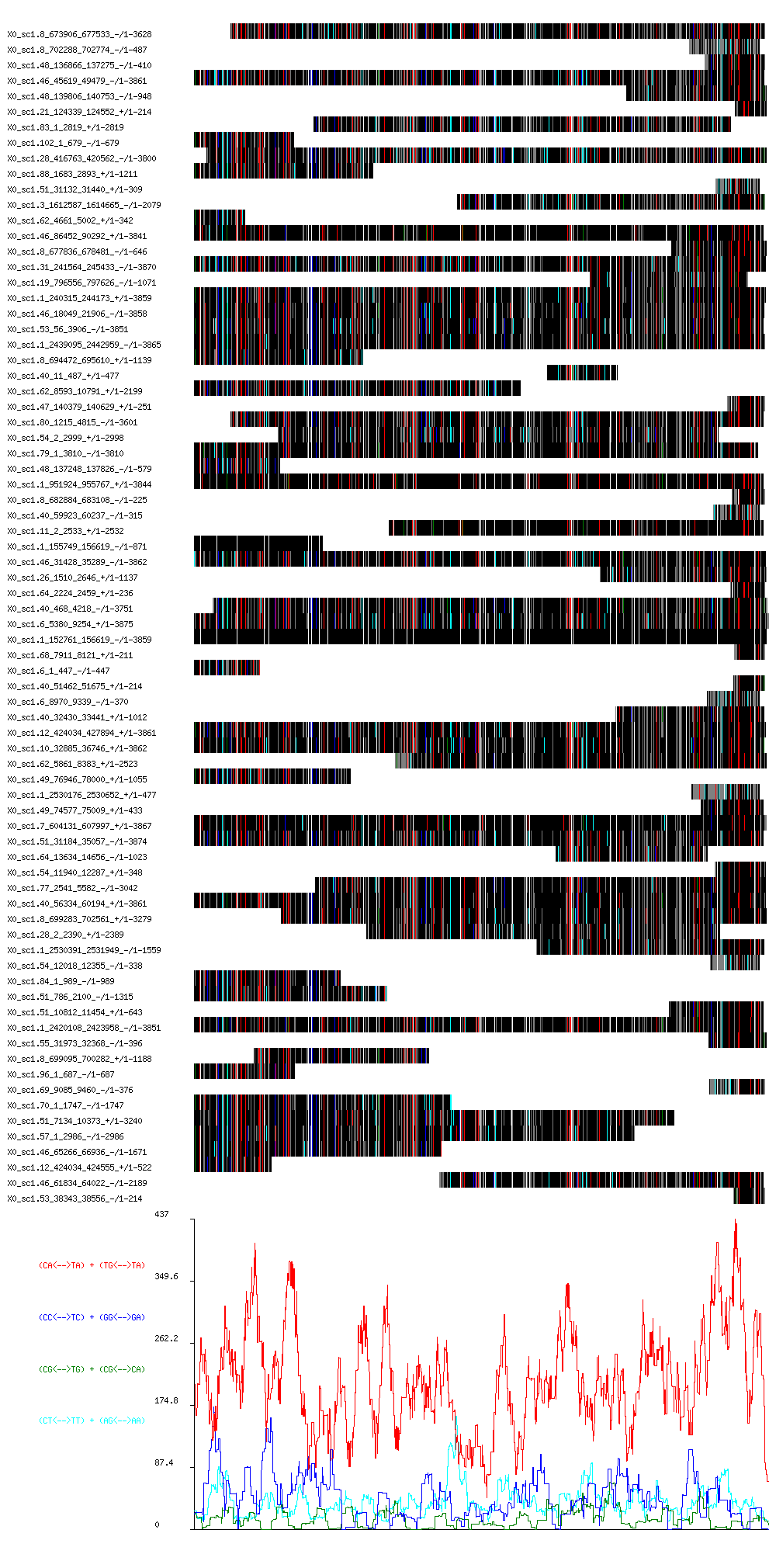

Supplement: Additional file 6 — List of predicted functional RecQ helicases in the S. nodorum genome. [file 1471-2164-11-655-S6.ZIP › highestgc/x0_aln_2009_9_24_11_16_42_X0_sc1.1_152761_156619_-_1-3859.gif]

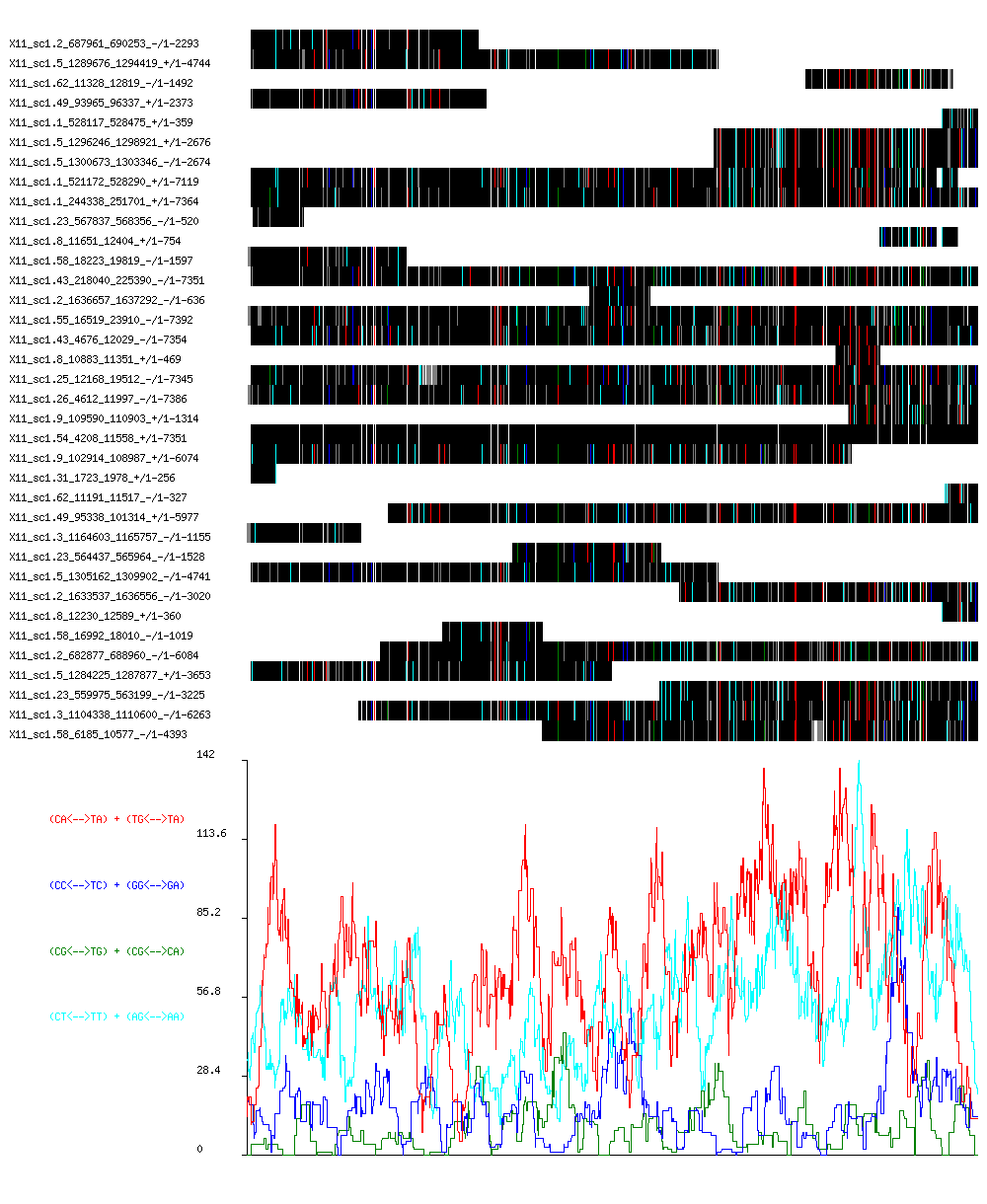

Supplement: Additional file 6 — List of predicted functional RecQ helicases in the S. nodorum genome. [file 1471-2164-11-655-S6.ZIP › highestgc/x11_aln_2009_9_24_11_17_19_X11_sc1.54_4208_11558_+_1-7351.gif]

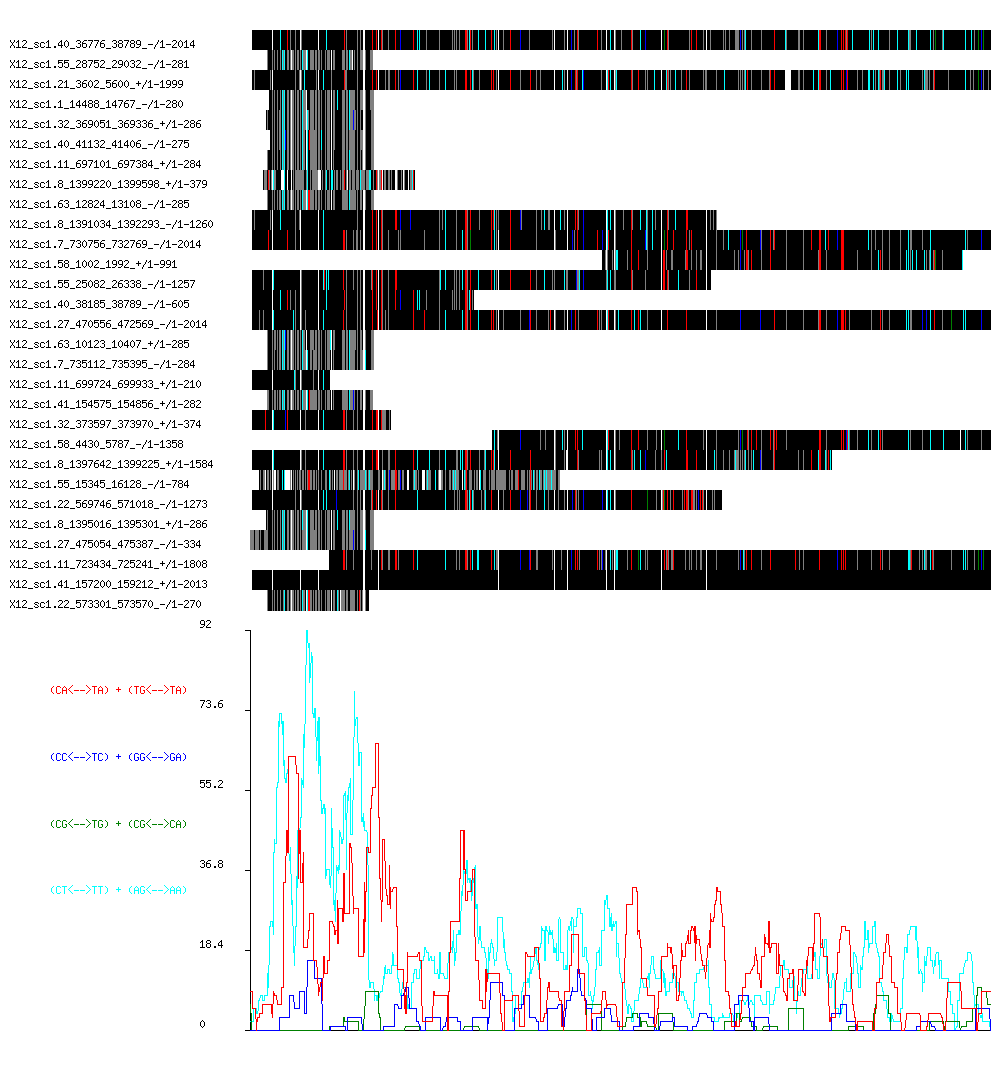

Supplement: Additional file 6 — List of predicted functional RecQ helicases in the S. nodorum genome. [file 1471-2164-11-655-S6.ZIP › highestgc/x12_aln_2009_9_24_11_17_56_X12_sc1.41_157200_159212_+_1-2013.gif]

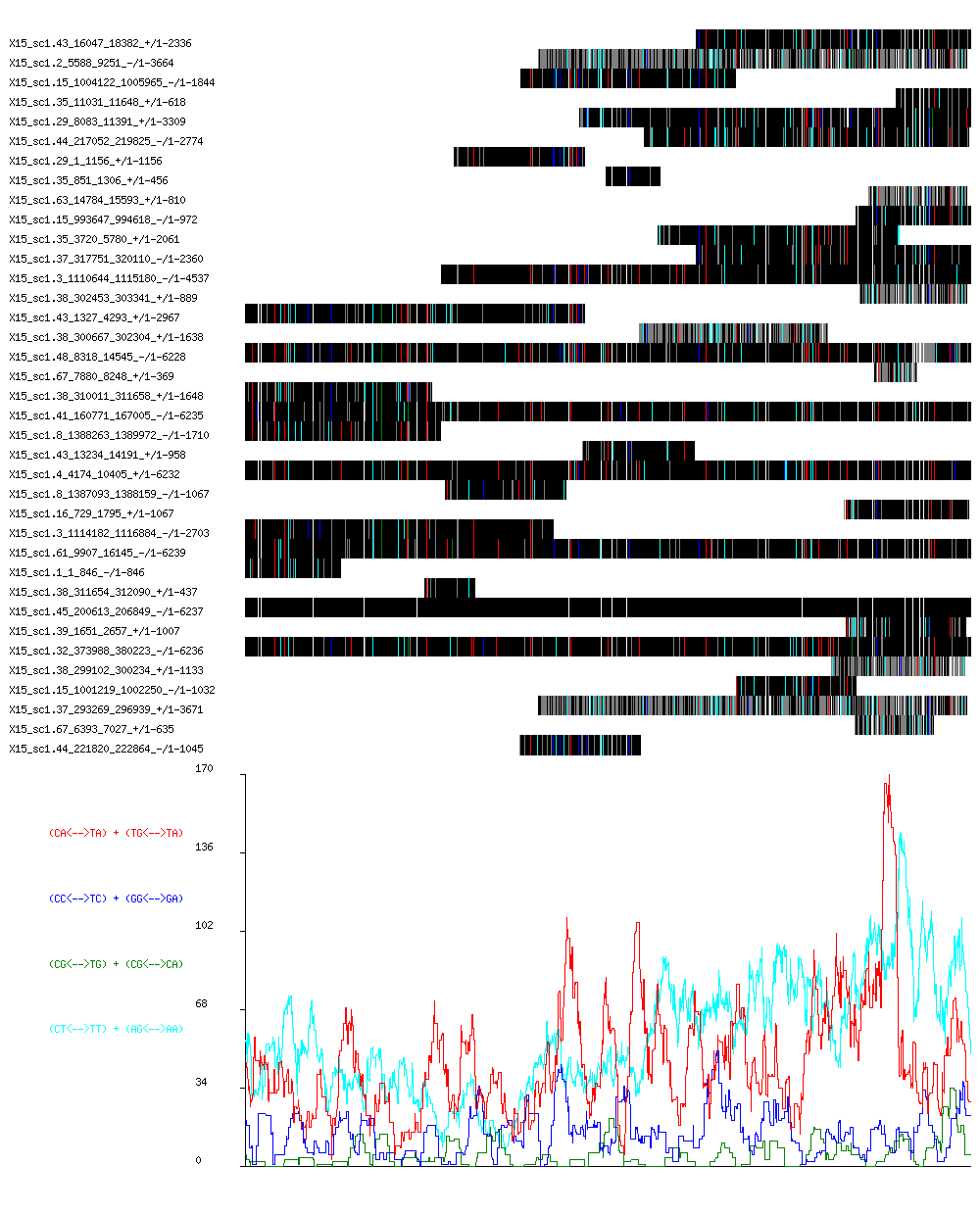

Supplement: Additional file 6 — List of predicted functional RecQ helicases in the S. nodorum genome. [file 1471-2164-11-655-S6.ZIP › highestgc/x15_aln_2009_9_24_11_18_04_X15_sc1.45_200613_206849_-_1-6237.gif]

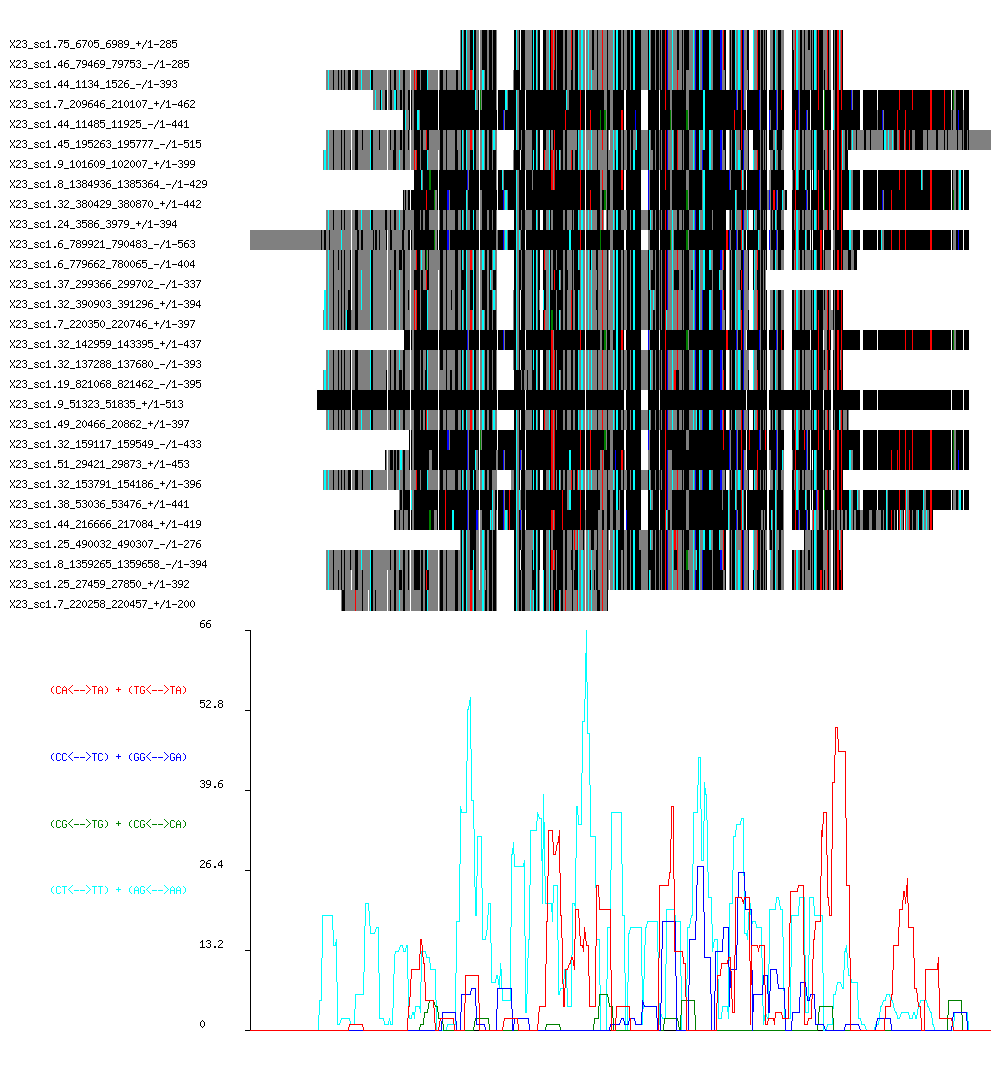

Supplement: Additional file 6 — List of predicted functional RecQ helicases in the S. nodorum genome. [file 1471-2164-11-655-S6.ZIP › highestgc/x23_aln_2009_9_24_11_18_36_X23_sc1.9_51323_51835_+_1-513.gif]

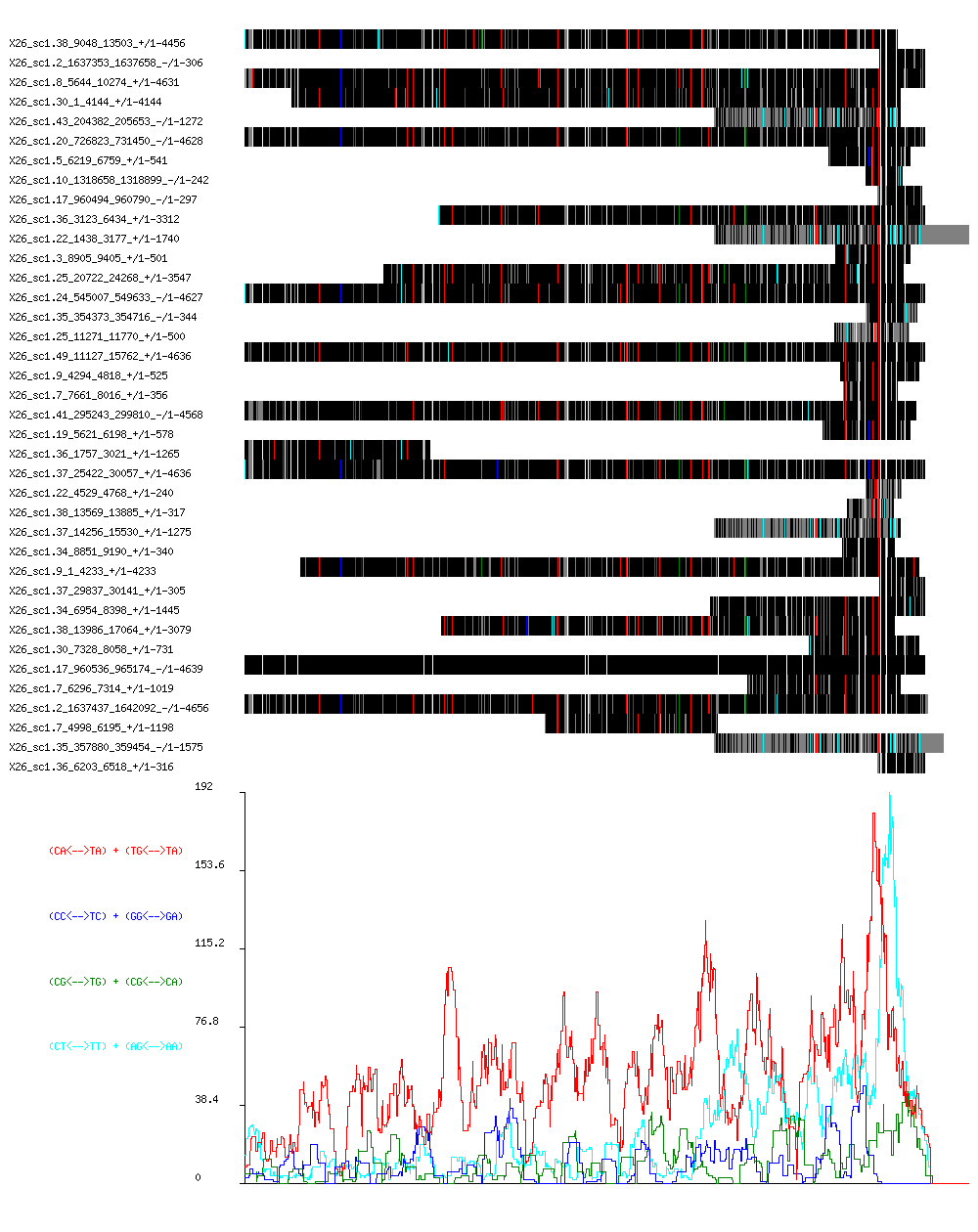

Supplement: Additional file 6 — List of predicted functional RecQ helicases in the S. nodorum genome. [file 1471-2164-11-655-S6.ZIP › highestgc/x26_aln_2009_9_24_11_18_39_X26_sc1.17_960536_965174_-_1-4639.gif]

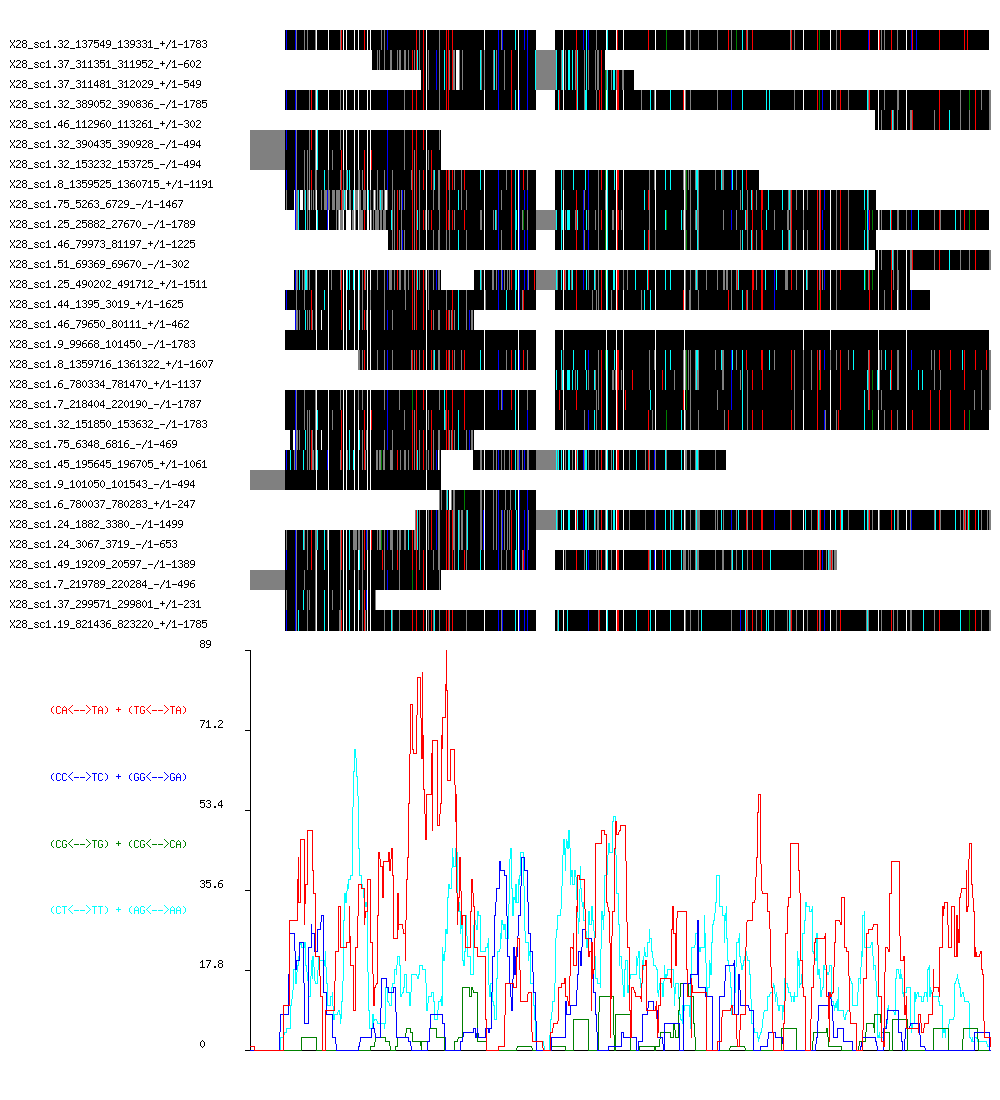

Supplement: Additional file 6 — List of predicted functional RecQ helicases in the S. nodorum genome. [file 1471-2164-11-655-S6.ZIP › highestgc/x28_aln_2009_9_24_11_19_03_X28_sc1.9_99668_101450_-_1-1783.gif]

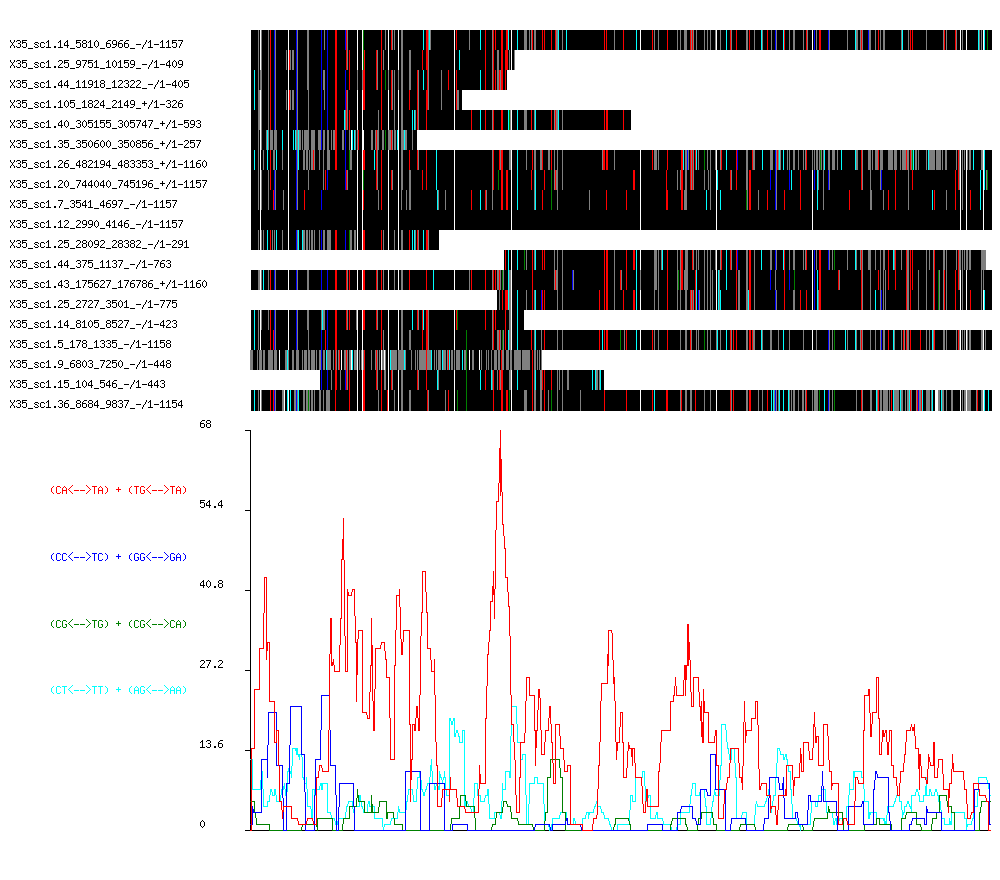

Supplement: Additional file 6 — List of predicted functional RecQ helicases in the S. nodorum genome. [file 1471-2164-11-655-S6.ZIP › highestgc/x35_aln_2009_9_24_11_23_53_X35_sc1.12_2990_4146_-_1-1157.gif]

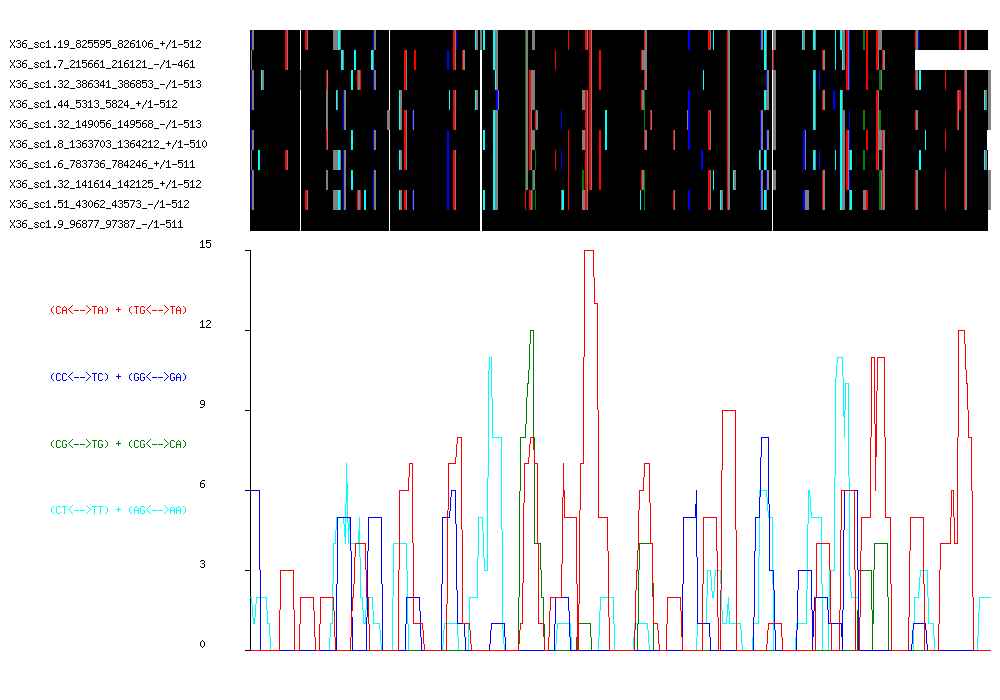

Supplement: Additional file 6 — List of predicted functional RecQ helicases in the S. nodorum genome. [file 1471-2164-11-655-S6.ZIP › highestgc/x36_aln_2009_9_24_11_23_56_X36_sc1.9_96877_97387_-_1-511.gif]

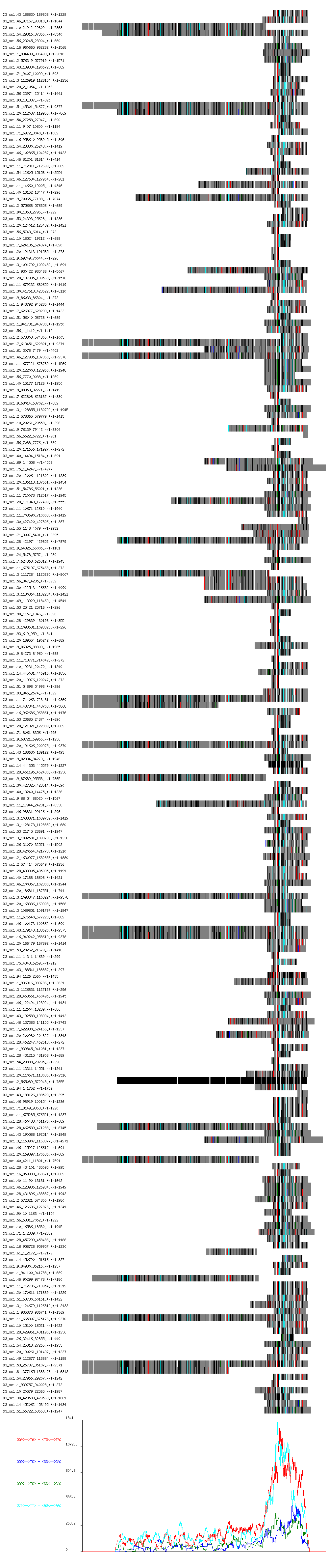

Supplement: Additional file 6 — List of predicted functional RecQ helicases in the S. nodorum genome. [file 1471-2164-11-655-S6.ZIP › highestgc/x3_aln_2009_9_24_11_19_11_X3_sc1.2_565089_572943_+_1-7855.gif]

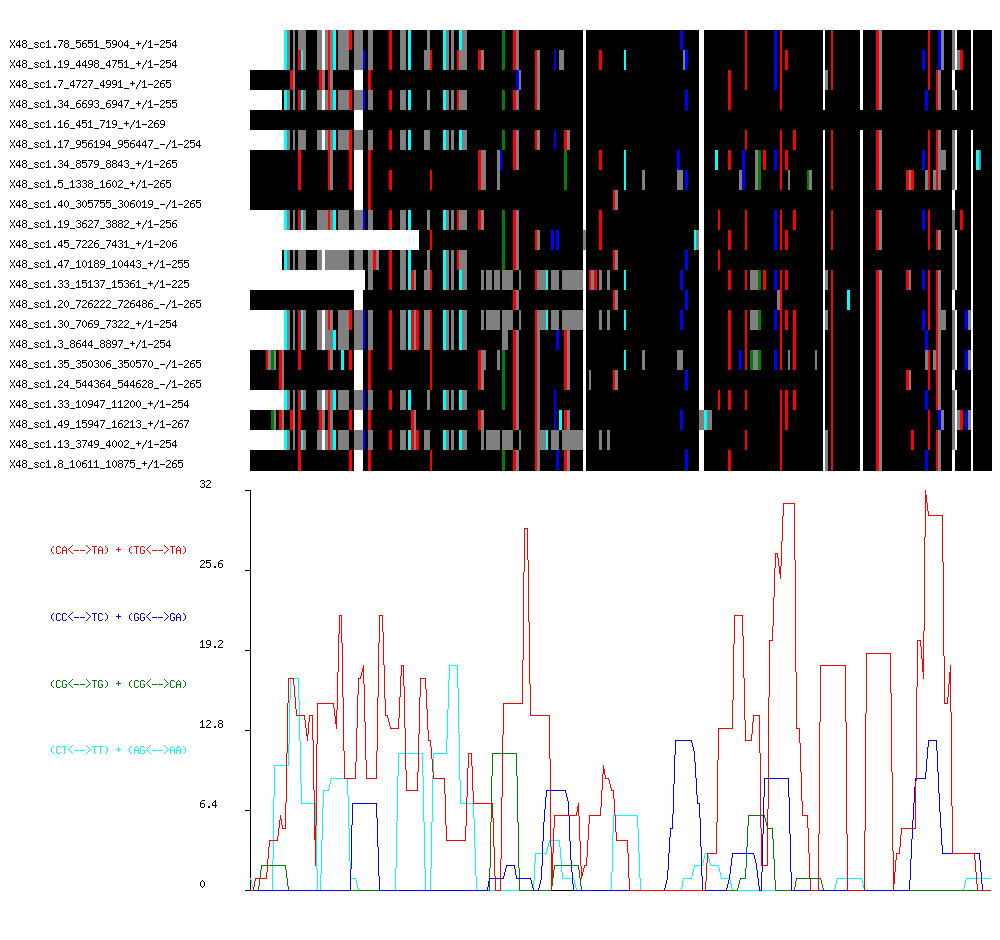

Supplement: Additional file 6 — List of predicted functional RecQ helicases in the S. nodorum genome. [file 1471-2164-11-655-S6.ZIP › highestgc/x48_aln_2009_9_24_11_23_57_X48_sc1.16_451_719_+_1-269.gif]

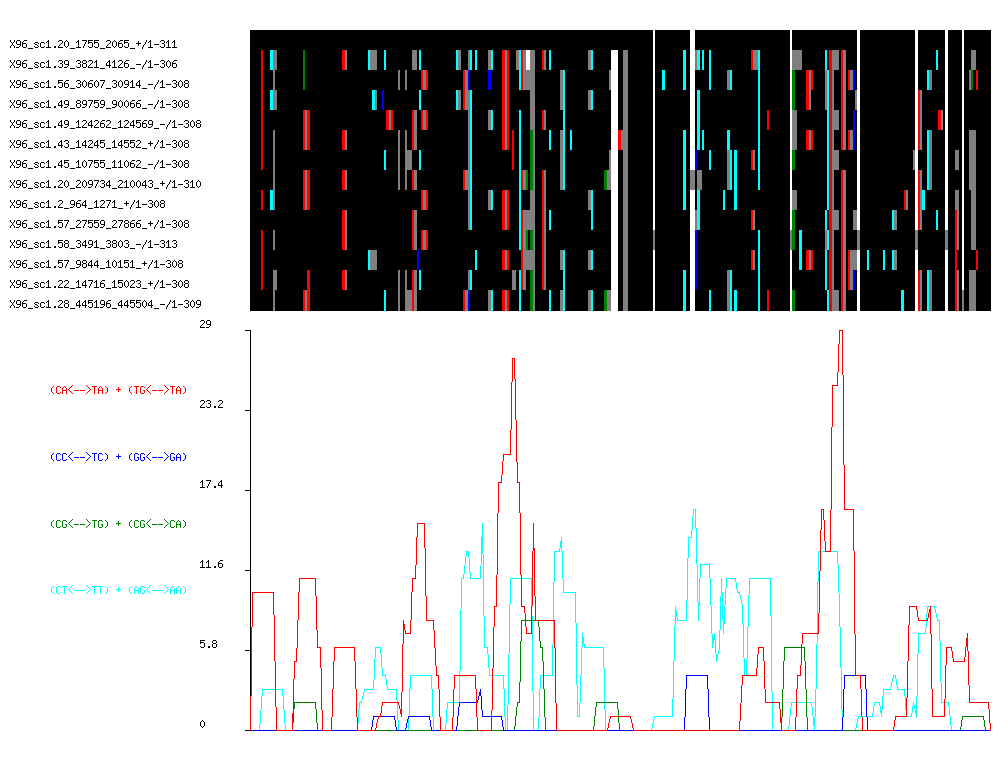

Supplement: Additional file 6 — List of predicted functional RecQ helicases in the S. nodorum genome. [file 1471-2164-11-655-S6.ZIP › highestgc/x96_aln_2009_9_24_11_23_59_X96_sc1.20_1755_2065_+_1-311.gif]

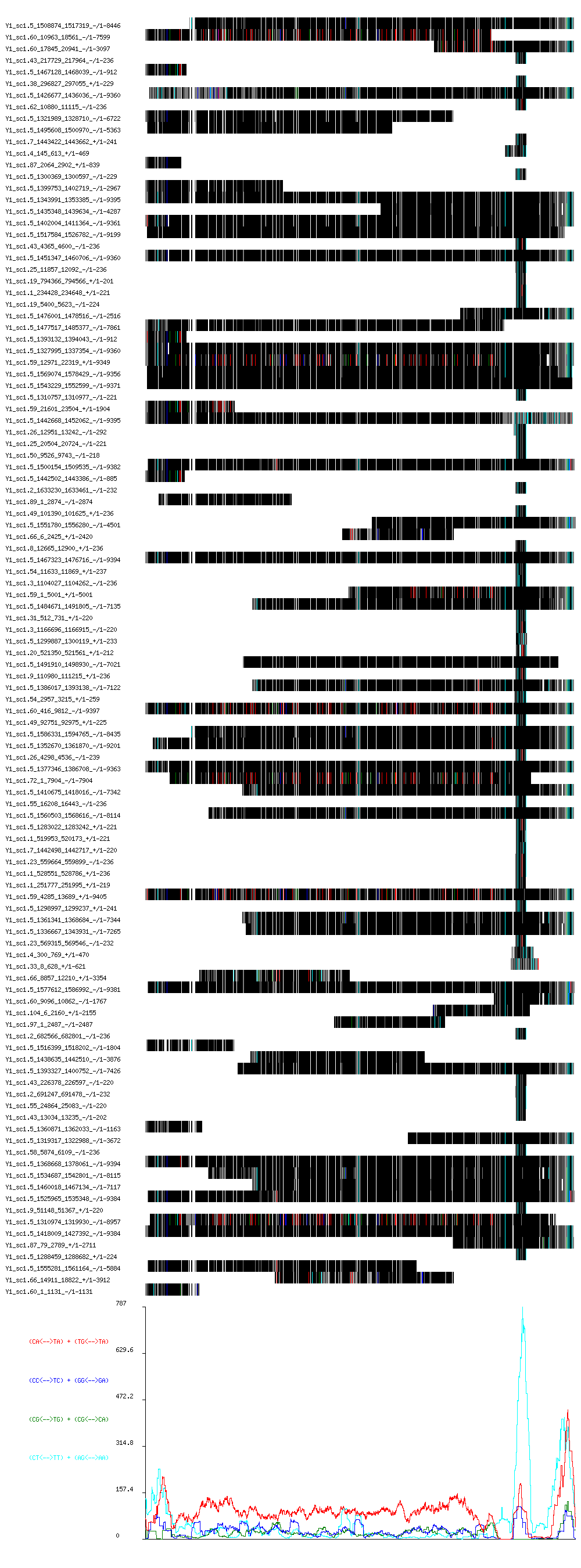

Supplement: Additional file 6 — List of predicted functional RecQ helicases in the S. nodorum genome. [file 1471-2164-11-655-S6.ZIP › highestgc/y1_aln_2009_9_24_11_24_00_Y1_sc1.5_1543229_1552599_-_1-9371.gif]

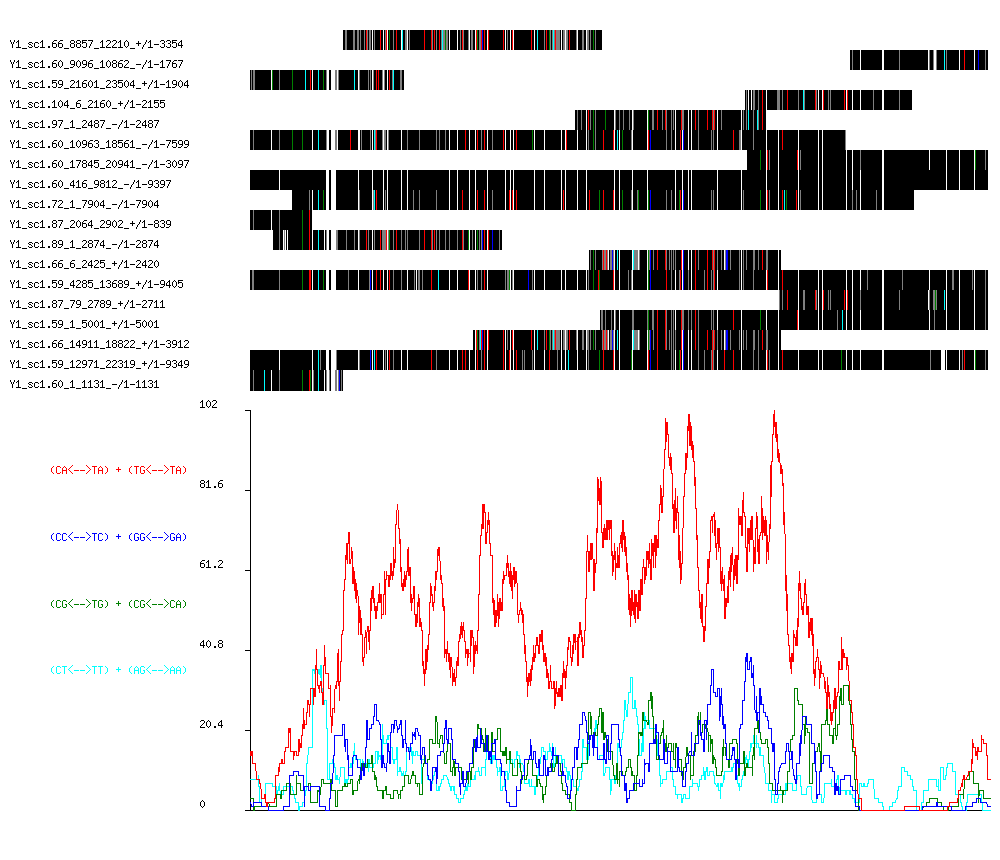

Supplement: Additional file 6 — List of predicted functional RecQ helicases in the S. nodorum genome. [file 1471-2164-11-655-S6.ZIP › highestgc/y1_long_aln_2009_9_24_11_12_51_Y1_sc1.60_416_9812_-_1-9397.gif]

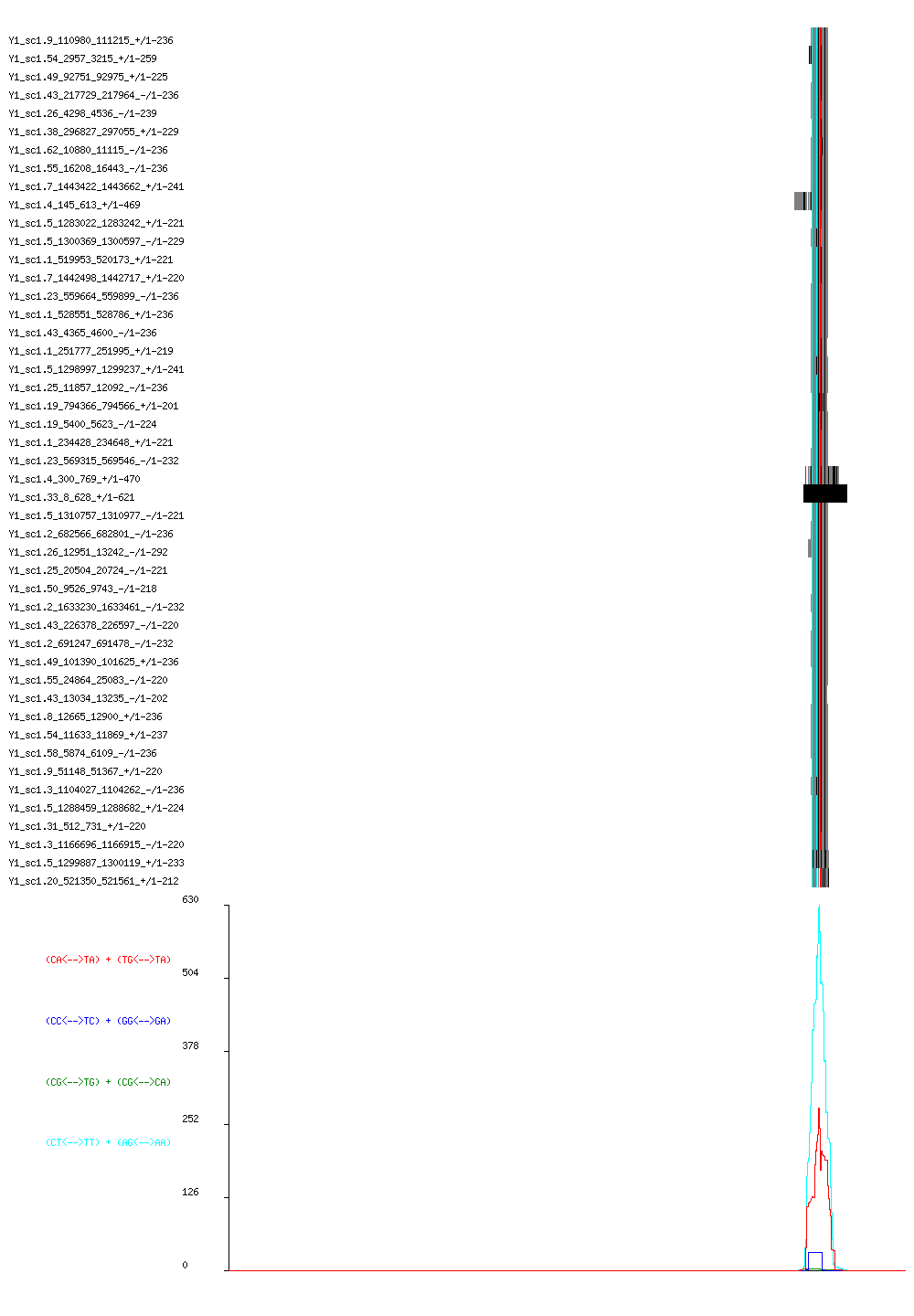

Supplement: Additional file 6 — List of predicted functional RecQ helicases in the S. nodorum genome. [file 1471-2164-11-655-S6.ZIP › highestgc/y1_short_aln_2009_9_24_11_11_57_Y1_sc1.33_8_628_+_1-621.gif]

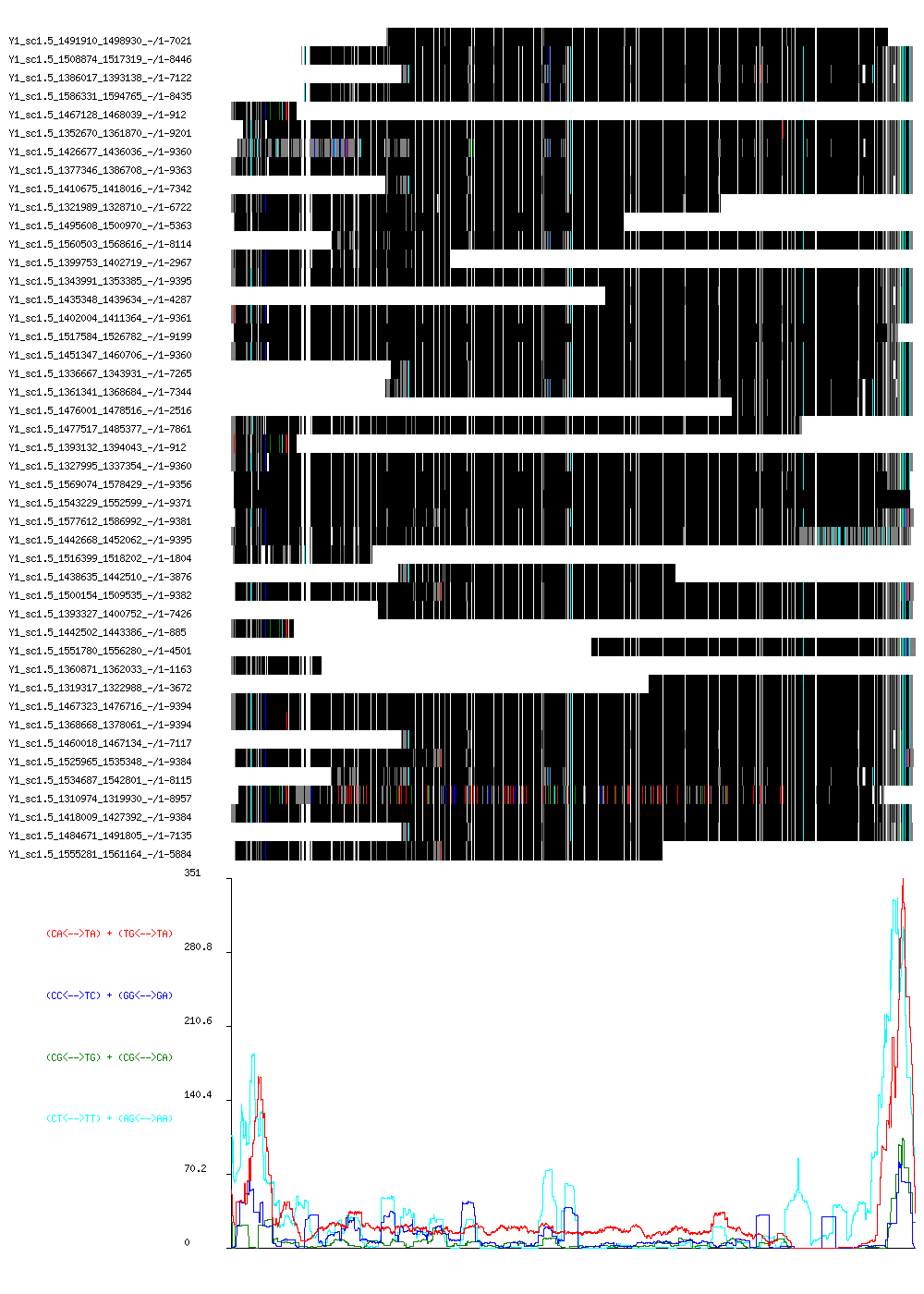

Supplement: Additional file 6 — List of predicted functional RecQ helicases in the S. nodorum genome. [file 1471-2164-11-655-S6.ZIP › highestgc/y1_tandem_aln_2009_9_24_11_10_59_Y1_sc1.5_1543229_1552599_-_1-9371.gif]
